# Associations of lipids and lipid-lowering drugs with risk of stroke: a Mendelian randomization study

**DOI:** 10.3389/fneur.2023.1185986

**Published:** 2023-07-17

**Authors:** Hao Qin, Fan Yang, Haitao Zhao, Jinchuan Zhao, Siyuan Lin, Yinshuai Shang, Chaoling Zhang, Pengfei Hao, Xianfeng Zhang

**Affiliations:** ^1^Department of Neurosurgery, The First Hospital of Jilin University, Changchun, China; ^2^Department of Anesthesiology, The First Hospital of Jilin University, Changchun, China; ^3^Department of Neurosurgery, Third Hospital of Shanxi Medical University, Shanxi Bethune Hospital, Shanxi Academy of Medical Sciences, Tongji Shanxi Hospital, Taiyuan, China

**Keywords:** lipids, lipid-lowering drugs, stroke, causality, Mendelian randomization

## Abstract

**Background:**

Stroke is a leading cause of death worldwide, but it is unclear whether circulating lipids and lipid-lowering drugs are causally associated with stroke and its subtypes.

**Methods:**

We used two-sample Mendelian randomization (MR) to examine the effects of blood lipids and lipid-lowering drugs on stroke and its subtypes.

**Results:**

The inverse variance weighted Mendelian randomization (IVW-MR) revealed the low-density lipoprotein cholesterol (LDL-C) (OR, 1.46; 95% CI, 1.17–1.83; *p* = 0.0008) and apolipoprotein B (apoB) (OR, 1.46; 95% CI, 1.21–1.77; *p* = 0.0001) was positively correlated with large artery stroke (LAS). However, no causal effect was found in LDL-C and apoB on LAS risk when we conducted mvMR. The IVW-MR also found a suggestive evidence that decreased LDL-C levels mediated by the PCSK9 (proprotein convertase subtilisin-kexin type 9) gene were associated with a reduced risk of any stroke (AS) (OR, 1.31; 95% CI, 1.13–1.52; *p* = 0.0003), any ischemic stroke (AIS) (OR, 1.29; 95% CI, 1.10–1.51; *p* = 0.001), and LAS (OR, 1.73; 95% CI, 1.15–2.59; *p* = 0.008), while NPC1L1 (Niemann-Pick C1-like protein)-mediated LDL-C levels were associated with a higher risk of small vessel stroke (SVS) (OR, 6.10; 95% CI, 2.13–17.43; *p* = 0.0008). The SMR revealed that expression of PCSK9 was associated with risk of AS (OR, 1.15; 95% CI, 1.03–1.28; *p* = 0.01), AIS (OR, 1.02; 95% CI, 1.14–1.29; *p* = 0.03), cardioembolic stroke (CES) (OR, 1.28; 95% CI, 1.01–1.61; *p* = 0.04). And, a significant association was found between the expression of NPC1L1 and the risk of SVS (OR, 1.15; 95% CI, 1.00–1.32; *p* = 0.04).

**Conclusion:**

We cautiously find that LDL-C and apoB was positively correlated with LAS. These findings suggest that the reducing LDL-C levels could be an effective prevention strategy for reducing the risk of stroke.

## Introduction

Stroke is major causes of death and disability globally, with ischemic stroke (IS) accounting for 80% of cases. To address the increasing incidence of stroke, effective prevention strategies targeting modifiable risk factors are urgently needed. Although total blood circulating lipids levels are a well-established risk factor for cardiovascular disease, the association with stroke remains unclear. Several observational studies have failed to find a clear association (1–7), including the MRFIT survey, which found elevated total cholesterol to be associated with death from ischemic stroke (*p* = 0.007) and low cholesterol (<160 mg/dL) to be associated with death from hemorrhagic stroke (*p* = 0.05) (1). Furthermore, cholesterol was found to be positively associated with non-hemorrhagic stroke death in women in the Women’s Pooling Project, but only in women younger than 55 years and more strongly in black women (2). The Atherosclerosis in Communities Study observed 14,175 healthy, middle-aged men and women for 10 years and showed no significant association between cholesterol and ischemic stroke (5).

Statins are the most effective and widely used lipid-lowering drugs, with numerous randomized studies demonstrating their benefits in lowering cholesterol in patients with cardiovascular disease. However, most of these trials were designed to study coronary events as the primary outcome and cerebrovascular events as a secondary endpoint or as a component of a composite primary endpoint. Given the weak observational data on the association between cholesterol and stroke, the conclusion that statins are effective in preventing stroke may be due to the pleiotropic effects of these drugs (8, 9).

Mendelian randomization (MR) (10) is a statistical method that utilizes genetic variation as an instrumental variable to assess the causal relationship between exposure factors and outcomes. This method is akin to a “genetically randomized controlled experiment” in that genetic variants are randomly assigned to offspring at the time of conception, which minimizes the risk of confounding factors and reverse causality. Using MR, researchers can investigate whether genetic variants associated with a particular exposure factor (such as lipid levels) are causally related to a specific outcome (such as stroke), providing stronger evidence for causality than traditional observational studies. In this study, MR analyses were performed to assess the causal relationship between circulating lipid-related traits and statins and stroke using the summary GWAS data. I hope our study can provide some clues for prevention of stroke.

## Materials and methods

### Study design

First, univariate MR (uvMR) analyses were used to estimate the causal effects of circulating lipid-related traits on stroke using genetically predicted high-density lipoprotein cholesterol (HDL-C), LDL-C, triglycerides (TG), apolipoprotein A-I (apoA-I), and apolipoprotein B (apoB) levels as exposures and risk of stroke as outcomes based on summary-level genome-wide association study (GWAS) datasets. Second, we conducted multivariable MR (mvMR), which is a statistical approach that allows for the association of single nucleotide polymorphisms (SNPs) with multiple phenotypes to be incorporated into the analysis, permitting an estimation of the direct effect of each phenotype on the outcome. For the multivariable MR analyses, we fitted a model with apolipoprotein B and LDL cholesterol to identify which one or more traits appeared to be responsible for the effect of lipid-related traits on risk of stroke. Third, we conducted summary-data-based MR (SMR) analysis to investigate the association of gene expression from eQTL studies, in which the most significant cis-eQTL SNP was selected as a genetic instrument for the target gene expression [HMGCR (HMG-CoA reductase), PCSK9 (proprotein convertase subtilisin-kexin type 9), NPC1L1 (Niemann-Pick C1-like protein), and APOB (apolipoprotein B-100)] of each approved lipid-lowering drug, with stroke using summary data from GWAS. Third, we used genetic variants related to LDL-C mediated by these target genes as instruments to proxy the exposure of lipid-lowering drugs and applied a two-sample MR method to explore the association between lipid-lowering drugs and stroke risk. Finally, to validate our selection of instrumental variables (IVs), positive control analyses were performed for validation of both genetic instruments.

To account for multiple testing, Bonferroni correction was used to adjust the thresholds of significance level, thus strong evidence was suggested for *p* < 0.005 (five exposures and five outcomes) and suggestive evidence of 0.005 ≤ *p* < 0.05.

### Data sources and identifying genetic instruments

Detailed information on the summarized data sources for the instrumental variables is presented in Table 1.

**Table 1:** Information of eQTL and GWAS summary data.

Characteristic	Resource	Sample size	Population ancestry	PMID
Lipid-related traits				
LDL-C	UK Biobank	440,546	European	32203549
HDL-C	UK Biobank	403,943	European	32203549
TG	UK Biobank	441,016	European	32203549
apolipoprotein A-I (apoA-I)	UK Biobank	393,193	European	32203549
apolipoprotein B (apoB)	UK Biobank	439,214	European	32203549
eQTL data				
eQTL for *HMGCR*	eQTLGen Consortium	Whole blood: 31,684	Predominantly European	–
eQTL in *PCSK9*, *NPC1L1*, and *APOB*	GTExV8	Whole blood: 755 Adipose subcutaneous: 663	Predominantly European	29022597
GWAS summary data				
AS	MEGASTROKE Consortium	67,162	European	29531354
AIS	MEGASTROKE Consortium	60,341	European	29531354
CES	MEGASTROKE Consortium	9,006	European	29531354
LAS	MEGASTROKE Consortium	6,688	European	29531354
SVS	MEGASTROKE Consortium	11,710	European	29531354

AS, any stroke; AIS, any ischemic stroke; LAS, large artery stroke; CES, cardioembolic stroke; SVS, small vessel stroke.

### GWAS of lipid-related traits

Summary statistical data for LDL-C (*n* = 440,546), HDL-C (*n* = 403,943), TG (*n* = 441,016), and apoA-I (*n* = 393,193) and apoB (*n* = 439,214) were extracted in a meta-analysis of GWAS involving individuals of European ancestry in the UK Biobank (UKB) (11).

First, we included SNP reaching GWAS (GWAS *p* < 5 × 10^−8^) whose minor allele frequency > 0.01. Second, we used the cutoff of the corresponding linkage disequilibrium (LD) value (threshold set at *r*^2^ < 0.001, kb = 10,000) to ensure that the IVs were independent (12). Third, we did not include proxy SNPs and excluded palindromic SNPs. Fourth, we excluded SNPs associated with confounding factors (for example, diabetes, hypertension, smoking, and alcohol consumption) using the PhenoScanner online tool.^1^

Finally, we then evaluated the remaining SNPs’ power using the F statistics for each SNP and calculated a general *F* statistic for all SNPs. SNPs with less statistical power would be removed (*F* statistics <10) (Supplementary Table S1).

### eQTL data

SNPs in 4 gene regions (HMGCR, PCSK9, NPC1L1, and APOB) were used to proxy the effect of lipid-lowering drugs from available eQTLs.

The cis-eQTL summary-level data for gene expression of HMGCR in blood were obtained from the eQTLGen Consortium.^2^ And, the summary data for gene expression of PCSK9 in blood, NPC1L1, and APOB in subcutaneous adipose tissue were selected from GTEx Consortium V8.^3^ We selected cis-eQTL genetic instruments significantly [minor allele frequency (MAF) > 1% and *p* < 5.0 × 10^−8^] associated with the expression of genes within 1 Mb on either side of the encoded gene.

To validate the observed association using the eQTLs as an instrument, we identified IVs by selecting significant SNPs (*p* < 5 × 10^−8^) within 100 kb windows associated with LDL-C level at these four genomic regions from the UKB and the more relaxed threshold of clumping SNPs for independence was used (*r*^2^ < 0.30) to maximize the strength of the instrument for each drug. Finally, 19 SNPs within 100 kb windows from the HMGCR gene were selected for proxying HMGCR inhibitors, 33 SNPs from the PCSK9 gene were identified for PCSK9 inhibitors, 6 SNPs from the NPC1L1 gene were selected for NPC1L1 inhibitor and 31 SNPs from the APOB gene were selected for APOB inhibitors. The details of IVs are presented in Supplementary Table S2.

### GWAS of stroke

The GWAS summary data for stroke were obtained from the MEGASTROKE consortium (13). This study included 29 studies including 40,585 cases and 406,111 controls. The MEGASTROKE consortium defined stroke as rapidly developing signs of focal (or global) disturbance of cerebral function, lasting >24 h or leading to death with no apparent cause other than that of vascular origin. The MEGASTROKE consortium divided stroke into as any stroke (AS), any ischemic stroke (AIS), large artery stroke (LAS), cardioembolic stroke (CES), small vessel stroke (SVS). We restricted the stroke population to European ancestry to minimize population stratification bias. A detailed description of the participants and study design of MEGASTROKE were provided in the original study (13).

## MR analyses

### MR estimates using uvMR

We applied the random effects inverse-variance weighted (IVW) model as the main statistical method (14) to evaluate effect estimates when using genetic variants associated with the exposures as an instrument (for analysis with ≥3 SNPs); otherwise, a fixed-effects model was used. The weighted median is robust to invalid instruments and could generate consistent estimates even when >50% of selected genetic variants are invalid instruments (15). MR-Egger regression, which allows the intercept to be freely estimated as an indicator of average pleiotropic bias, can detect some violations of the standard instrumental variable assumptions, i.e., the InSIDE (InstrumentStrength Independent of Direct Effect) assumption is satisfied (16). In addition, a *p*-value for the MR-Egger intercept greater than 0.05 indicates no horizontal pleiotropic effects. Cochran’s Q test and *I*^2^ statistics was used to assess heterogeneous effects among IVs based on the IVW method (15). The leave-one-out analysis was used to evaluate whether the association was driven by a single SNP (17).

All data analyses for MR were conducted by “TwoSampleMR” package in the R environment (R version 4.1.1).

### MR estimates using mvMR

For the multivariable MR analyses, we fitted a model with apolipoprotein B and LDL cholesterol to identify which one or more traits appeared to be responsible for the effect of lipid-related traits on risk of stroke.

### SMR analyses

We performed SMR to identify associations between gene expression and complex traits using summary data from GWAS and eQTL studies. We also performed the heterogeneity in dependent instruments (HEIDI) test to evaluate the existence of linkage in the observed association. A PHEIDI of <0.05 is considered evidence to support that the observed association could be due to two distinct genetic variants in high linkage disequilibrium with each other. A *p*-value below 0.05 was considered statistically significant. The analyses were conducted in the SMR software tool (version 1.03).

## Results

### uvMR analysis of lipid-related traits on stroke risks via forward MR

We performed univariate Mendelian randomization analyses of circulating lipids and stroke (Table 2).

**Table 2:** IVW-MR association between lipid-related traits levels and stroke and its subtypes.

Exposures	Outcomes	IVs	OR (95% CI)	Beta	SE	*p*	Q statistic_*p*	egger_intercept_*p*
LDL-C	AS	121	1.07 (0.98–1.18)	0.071	0.047	0.13	0.000	0.125
	AIS	121	1.10 (0.99–1.21)	0.094	0.049	0.05	0.000	0.161
	CES	120	1.03 (0.88–1.20)	0.027	0.081	0.74	0.038	0.252
	LAS	121	1.46 (1.17–1.83)	0.382	0.113	0.0008	0.001	0.997
	SVS	121	1.20 (0.99–1.44)	0.179	0.094	0.06	0.068	0.738
HDL-C	AS	272	0.97 (0.91–1.02)	−0.034	0.029	0.24	0.000	0.125
	AIS	273	0.96 (0.90–1.02)	−0.043	0.032	0.17	0.000	0.064
	CES	272	0.93 (0.84–1.04)	−0.069	0.054	0.2	0.436	0.486
	LAS	273	0.88 (0.76–1.02)	−0.125	0.075	0.1	0.017	0.011
	SVS	274	0.86 (0.75–0.99)	−0.148	0.071	0.04	0.003	0.367
TG	AS	223	1.04 (0.98–1.10)	0.036	0.030	0.23	0.002	0.065
	AIS	223	1.05 (0.98–1.11)	0.044	0.032	0.16	0.020	0.037
	CES	223	0.97 (0.87–1.09)	−0.027	0.056	0.63	0.914	0.647
	LAS	224	1.20 (1.03–1.40)	0.186	0.079	0.02	0.025	0.065
	SVS	223	1.10 (0.95–1.26)	0.094	0.071	0.19	0.054	0.027
apoA-I	AS	227	0.95 (0.89–1.01)	−0.055	0.031	0.07	0.000	0.326
	AIS	228	0.95 (0.89–1.01)	−0.054	0.033	0.1	0.000	0.253
	CES	227	0.94 (0.84–1.04)	−0.064	0.055	0.24	0.508	0.625
	LAS	228	0.87 (0.75–1.01)	−0.135	0.076	0.07	0.027	0.155
	SVS	228	0.87 (0.76–0.99)	−0.142	0.071	0.04	0.026	0.380
apoB	AS	144	1.10 (1.02–1.19)	0.097	0.040	0.02	0.000	0.714
	AIS	144	1.12 (1.03–1.22)	0.115	0.042	0.007	0.000	0.768
	CES	143	1.04 (0.89–1.21)	0.037	0.078	0.64	0.000	0.522
	LAS	144	1.46 (1.21–1.77)	0.380	0.098	0.0001	0.000	0.381
	SVS	144	1.12 (0.97–1.30)	0.117	0.076	0.12	0.322	0.063

**Figure 1:**
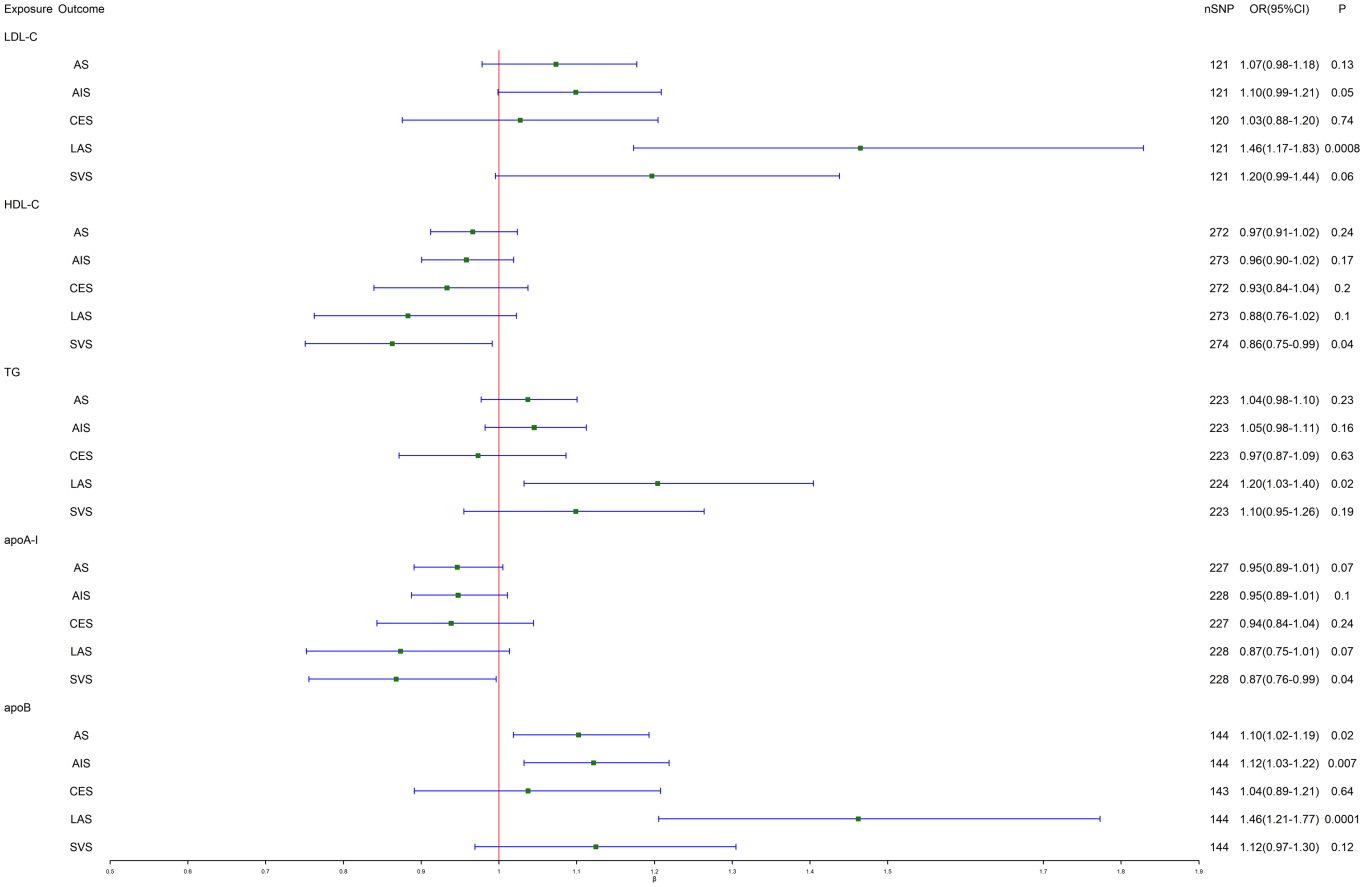
Inverse-variance-weighted Mendelian randomization (IVW-MR) association between lipid-related traits and stroke. LDL-C, low-density lipoprotein cholesterol; HDL-C, high-density lipoprotein cholesterol; TG, triglyceride; apoA-I, apolipoprotein A-I; apoB, apolipoprotein B; AS, any stroke; AIS, any ischemic stroke; CES, cardioembolic stroke; LAS, large artery stroke; SVS, small vessel stroke.

As shown in Figure 1 and Table 2, IVW-MR analysis found a strong evidence for a causal effect of genetically predicted LDL-C (OR, 1.46; 95% CI, 1.17–1.83; *p* = 0.0008) and apoB (OR, 1.46; 95% CI, 1.21–1.77; *p* = 0.0001) on LAS. Heterogeneity but no pleiotropy was detected based on the Q test (*p* < 0.05) and MR-Egger intercept test (*p* > 0.05). Furthermore, suggestive evidence indicated that genetically predicted TG (OR, 1.20; 95% CI, 1.03–1.40; *p* = 0.02) was related to the higher risk of LAS, genetically predicted apoB was related to the higher risk of AS (OR, 1.10; 95% CI, 1.02–1.19; *p* = 0.02) and AIS (OR, 1.12; 95% CI, 1.03–1.22; *p* = 0.007), and genetically predicted HDL-C (OR, 0.86; 95% CI, 0.75–0.99; *p* = 0.04) and apoA-I (OR, 0.87; 95% CI, 0.76–0.99; *p* = 0.04) are associated with the lower risk of SVS. Likewise, heterogeneity but no pleiotropy was detected. The scatter plots are presented in Supplementary Figures S6A–S10E. The leave-one-out method indicated that no SNP was substantially driving the association between lipid-related traits and stroke risks (Supplementary Figures S1A–S5E). The funnel plots are presented in Supplementary Figures S11A–S15E.

### mvMR analysis

When LDL-C and apoB were assessed together in multivariable MR, no causal effect was found in LDL-C (OR, 1.11; 95% CI, 0.47–2.60; *p* = 0.81) and apoB (OR, 1.30; 95% CI, 0.50–3.35; *p* = 0.60) on LAS risk. Similarly, heterogeneity but no pleiotropy was detected (Table 3).

**Table 3:** mvMR association between LDL cholesterol and apolipoprotein B and LAS.

Exposure	Outcome	IVs	Method	b	SE	*p*-value	Q statistic_*p*	egger_intercept_*p*
LDL cholesterol Apolipoprotein B	LAS	162	IVW	0.10	0.44	0.81	3.40E-06	–
		162	IVW	0.26	0.49	0.60	3.40E-06	–
		162	Weighted median	0.54	0.58	0.36	–	–
		162	Weighted median	−0.23	0.63	0.71	–	–
		162	MR-Egger	0.10	0.44	0.82	2.66E-06	0.97
		162	MR-Egger	0.26	0.51	0.61	2.66E-06	0.97

### SMR analyses

The SMR method was conducted to assess the association between the expression of HMGCR, PCSK9, NPC1L1, and APOB and stroke outcome. We obtained 921, 24, 11, and 161 SNPs for cis-eQTL results from eQTLGen or GTEx Consortium about the drug target genes HMGCR, PCSK9, NPC1L1, and APOB, respectively. Next, SMR analyses were performed to use the most significant cis-eQTL SNP (rs6453133, rs472495, rs41279633, and rs4665179) as an IV for the target gene of each lipid-lowering drug.

The results of SMR analysis suggested a significant association between the expression of PCSK9 and the risk of AS (OR, 1.15; 95% CI, 1.03–1.28; *p* = 0.01), AIS (OR, 1.02; 95% CI, 1.14–1.29; *p* = 0.03), and CES (OR, 1.28; 95% CI, 1.01–1.61; *p* = 0.04). Besides, a significant association also was found between the expression of NPC1L1 and the risk of SVS (OR, 1.15; 95% CI, 1.00–1.32; *p* = 0.04). The HEIDI test suggested that all observed associations were not due to a linkage (*p* > 0.05) (Table 4).

**Table 4:** SMR association between expression of gene HMGCR, PCSK9, NPC1L1 or APOB and stroke and its subtypes.

Gene	Top eQTL SNP	SNP chromosome	SNP base pair	Effect allele	Other allele	Effect allele frequency	GWAS association				SMR association			HEIDI test	
							Outcome	Beta	SE	*p*-value	Beta	SE	*p*-value	*p*-value	Number of SNPs
HMGCR	rs6453133	5	74,692,776	G	A	0.286	AS	−0.015	0.010	1.38E-01	−0.117	0.079	1.38E-01	2.99E-01	20
HMGCR	rs6453133	5	74,692,776	G	A	0.286	AIS	−0.013	0.011	2.44E-01	−0.099	0.086	2.45E-01	4.59E-01	20
HMGCR	rs6453133	5	74,692,776	G	A	0.286	CES	−0.017	0.021	4.34E-01	−0.129	0.165	4.35E-01	4.63E-01	20
HMGCR	rs6453133	5	74,692,776	G	A	0.286	LAS	−0.007	0.027	8.06E-01	−0.052	0.211	8.06E-01	2.50E-01	20
HMGCR	rs6453133	5	74,692,776	G	A	0.286	SVS	0.043	0.025	8.66E-02	0.335	0.197	8.90E-02	4.31E-01	20
PCSK9	rs472495	1	55,521,313	T	G	0.628	AS	0.026	0.010	9.20E-03	0.139	0.057	1.41E-02	4.18E-01	8
PCSK9	rs472495	1	55,521,313	T	G	0.628	AIS	0.025	0.011	2.17E-02	0.133	0.060	2.76E-02	5.95E-01	8
PCSK9	rs472495	1	55,521,313	T	G	0.628	CES	0.046	0.021	3.01E-02	0.244	0.117	3.75E-02	7.04E-01	8
PCSK9	rs472495	1	55,521,313	T	G	0.628	LAS	0.054	0.027	4.42E-02	0.288	0.149	5.26E-02	9.95E-01	8
PCSK9	rs472495	1	55,521,313	T	G	0.628	SVS	0.037	0.025	1.35E-01	0.200	0.136	1.42E-01	3.78E-01	8
NPC1L1	rs41279633	7	44,580,876	T	G	0.144	AS	0.010	0.013	4.59E-01	0.020	0.027	4.60E-01	8.70E-01	5
NPC1L1	rs41279633	7	44,580,876	T	G	0.144	AIS	0.009	0.014	5.01E-01	0.020	0.029	5.00E-01	6.41E-01	5
NPC1L1	rs41279633	7	44,580,876	T	G	0.144	CES	−0.012	0.027	6.75E-01	−0.024	0.058	6.75E-01	5.18E-01	5
NPC1L1	rs41279633	7	44,580,876	T	G	0.144	LAS	0.069	0.035	4.96E-02	0.145	0.076	5.51E-02	2.25E-01	5
NPC1L1	rs41279633	7	44,580,876	T	G	0.144	SVS	0.067	0.032	3.71E-02	0.141	0.069	4.29E-02	7.53E-01	5
APOB	rs4665179	2	21,321,724	C	T	0.306	AS	0.001	0.010	9.05E-01	0.003	0.024	9.04E-01	1.16E-01	20
APOB	rs4665179	2	21,321,724	C	T	0.306	AIS	0.009	0.011	3.98E-01	0.022	0.026	4.02E-01	3.54E-02	20
APOB	rs4665179	2	21,321,724	C	T	0.306	CES	0.015	0.020	4.62E-01	0.036	0.050	4.64E-01	2.23E-01	20
APOB	rs4665179	2	21,321,724	C	T	0.306	LAS	−0.007	0.027	7.99E-01	−0.017	0.065	7.98E-01	7.08E-01	20
APOB	rs4665179	2	21,321,724	C	T	0.306	SVS	0.017	0.025	5.00E-01	0.040	0.060	5.01E-01	1.75E-01	20

AS, any stroke; AIS, any ischemic stroke; LAS, large artery stroke; CES, cardioembolic stroke; SVS, small vessel stroke.

### Causal effect of LDL-C level mediated by target genes on stroke via MR analyses

We selected 19, 33, 6, and 31 SNPs within or near the genes HMGCR, PCSK9, NPC1L1, and APOB from summary statistical data for LDL-C level from the UKB, respectively (Table 5).

**Table 5:** IVW-MR association between LDL cholesterol mediated by gene HMGCR, PCSK9, NPC1L1, or APOB and stroke and its subtypes.

Exposure	Outcomes	IVs	OR (95% CI)	Beta	SE	*p*	Q statistic	Q_df	*I*^2^ (%)	Q statistic_*p*	egger_intercept_*p*
HMGCR	AS	16	0.87 (0.71–1.07)	−0.136	0.105	0.2	18.71	15	20.0	0.227	0.150
	AIS	16	0.90 (0.73–1.10)	−0.109	0.102	0.29	14.26	15	0	0.506	0.172
	CES	16	0.90 (0.61–1.33)	−0.102	0.197	0.6	15.11	15	0.7	0.443	0.441
	LAS	16	0.82 (0.48–1.41)	−0.196	0.275	0.48	18.00	15	16.7	0.263	0.977
	SVS	16	2.35 (1.49–3.72)	0.856	0.234	0.0002	6.72	15	0	0.965	0.622
PCSK9	AS	28	1.31 (1.13–1.52)	0.270	0.074	0.0003	15.52	27	0	0.961	0.125
	AIS	28	1.29 (1.10–1.51)	0.257	0.081	0.001	15.19	27	0	0.967	0.260
	CES	28	1.37 (0.99–1.88)	0.311	0.163	0.06	23.18	27	0	0.675	0.378
	LAS	28	1.73 (1.15–2.59)	0.547	0.207	0.008	16.95	27	0	0.932	0.195
	SVS	28	1.06 (0.73–1.55)	0.061	0.193	0.75	28.94	27	6.7	0.364	0.315
NPC1L1	AS	6	1.25 (0.82–1.89)	0.222	0.212	0.3	0.67	5	0	0.985	0.985
	AIS	6	1.26 (0.80–1.98)	0.230	0.232	0.32	0.52	5	0	0.992	0.916
	CES	6	0.61 (0.25–1.47)	−0.498	0.452	0.27	0.94	5	0	0.967	0.960
	LAS	6	3.01 (0.94–9.65)	1.103	0.594	0.06	5.18	5	3.5	0.395	0.208
	SVS	6	6.10 (2.13–17.44)	1.808	0.536	0.0007	1.79	5	0	0.877	0.545
APOB	AS	27	0.95 (0.87–1.05)	−0.047	0.049	0.33	20.53	26	0	0.766	0.140
	AIS	27	1.00 (0.91–1.12)	0.006	0.053	0.91	24.30	26	0	0.559	0.123
	CES	27	0.98 (0.79–1.22)	−0.021	0.111	0.85	31.08	26	16.3	0.225	0.248
	LAS	27	0.88 (0.68–1.13)	−0.130	0.130	0.32	21.37	26	0	0.723	0.491
	SVS	27	1.03 (0.79–1.34)	0.027	0.136	0.32	32.78	26	20.7	0.169	0.435

*I*^2^ = (Q statistic − Q_df) / Q statistic × 100%.

**Figure 2:**
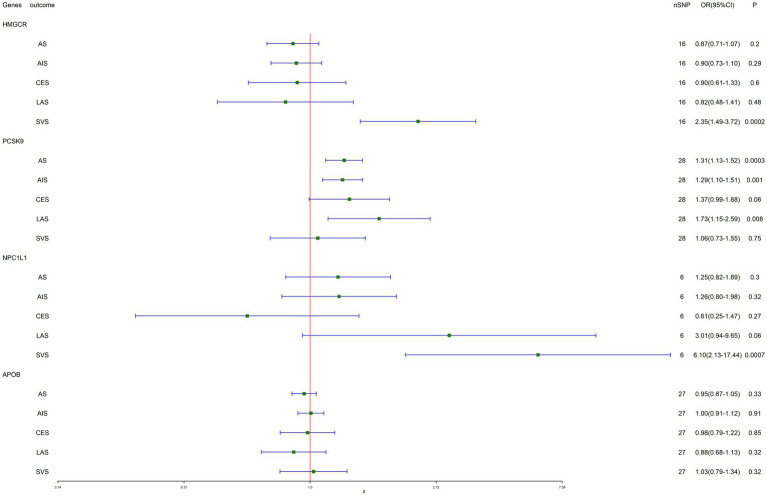
Inverse-variance-weighted Mendelian randomization (IVW-MR) association between low-density lipoprotein (LDL) cholesterol mediated by gene HMGCR, PCSK9, NPC1L1, or APOB and stroke.

The results of MR analysis provided evidence that the decreased LDL-C level mediated by the PCSK9 gene reduced the risk of AS (OR, 1.31; 95% CI, 1.13–1.52; *p* = 0.0003), AIS (OR, 1.29; 95% CI, 1.10–1.51; *p* = 0.001), and LAS (OR, 1.73; 95% CI, 1.15–2.59; *p* = 0.008). In addition, IVW-MR analysis also found a suggestive evidence for the association between NPC1L1-mediated LDL cholesterol and the risk of SVS (OR, 6.10; 95% CI, 2.13–17.43; *p* = 0.0008), further supporting a possible protective effect of NPC1L1 inhibitors against SVS. Similarly, strong evidence was observed between HMGCR-mediated LDL cholesterol and the risk of SVS (OR. 2.35, 95% CI, 1.49–3.72; *p* = 0.0002). There is little evidence of heterogeneity and horizontal pleiotropy for all reported results (all *p* > 0.05; Figure 2).

### Positive control analysis

To validate our selection of IVs, the positive control study showed significant associations between exposure to each drug and LDL cholesterol when using eQTLs-proposed instruments (Table 6), as well as between exposure to each drug and coronary heart disease when using LDL cholesterol GWAS-proposed instruments (Table 7).

**Table 6:** SMR association between expression of gene HMGCR, PCSK9, or NPC1L1 and LDL cholesterol level.

Exposure	OR	95%CI	*p* value	Tissue
HMGCR	1.46	1.37–1.55	2.99E-34	Blood
PCSK9	1.26	1.18–1.34	3.24E-12	Blood
NPC1L1	1.08	1.06–1.10	7.51E-13	Adipose subcutaneous
APOB	1.20	1.16–1.24	2.13E-26	Adipose subcutaneous

**Table 7:** IVW-MR association between LDL cholesterol mediated by gene HMGCR, PCSK9, NPC1L1 or APOB and coronary heart disease.

Exposure	OR	95%CI	*p* value
HMGCR	1.62	1.36–1.93	8.29E-08
PCSK9	2.30	1.95–2.70	7.20E-24
NPC1L1	2.19	1.41–3.39	4.92E-04
APOB	1.41	1.24–1.61	2.42E-07

## Discussion

Our study aimed to evaluate the potential causal effects of genetically determined lipid-related traits and lipid-lowering drugs on stroke risk in a European population using uvMR, mvMR, and SMR. Firstly, our study provided suggestive evidence of a link between LDL cholesterol levels and LAS risk, using uvMR. However, no causal effect was found in LDL-C and apoB on LAS risk when we conducted mvMR, so we should be cautious about LDL-C and apoB can increase the risk of LAS. Secondly, both SMR and uvMR suggested a positive association between PCSK9 expression and PCSK9-mediated LDL cholesterol levels with the risk of AS and AIS, and between NPC1L1 expression and NPC1L1-mediated LDL cholesterol levels with the risk of SVS. Additionally, we found suggestive evidence of a positive association between PCSK9 expression and CES, but this was not supported when using PCSK9-mediated LDL cholesterol as an instrument. Strong evidence was observed for the protective effect of HMGCR-mediated LDL cholesterol on SVS, but there was no evidence for the association of HMGCR expression and SVS.

There have been numerous studies examining the association between lipids and stroke, but the conclusions have been inconclusive (18–22). For instance, the City of Copenhagen Heart Study found a positive association between total cholesterol and the risk of non-hemorrhagic stroke, but only for levels greater than 309 mg/dL, which corresponded to more than 5% of the study cohort (4). On the other hand, several epidemiological studies have reported a significant negative association between HDL-C and stroke (4, 23). Laloux et al.’s analysis of 240 consecutive patients revealed that low HDL-C levels were more common in large-vessel disease relative to other stroke subtypes (24). Additionally, an analysis of patients with coronary disease found that elevated triglycerides significantly increase the risk of first-ever ischemic stroke (25). However, data on the relationship between triglycerides and ischemic stroke are limited and inconclusive. The inconsistent results from conventional studies can be attributed to confounding factors and reverse causality, but Mendelian randomization analysis can effectively avoid these issues.

Proprotein convertase subtilisin/kexin type 9 (PCSK9) is a crucial regulator of low-density lipoprotein (LDL) receptor (LDLR) metabolism, which increases the circulation of LDL cholesterol (LDL-C) by degrading LDLR lysosomally, preventing receptor recycling to the cell membrane, and reducing cellular uptake of LDL-C from plasma (26). Studies have revealed that patients with gain-of-function PCSK9 variants have a higher LDL-C and an increased risk of ischemic stroke. However, a meta-analysis of the loss-of-function rs11591147 (R46L) PCSK9 variant and several studies showed no association between this variant and ischemic stroke and its subtypes (27–30). In addition, ezetimibe, a pharmacological inhibitor of Niemann–Pick disease type C1-Like 1 (NPC1L1), has been found to attenuate neuronal apoptosis through AMPK dependent autophagy activation after middle cerebral artery occlusion in rats. Autophagy activation has also been shown to have neuroprotective effects in an ischemic stroke model (31). However, many of these studies are subject to confounding bias and reverse causation.

The MR study is a powerful tool in genetic epidemiology that can overcome the limitations of traditional observational studies. In this study, we used genetic variants associated with PCSK9 expression or PCSK9-mediated LDL cholesterol as instruments to proxy the exposure of statins. Our analysis found strong evidence that PCSK9 inhibition could reduce the risk of AS and AIS. Similarly, NPC1L1 inhibition could reduce the risk of SVS. Our study has several strengths. First, we minimized the potential for population stratification bias by focusing on individuals of European ancestry. Second, we employed both uvMR and SMR analyses to evaluate the causal effects of genetically determined lipid-related traits and lipid-lowering drugs on the risk of stroke based on GWAS and eQTL data. Third, we conducted a positive control test to validate the instrumental variables’ strength.

However, this study also has several limitations that need to be considered when interpreting the results. Firstly, the lack of available eQTLs in blood for NPC1L1 and APOB limited our ability to explore the association between NPC1L1 and APOB expression in blood and stroke. Secondly, the generalizability of our results is limited to populations of European ancestry since we only used genetic summary data from this population. Thirdly, although we performed sensitivity analyses to test the assumptions of the MR study, the possibility of confounding bias and/or horizontal pleiotropy cannot be completely ruled out. Fourthly, the use of summary-level data did not allow for subgroup analyses, so further MR studies using individual-level data are necessary to provide more detailed information.

## Conclusion

In conclusion, this study provides genetic evidence that decreasing LDL-C and apoB levels may reduce the risk of LAS, but it is cautious. The results also suggest that statins, which lower LDL-C levels, could be an effective prevention strategy for the risk of stroke. However, these findings need to be confirmed by clinical trials in the future.

## Data availability statement

The original contributions presented in the study are included in the article/Supplementary material, further inquiries can be directed to the corresponding author.

## Ethics statement

Ethical review and approval was not required for the study on human participants in accordance with the local legislation and institutional requirements. Written informed consent from the patients/participants or patients/participants’ legal guardian/next of kin was not required to participate in this study in accordance with the national legislation and the institutional requirements.

## Author contributions

HQ, XZ, and FY: conceptualization, validation, and supervision. HQ: methodology and data curation. HQ and PH: software. HQ, HZ, and JZ: formal analysis. YS, CZ, and SL: resources. HQ and FY: writing—original draft preparation and writing—review & editing. HZ and JZ: visualization. HQ, FY, HZ, JZ, SL, YS, CZ, PH, and XZ commented on the manuscript. All authors contributed to the article and approved the submitted version.

## Funding

The MEGASTROKE project received funding from sources specified at https://www.megastroke.org/acknowledgements.html.

## Conflict of interest

The authors declare that the research was conducted in the absence of any commercial or financial relationships that could be construed as a potential conflict of interest.

## Publisher’s note

All claims expressed in this article are solely those of the authors and do not necessarily represent those of their affiliated organizations, or those of the publisher, the editors and the reviewers. Any product that may be evaluated in this article, or claim that may be made by its manufacturer, is not guaranteed or endorsed by the publisher.

## Footnotes

^1^http://www.phenoscanner.medschl.cam.ac.uk/

^2^https://www.eqtlgen.org/

^3^https://gtexportal.org/
