# Supplementary material for: Associations of lipids and lipid-lowering drugs with risk of stroke: a Mendelian randomization study
Source: Front Neurol. 2023 Jul 17;14:1185986. doi: 10.3389/fneur.2023.1185986 (PMC10390065; doi:10.3389/fneur.2023.1185986)
Supplement: Supplementary file 1 [file Data_Sheet_1.ZIP › Supplementary materials.doc]

### Supplementary Materials

**Supplementary Table S1:** Information of genetic instrumental variants associated with lipid-related traits via uvMR.

**Supplementary Table S2:** Information of genetic instrumental variants associated with LDL cholesterol located within 100 kb windows from gene HMGCR, PCSK9, NPC1L1 or APOB.

**Supplementary Figure S1A:** Leave-one-out permutation analysis of the causal association between LDL-C level and AS;

**Supplementary Figure S1B:** Leave-one-out permutation analysis of the causal association between HDL-C level and AS;

**Supplementary Figure S1C:** Leave-one-out permutation analysis of the causal association between TG level and AS;

**Supplementary Figure S1D:** Leave-one-out permutation analysis of the causal association between apoA-I level and AS;

**Supplementary Figure S1E:** Leave-one-out permutation analysis of the causal association between apoB level and AS;

**Supplementary Figure S2A:** Leave-one-out permutation analysis of the causal association between LDL-C level and AIS;

**Supplementary Figure S2B:** Leave-one-out permutation analysis of the causal association between HDL-C level and AIS;

**Supplementary Figure S2C:** Leave-one-out permutation analysis of the causal association between TG level and ASI;

**Supplementary Figure S2D:** Leave-one-out permutation analysis of the causal association between apoA-I level and AIS;

**Supplementary Figure S2E:** Leave-one-out permutation analysis of the causal association between apoB level and AIS;

**Supplementary Figure S3A:** Leave-one-out permutation analysis of the causal association between LDL-C level and CES;

**Supplementary Figure S3B:** Leave-one-out permutation analysis of the causal association between HDL-C level and CES;

**Supplementary Figure S3C:** Leave-one-out permutation analysis of the causal association between TG level and CES;

**Supplementary Figure S3D:** Leave-one-out permutation analysis of the causal association between apoA-I level and CES;

**Supplementary Figure S3E:** Leave-one-out permutation analysis of the causal association between apoB level and CES;

**Supplementary Figure S4A:** Leave-one-out permutation analysis of the causal association between LDL-C level and LAS;

**Supplementary Figure S4B:** Leave-one-out permutation analysis of the causal association between HDL-C level and LAS;

**Supplementary Figure S4C:** Leave-one-out permutation analysis of the causal association between TG level and LAS;

**Supplementary Figure S4D:** Leave-one-out permutation analysis of the causal association between apoA-I level and LAS;

**Supplementary Figure S4E:** Leave-one-out permutation analysis of the causal association between apoB level and LAS;

**Supplementary Figure S5A:** Leave-one-out permutation analysis of the causal association between LDL-C level and SVS;

**Supplementary Figure S5B:** Leave-one-out permutation analysis of the causal association between HDL-C level and SVS;

**Supplementary Figure S5C:** Leave-one-out permutation analysis of the causal association between TG level and SVS;

**Supplementary Figure S5D:** Leave-one-out permutation analysis of the causal association between apoA-I level and SVS;

**Supplementary Figure S5E:** Leave-one-out permutation analysis of the causal association between apoB level and SVS;

**Supplementary Figure S6A:** Scatter plot showing the association of the SNP effects on LDL-C level against the SNP effects on AS;

**Supplementary Figure S6B:** Scatter plot showing the association of the SNP effects on HDL-C level against the SNP effects on AS;

**Supplementary Figure S6C:** Scatter plot showing the association of the SNP effects on TG levels against the SNP effects on AS;

**Supplementary Figure S6D:** Scatter plot showing the association of the SNP effects on apoA-I level against the SNP effects on AS;

**Supplementary Figure S6E:** Scatter plot showing the association of the SNP effects on the apoB level against the SNP effects on AS;

**Supplementary Figure S7A:** Scatter plot showing the association of the SNP effects on LDL-C level against the SNP effects on AIS;

**Supplementary Figure S7B:** Scatter plot showing the association of the SNP effects on HDL-C level against the SNP effects on AIS;

**Supplementary Figure S7C:** Scatter plot showing the association of the SNP effects on TG levels against the SNP effects on AIS;

**Supplementary Figure S7D:** Scatter plot showing the association of the SNP effects on apoA-I level against the SNP effects on AIS;

**Supplementary Figure S7E:** Scatter plot showing the association of the SNP effects on the apoB level against the SNP effects on AIS;

**Supplementary Figure S8A:** Scatter plot showing the association of the SNP effects on LDL-C level against the SNP effects on CES;

**Supplementary Figure S8B:** Scatter plot showing the association of the SNP effects on HDL-C level against the SNP effects on CES;

**Supplementary Figure S8C:** Scatter plot showing the association of the SNP effects on TG levels against the SNP effects on CES;

**Supplementary Figure S8D:** Scatter plot showing the association of the SNP effects on apoA-I level against the SNP effects on CES;

**Supplementary Figure S8E:** Scatter plot showing the association of the SNP effects on the apoB level against the SNP effects on CES;

**Supplementary Figure S9A:** Scatter plot showing the association of the SNP effects on LDL-C level against the SNP effects on LAS;

**Supplementary Figure S9B:** Scatter plot showing the association of the SNP effects on HDL-C level against the SNP effects on LAS;

**Supplementary Figure S9C:** Scatter plot showing the association of the SNP effects on TG levels against the SNP effects on LAS;

**Supplementary Figure S9D:** Scatter plot showing the association of the SNP effects on apoA-I level against the SNP effects on LAS;

**Supplementary Figure S9E:** Scatter plot showing the association of the SNP effects on the apoB level against the SNP effects on LAS;

**Supplementary Figure S10A:** Scatter plot showing the association of the SNP effects on LDL-C level against the SNP effects on SVS;

**Supplementary Figure S10B:** Scatter plot showing the association of the SNP effects on HDL-C level against the SNP effects on SVS;

**Supplementary Figure S10C:** Scatter plot showing the association of the SNP effects on TG levels against the SNP effects on SVS;

**Supplementary Figure S10D:** Scatter plot showing the association of the SNP effects on apoA-I level against the SNP effects on SVS;

**Supplementary Figure S10E:** Scatter plot showing the association of the SNP effects on the apoB level against the SNP effects on SVS;

**Supplementary Figure S11A:** Funnel plot showing the association of the SNP effects on LDL-C level against the SNP effects on AS;

**Supplementary Figure S11B:** Funnel plot showing the association of the SNP effects on HDL-C level against the SNP effects on AS;

**Supplementary Figure S11C:** Funnel plot showing the association of the SNP effects on TG levels against the SNP effects on AS;

**Supplementary Figure S11D:** Funnel plot showing the association of the SNP effects on apoA-I level against the SNP effects on AS;

**Supplementary Figure S11E:** Funnel plot showing the association of the SNP effects on the apoB level against the SNP effects on AS;

**Supplementary Figure S12A:** Funnel plot showing the association of the SNP effects on LDL-C level against the SNP effects on AIS;

**Supplementary Figure S12B:** Funnel plot showing the association of the SNP effects on HDL-C level against the SNP effects on AIS;

**Supplementary Figure S12C:** Funnel plot showing the association of the SNP effects on TG levels against the SNP effects on AIS;

**Supplementary Figure S12D:** Funnel plot showing the association of the SNP effects on apoA-I level against the SNP effects on AIS;

**Supplementary Figure S12E:** Funnel plot showing the association of the SNP effects on the apoB level against the SNP effects on AIS;

**Supplementary Figure S13A:** Funnel plot showing the association of the SNP effects on LDL-C level against the SNP effects on CES;

**Supplementary Figure S13B:** Funnel plot showing the association of the SNP effects on HDL-C level against the SNP effects on CES;

**Supplementary Figure S13C:** Funnel plot showing the association of the SNP effects on TG levels against the SNP effects on CES;

**Supplementary Figure S13D:** Funnel plot showing the association of the SNP effects on apoA-I level against the SNP effects on CES;

**Supplementary Figure S13E:** Funnel plot showing the association of the SNP effects on the apoB level against the SNP effects on CES;

**Supplementary Figure S14A:** Funnel plot showing the association of the SNP effects on LDL-C level against the SNP effects on LAS;

**Supplementary Figure S14B:** Funnel plot showing the association of the SNP effects on HDL-C level against the SNP effects on LAS;

**Supplementary Figure S14C:** Funnel plot showing the association of the SNP effects on TG levels against the SNP effects on LAS;

**Supplementary Figure S14D:** Funnel plot showing the association of the SNP effects on apoA-I level against the SNP effects on LAS;

**Supplementary Figure S14E:** Funnel plot showing the association of the SNP effects on the apoB level against the SNP effects on LAS;

**Supplementary Figure S15A:** Funnel plot showing the association of the SNP effects on LDL-C level against the SNP effects on SVS;

**Supplementary Figure S15B:** Funnel plot showing the association of the SNP effects on HDL-C level against the SNP effects on SVS;

**Supplementary Figure S15C:** Funnel plot showing the association of the SNP effects on TG levels against the SNP effects on SVS;

**Supplementary Figure S15D:** Funnel plot showing the association of the SNP effects on apoA-I level against the SNP effects on SVS;

**Supplementary Figure S15E:** Funnel plot showing the association of the SNP effects on the apoB level against the SNP effects on SVS.

Supplementary Table S1: Information of genetic instrumental variants associated with lipid-related traits via uvMR.

| **Exposure** | **IVs** | **Beta** | **SE** | ***p*** | **Effect allele** | **Other allele** | **Eaf** | **mean F-statistic** |
| --- | --- | --- | --- | --- | --- | --- | --- | --- |
| **LDL-C** | rs115458560 | -0.050 | 0.008 | 6.4E-11 | C | T | 0.019 | 42.685 |
|  | rs6667939 | 0.015 | 0.002 | 5E-11 | T | C | 0.719 | 43.165 |
|  | rs12078100 | 0.013 | 0.002 | 0.000000001 | G | C | 0.623 | 37.240 |
|  | rs114165349 | 0.056 | 0.007 | 6.2E-16 | C | G | 0.023 | 65.358 |
|  | rs140584594 | -0.014 | 0.002 | 7.7E-10 | G | A | 0.730 | 37.838 |
|  | rs1183851 | 0.024 | 0.002 | 4.5E-30 | C | T | 0.396 | 129.827 |
|  | rs6680227 | -0.075 | 0.006 | 4.3E-40 | A | G | 0.035 | 175.647 |
|  | rs2642438 | 0.025 | 0.002 | 8E-29 | G | A | 0.703 | 124.097 |
|  | rs28631087 | -0.016 | 0.003 | 2E-10 | C | T | 0.213 | 40.440 |
|  | rs55637835 | -0.019 | 0.003 | 8.4E-09 | T | C | 0.121 | 33.186 |
|  | rs11591147 | -0.348 | 0.008 | 1E-200 | T | G | 0.017 | 1930.426 |
|  | rs472495 | 0.043 | 0.002 | 7.3E-85 | T | G | 0.649 | 381.076 |
|  | rs4970834 | -0.105 | 0.003 | 1E-200 | T | C | 0.187 | 1541.814 |
|  | rs556107 | 0.035 | 0.002 | 1.2E-63 | T | C | 0.523 | 283.610 |
|  | rs34596921 | 0.027 | 0.002 | 2.2E-36 | T | TA | 0.580 | 158.715 |
|  | rs1556562 | 0.019 | 0.002 | 2.1E-14 | T | G | 0.790 | 58.423 |
|  | rs10910476 | 0.012 | 0.002 | 3.7E-09 | T | C | 0.555 | 34.767 |
|  | rs13020929 | 0.015 | 0.002 | 3.4E-12 | A | G | 0.457 | 48.456 |
|  | rs12471768 | 0.014 | 0.002 | 2.5E-09 | C | T | 0.704 | 35.545 |
|  | rs6732741 | -0.028 | 0.003 | 2.9E-18 | A | T | 0.119 | 75.948 |
|  | rs6709904 | -0.043 | 0.003 | 1.1E-39 | G | A | 0.113 | 173.709 |
|  | rs4666384 | -0.017 | 0.002 | 1.1E-13 | G | A | 0.679 | 55.136 |
|  | rs934197 | 0.083 | 0.002 | 1E-200 | A | G | 0.335 | 1427.542 |
|  | rs7562734 | -0.020 | 0.002 | 1.8E-19 | C | G | 0.322 | 81.433 |
|  | rs150474434 | -0.035 | 0.003 | 1.3E-23 | A | G | 0.101 | 100.323 |
|  | rs17050272 | -0.021 | 0.002 | 2.5E-22 | A | G | 0.409 | 94.452 |
|  | rs7569317 | 0.018 | 0.002 | 8.9E-18 | C | T | 0.531 | 73.738 |
|  | rs56236159 | 0.018 | 0.003 | 8.8E-09 | G | T | 0.131 | 33.082 |
|  | rs4954192 | 0.015 | 0.002 | 6.7E-12 | T | C | 0.373 | 47.100 |
|  | rs2287622 | -0.021 | 0.002 | 2.9E-23 | G | A | 0.603 | 98.728 |
|  | rs1250258 | 0.014 | 0.002 | 0.000000006 | T | C | 0.737 | 33.845 |
|  | rs11568318 | 0.026 | 0.004 | 7.8E-10 | A | C | 0.066 | 37.815 |
|  | rs6544713 | -0.054 | 0.002 | 5E-129 | C | T | 0.677 | 584.006 |
|  | rs148150904 | 0.016 | 0.003 | 2.5E-09 | TTAAAG | T | 0.190 | 35.543 |
|  | rs13076933 | -0.021 | 0.002 | 1.6E-18 | G | T | 0.259 | 77.143 |
|  | rs9832727 | -0.015 | 0.002 | 2.1E-11 | G | C | 0.340 | 44.856 |
|  | rs9834932 | -0.032 | 0.004 | 1.5E-18 | G | A | 0.089 | 77.312 |
|  | rs71311871 | -0.028 | 0.004 | 7.5E-14 | G | A | 0.083 | 55.945 |
|  | rs5849920 | -0.014 | 0.002 | 3.1E-10 | G | GT | 0.364 | 39.636 |
|  | rs9289196 | 0.017 | 0.003 | 3E-10 | C | T | 0.174 | 39.703 |
|  | rs113177823 | -0.041 | 0.005 | 1E-18 | A | G | 0.054 | 78.054 |
|  | rs3732359 | -0.017 | 0.003 | 5.7E-12 | A | G | 0.780 | 47.418 |
|  | rs13121616 | 0.013 | 0.002 | 0.000000013 | G | A | 0.694 | 32.294 |
|  | rs9884390 | 0.025 | 0.002 | 5.9E-24 | C | T | 0.234 | 101.876 |
|  | rs13108218 | -0.018 | 0.002 | 1.8E-16 | G | A | 0.615 | 67.824 |
|  | rs7707394 | 0.040 | 0.002 | 2.4E-77 | A | G | 0.357 | 346.538 |
|  | rs12916 | 0.062 | 0.002 | 1.7E-187 | C | T | 0.401 | 852.850 |
|  | rs146433259 | -0.057 | 0.010 | 0.000000041 | T | C | 0.012 | 30.107 |
|  | rs28590710 | -0.018 | 0.003 | 1.4E-10 | A | G | 0.197 | 41.116 |
|  | rs7734476 | 0.019 | 0.002 | 3.3E-19 | A | G | 0.550 | 80.257 |
|  | rs6874202 | 0.032 | 0.002 | 1.6E-50 | C | T | 0.634 | 223.438 |
|  | rs116734477 | -0.047 | 0.005 | 3.2E-19 | T | C | 0.041 | 80.319 |
|  | rs1016988 | -0.017 | 0.003 | 4.8E-11 | C | T | 0.191 | 43.271 |
|  | rs3104412 | -0.019 | 0.002 | 1.8E-19 | G | A | 0.452 | 81.500 |
|  | rs2745353 | 0.013 | 0.002 | 1.1E-09 | T | C | 0.518 | 37.139 |
|  | rs7776054 | -0.016 | 0.002 | 8.2E-12 | G | A | 0.261 | 46.711 |
|  | rs12208357 | 0.057 | 0.004 | 5.4E-44 | T | C | 0.070 | 193.533 |
|  | rs146534110 | 0.068 | 0.009 | 7.8E-14 | T | G | 0.013 | 55.868 |
|  | rs118039278 | 0.084 | 0.004 | 1.8E-102 | A | G | 0.079 | 461.929 |
|  | rs7746081 | -0.023 | 0.002 | 4E-25 | A | G | 0.304 | 107.204 |
|  | rs9496567 | -0.017 | 0.002 | 7.8E-13 | A | G | 0.243 | 51.344 |
|  | rs3823376 | 0.017 | 0.002 | 1E-16 | T | C | 0.502 | 68.917 |
|  | rs34568880 | 0.056 | 0.009 | 6.8E-10 | T | C | 0.013 | 38.082 |
|  | rs35919498 | 0.046 | 0.003 | 1.2E-44 | T | C | 0.117 | 196.577 |
|  | rs3127580 | 0.036 | 0.003 | 2.2E-35 | T | C | 0.155 | 154.141 |
|  | rs3822855 | 0.018 | 0.002 | 4.5E-17 | T | G | 0.402 | 70.530 |
|  | rs117733303 | 0.084 | 0.008 | 2.4E-27 | G | A | 0.018 | 117.318 |
|  | rs2820226 | -0.013 | 0.002 | 8.6E-10 | A | G | 0.452 | 37.621 |
|  | rs9471968 | -0.012 | 0.002 | 0.000000029 | G | A | 0.546 | 30.782 |
|  | rs869412 | -0.014 | 0.003 | 0.000000013 | C | T | 0.226 | 32.381 |
|  | rs28406917 | 0.012 | 0.002 | 0.000000018 | T | C | 0.428 | 31.708 |
|  | rs56130071 | 0.033 | 0.003 | 4.7E-39 | C | G | 0.217 | 170.914 |
|  | rs61003864 | 0.016 | 0.003 | 5.6E-09 | C | T | 0.187 | 33.965 |
|  | rs60612724 | 0.032 | 0.005 | 2.3E-09 | G | A | 0.039 | 35.714 |
|  | rs111338114 | -0.029 | 0.005 | 5.1E-09 | G | A | 0.056 | 34.165 |
|  | rs10231941 | 0.020 | 0.003 | 3.3E-13 | C | T | 0.178 | 53.016 |
|  | rs4148826 | -0.016 | 0.003 | 6.7E-09 | C | T | 0.180 | 33.625 |
|  | rs35990695 | 0.014 | 0.002 | 4.1E-11 | C | CA | 0.477 | 43.562 |
|  | rs2073547 | 0.036 | 0.003 | 2.3E-40 | G | A | 0.184 | 176.896 |
|  | rs117139027 | -0.057 | 0.008 | 5.9E-13 | A | G | 0.018 | 51.867 |
|  | rs28615248 | 0.018 | 0.003 | 2.7E-12 | C | T | 0.196 | 48.920 |
|  | rs4738684 | -0.032 | 0.002 | 3E-46 | G | A | 0.665 | 203.842 |
|  | rs34207171 | -0.014 | 0.002 | 0.000000026 | C | CA | 0.701 | 30.980 |
|  | rs55831924 | 0.016 | 0.002 | 6.9E-13 | T | C | 0.361 | 51.571 |
|  | rs1350559 | 0.014 | 0.002 | 1.1E-10 | G | C | 0.401 | 41.601 |
|  | rs2737265 | -0.020 | 0.002 | 2.5E-18 | G | A | 0.280 | 76.237 |
|  | rs28601761 | -0.062 | 0.002 | 2.9E-185 | G | C | 0.419 | 842.617 |
|  | rs9987289 | 0.045 | 0.004 | 5.1E-36 | G | A | 0.909 | 157.019 |
|  | rs34265667 | -0.032 | 0.006 | 0.000000026 | A | G | 0.034 | 30.995 |
|  | rs28814720 | 0.012 | 0.002 | 0.000000015 | G | A | 0.520 | 32.044 |
|  | rs10448340 | -0.015 | 0.002 | 1.7E-11 | G | T | 0.320 | 45.304 |
|  | rs3780181 | -0.028 | 0.004 | 2.6E-11 | G | A | 0.068 | 44.477 |
|  | rs6475606 | -0.020 | 0.002 | 2.3E-22 | T | C | 0.484 | 94.624 |
|  | rs6560499 | -0.012 | 0.002 | 8.9E-09 | A | G | 0.576 | 33.073 |
|  | rs2066714 | 0.021 | 0.003 | 1.4E-11 | C | T | 0.129 | 45.671 |
|  | rs2740488 | -0.025 | 0.002 | 1.3E-26 | C | A | 0.265 | 113.942 |
|  | rs79828839 | 0.014 | 0.003 | 0.000000029 | T | C | 0.199 | 30.741 |
|  | rs2068888 | -0.019 | 0.002 | 4.6E-20 | A | G | 0.451 | 84.126 |
|  | rs76468627 | -0.022 | 0.004 | 0.000000037 | T | C | 0.076 | 30.288 |
|  | rs2250802 | -0.018 | 0.002 | 6.1E-15 | A | G | 0.724 | 60.856 |
|  | rs17476364 | -0.022 | 0.003 | 1.2E-10 | C | T | 0.108 | 41.502 |
|  | rs12246352 | 0.026 | 0.003 | 6E-14 | G | A | 0.104 | 56.356 |
|  | rs11601507 | 0.032 | 0.004 | 1.8E-15 | A | C | 0.069 | 63.223 |
|  | rs11226108 | -0.016 | 0.003 | 1.6E-09 | C | G | 0.192 | 36.469 |
|  | rs10832963 | 0.017 | 0.002 | 5.1E-13 | G | T | 0.744 | 52.162 |
|  | rs174564 | -0.032 | 0.002 | 4.5E-48 | G | A | 0.349 | 212.221 |
|  | rs964184 | -0.058 | 0.003 | 8.2E-79 | C | G | 0.866 | 353.285 |
|  | rs7108486 | -0.039 | 0.007 | 0.000000024 | C | T | 0.024 | 31.117 |
|  | rs4930163 | 0.017 | 0.003 | 9.2E-10 | A | G | 0.159 | 37.477 |
|  | rs4307732 | 0.045 | 0.003 | 9.8E-40 | A | G | 0.106 | 174.017 |
|  | rs78447482 | -0.013 | 0.002 | 2.1E-09 | TACTC | T | 0.588 | 35.874 |
|  | rs2160994 | 0.018 | 0.002 | 6.3E-17 | C | T | 0.647 | 69.878 |
|  | rs78508096 | 0.018 | 0.002 | 1.3E-12 | A | G | 0.226 | 50.324 |
|  | rs61754230 | 0.043 | 0.007 | 9.1E-09 | T | C | 0.020 | 33.026 |
|  | rs35882350 | 0.014 | 0.002 | 0.000000004 | G | A | 0.261 | 34.615 |
|  | rs11065385 | -0.024 | 0.002 | 2E-27 | G | A | 0.691 | 117.695 |
|  | rs2391825 | -0.013 | 0.002 | 0.000000026 | A | G | 0.279 | 30.962 |
|  | rs2238162 | -0.017 | 0.002 | 2.8E-15 | T | C | 0.523 | 62.424 |
|  | rs6602912 | 0.022 | 0.002 | 7.2E-22 | G | T | 0.285 | 92.356 |
|  | rs11621792 | 0.019 | 0.002 | 1.1E-19 | T | C | 0.453 | 82.335 |
|  | rs60229127 | -0.013 | 0.002 | 2.4E-10 | G | GA | 0.557 | 40.083 |
|  | rs61988556 | -0.022 | 0.004 | 1.7E-09 | C | T | 0.086 | 36.288 |
|  | rs145730801 | 0.036 | 0.005 | 3.3E-12 | C | T | 0.044 | 48.501 |
|  | rs35980001 | -0.022 | 0.003 | 3.8E-18 | G | GC | 0.787 | 75.429 |
|  | rs2043085 | -0.017 | 0.002 | 1.9E-15 | C | T | 0.612 | 63.135 |
|  | rs12445804 | 0.023 | 0.004 | 9.5E-09 | A | G | 0.075 | 32.945 |
|  | rs7202323 | -0.026 | 0.002 | 6.5E-25 | G | T | 0.230 | 106.257 |
|  | rs4782568 | -0.017 | 0.002 | 4.9E-15 | G | C | 0.451 | 61.308 |
|  | rs183130 | -0.033 | 0.002 | 2.1E-49 | T | C | 0.324 | 218.312 |
|  | rs9929977 | 0.017 | 0.002 | 1E-14 | A | T | 0.370 | 59.897 |
|  | rs34042070 | 0.049 | 0.003 | 2.9E-73 | G | C | 0.188 | 327.782 |
|  | rs72631343 | -0.029 | 0.003 | 4.6E-21 | G | C | 0.129 | 88.676 |
|  | rs2611867 | -0.027 | 0.002 | 4.7E-37 | G | A | 0.514 | 161.751 |
|  | rs1801689 | 0.062 | 0.006 | 1.8E-24 | C | A | 0.031 | 104.281 |
|  | rs72911393 | -0.018 | 0.003 | 5.3E-10 | T | C | 0.148 | 38.552 |
|  | rs55714927 | -0.026 | 0.003 | 2.4E-23 | T | C | 0.190 | 99.071 |
|  | rs9894946 | -0.017 | 0.003 | 2.3E-09 | G | A | 0.841 | 35.692 |
|  | rs35278712 | 0.018 | 0.002 | 5.3E-15 | GAAAT | G | 0.712 | 61.141 |
|  | rs77542162 | 0.128 | 0.007 | 2.1E-74 | G | A | 0.022 | 333.074 |
|  | rs77960347 | 0.071 | 0.009 | 7.7E-15 | G | A | 0.013 | 60.403 |
|  | rs7241918 | 0.016 | 0.003 | 0.00000001 | T | G | 0.824 | 32.804 |
|  | rs440677 | -0.016 | 0.002 | 1.5E-13 | A | G | 0.623 | 54.634 |
|  | rs35511051 | -0.022 | 0.003 | 1.1E-17 | A | C | 0.210 | 73.254 |
|  | rs2738447 | 0.042 | 0.002 | 2.9E-89 | C | A | 0.593 | 401.301 |
|  | rs1551891 | -0.174 | 0.004 | 1E-200 | A | G | 0.088 | 2250.135 |
|  | rs4263041 | -0.070 | 0.003 | 4.9E-171 | G | A | 0.283 | 777.197 |
|  | rs111273322 | 0.022 | 0.003 | 8E-11 | A | G | 0.108 | 42.248 |
|  | rs5112 | 0.028 | 0.002 | 9.4E-36 | G | C | 0.533 | 155.790 |
|  | rs143020224 | -0.169 | 0.003 | 1E-200 | G | C | 0.119 | 2773.120 |
|  | rs8107974 | -0.105 | 0.004 | 2.7E-158 | T | A | 0.076 | 718.564 |
|  | rs200046586 | 0.309 | 0.008 | 1E-200 | C | CA | 0.981 | 1560.116 |
|  | rs375972689 | 0.168 | 0.010 | 1.5E-61 | G | T | 0.011 | 274.001 |
|  | rs1010759 | -0.023 | 0.003 | 1.5E-14 | A | G | 0.140 | 59.124 |
|  | rs1883711 | 0.103 | 0.006 | 7.5E-64 | C | G | 0.031 | 284.595 |
|  | rs6031587 | -0.026 | 0.004 | 5.7E-10 | T | C | 0.072 | 38.424 |
|  | rs6074012 | 0.012 | 0.002 | 0.000000024 | C | T | 0.524 | 31.102 |
|  | rs17569873 | 0.017 | 0.003 | 4.8E-11 | T | C | 0.201 | 43.244 |
|  | rs438568 | 0.012 | 0.002 | 5.5E-09 | G | A | 0.609 | 34.002 |
|  | rs2618566 | -0.025 | 0.002 | 9.6E-30 | T | G | 0.660 | 128.313 |
|  | rs200571020 | -0.026 | 0.002 | 1.8E-34 | C | CTTTTTTT | 0.541 | 149.972 |
|  | rs2256814 | 0.015 | 0.003 | 6.1E-09 | A | G | 0.198 | 33.800 |
|  | rs5843957 | 0.014 | 0.002 | 8.1E-11 | TG | T | 0.571 | 42.229 |
|  | rs960596 | 0.014 | 0.002 | 1.1E-09 | T | C | 0.339 | 37.098 |
|  | rs12162782 | 0.013 | 0.002 | 3.5E-09 | G | T | 0.344 | 34.904 |
| **HDL-C** | rs114165349 | -0.081 | 0.006 | 3.9E-37 | C | G | 0.023 | 162.120 |
|  | rs12045101 | -0.015 | 0.002 | 6.7E-11 | T | C | 0.241 | 42.617 |
|  | rs2642438 | 0.028 | 0.002 | 2.1E-40 | G | A | 0.703 | 177.039 |
|  | rs76962725 | -0.028 | 0.005 | 0.000000046 | A | G | 0.037 | 29.860 |
|  | rs185073199 | 0.064 | 0.010 | 2.4E-10 | A | T | 0.010 | 40.074 |
|  | rs75246752 | 0.049 | 0.008 | 5.4E-09 | C | G | 0.013 | 34.055 |
|  | rs1771582 | 0.013 | 0.002 | 6E-11 | G | T | 0.557 | 42.822 |
|  | rs4650994 | -0.018 | 0.002 | 2.1E-21 | A | G | 0.532 | 90.252 |
|  | rs61805075 | -0.026 | 0.002 | 6E-37 | A | G | 0.329 | 161.246 |
|  | rs141440048 | 0.044 | 0.008 | 7.8E-09 | T | C | 0.018 | 33.327 |
|  | rs2298632 | 0.014 | 0.002 | 9.7E-14 | T | C | 0.498 | 55.435 |
|  | rs771481 | 0.029 | 0.002 | 9.1E-32 | A | T | 0.184 | 137.560 |
|  | rs3747973 | 0.014 | 0.002 | 2.7E-13 | G | A | 0.593 | 53.433 |
|  | rs2298214 | -0.012 | 0.002 | 1.6E-10 | A | C | 0.577 | 40.879 |
|  | rs57760538 | 0.013 | 0.002 | 0.000000036 | G | GT | 0.762 | 30.329 |
|  | rs1168114 | 0.016 | 0.002 | 7.5E-15 | G | A | 0.652 | 60.455 |
|  | rs6693842 | 0.014 | 0.002 | 7.1E-12 | C | T | 0.365 | 46.988 |
|  | rs61748951 | -0.036 | 0.006 | 0.000000017 | A | C | 0.024 | 31.765 |
|  | rs12740811 | -0.018 | 0.003 | 0.000000016 | G | A | 0.094 | 31.950 |
|  | rs2516331 | 0.013 | 0.002 | 1.1E-10 | A | C | 0.376 | 41.596 |
|  | rs2281718 | 0.060 | 0.002 | 1E-200 | T | A | 0.613 | 931.604 |
|  | rs12740374 | 0.029 | 0.002 | 2.2E-36 | T | G | 0.221 | 158.711 |
|  | rs140584594 | 0.031 | 0.002 | 3.1E-48 | G | A | 0.730 | 212.980 |
|  | rs267738 | 0.021 | 0.002 | 9.9E-21 | G | T | 0.219 | 87.184 |
|  | rs12046972 | -0.015 | 0.002 | 1E-14 | C | T | 0.564 | 59.839 |
|  | rs557933 | 0.015 | 0.002 | 1.3E-15 | C | A | 0.520 | 63.871 |
|  | rs676210 | 0.059 | 0.002 | 4.5E-140 | A | G | 0.205 | 634.819 |
|  | rs7583067 | 0.015 | 0.002 | 6.6E-11 | T | C | 0.240 | 42.628 |
|  | rs17713879 | 0.014 | 0.002 | 2E-12 | A | G | 0.365 | 49.470 |
|  | rs13402475 | -0.025 | 0.002 | 1.9E-23 | G | C | 0.815 | 99.536 |
|  | rs2362541 | -0.011 | 0.002 | 0.000000011 | G | T | 0.508 | 32.598 |
|  | rs2364723 | 0.012 | 0.002 | 3.4E-09 | C | G | 0.319 | 34.936 |
|  | rs12475332 | 0.013 | 0.002 | 2.5E-09 | G | T | 0.261 | 35.526 |
|  | rs907866 | -0.018 | 0.002 | 1.4E-21 | A | G | 0.445 | 91.112 |
|  | rs2723065 | 0.015 | 0.002 | 2.4E-14 | G | A | 0.377 | 58.133 |
|  | rs4599108 | 0.014 | 0.002 | 1.2E-12 | T | C | 0.488 | 50.429 |
|  | rs1446585 | 0.017 | 0.002 | 1.1E-14 | G | A | 0.244 | 59.734 |
|  | rs1083470 | 0.012 | 0.002 | 3.4E-09 | A | G | 0.616 | 34.933 |
|  | rs12998038 | 0.013 | 0.002 | 1.6E-09 | T | C | 0.259 | 36.461 |
|  | rs12986742 | -0.011 | 0.002 | 0.00000003 | C | T | 0.476 | 30.699 |
|  | rs6705285 | 0.012 | 0.002 | 1.9E-09 | T | G | 0.609 | 36.041 |
|  | rs2196808 | 0.013 | 0.002 | 2.5E-09 | C | T | 0.732 | 35.561 |
|  | rs112350227 | -0.032 | 0.006 | 0.000000041 | T | C | 0.028 | 30.086 |
|  | rs59037995 | -0.017 | 0.003 | 1.3E-11 | A | AT | 0.829 | 45.837 |
|  | rs59104589 | 0.015 | 0.002 | 3.5E-14 | T | C | 0.358 | 57.418 |
|  | rs17326656 | -0.022 | 0.002 | 1.5E-23 | T | G | 0.239 | 100.034 |
|  | rs11688682 | 0.015 | 0.002 | 2.8E-11 | C | G | 0.271 | 44.333 |
|  | rs72926946 | -0.021 | 0.002 | 3.1E-23 | A | C | 0.296 | 98.586 |
|  | rs1047891 | -0.019 | 0.002 | 2.2E-20 | A | C | 0.316 | 85.593 |
|  | rs78058190 | -0.078 | 0.005 | 1.8E-57 | A | G | 0.050 | 255.344 |
|  | rs11456863 | 0.019 | 0.002 | 2E-21 | A | AT | 0.649 | 90.297 |
|  | rs13097947 | 0.016 | 0.002 | 3.8E-15 | C | T | 0.649 | 61.824 |
|  | rs62246443 | -0.015 | 0.003 | 3.8E-09 | C | T | 0.174 | 34.722 |
|  | rs113851927 | 0.011 | 0.002 | 0.000000043 | G | GT | 0.687 | 30.018 |
|  | rs4916426 | 0.014 | 0.002 | 2E-10 | G | GA | 0.745 | 40.423 |
|  | rs7622114 | 0.012 | 0.002 | 2.5E-09 | A | C | 0.581 | 35.561 |
|  | rs2159607 | -0.024 | 0.002 | 7.2E-23 | T | G | 0.811 | 96.933 |
|  | rs4855582 | 0.011 | 0.002 | 6.5E-09 | T | C | 0.430 | 33.687 |
|  | rs1225053 | -0.015 | 0.002 | 3.9E-12 | C | T | 0.264 | 48.162 |
|  | rs62271373 | -0.041 | 0.004 | 2.5E-23 | A | T | 0.060 | 99.021 |
|  | rs13066793 | 0.022 | 0.003 | 3.3E-11 | G | A | 0.090 | 43.983 |
|  | rs6762415 | -0.011 | 0.002 | 0.000000012 | G | T | 0.535 | 32.429 |
|  | rs77320712 | -0.013 | 0.002 | 0.000000019 | T | G | 0.235 | 31.619 |
|  | rs10513801 | -0.030 | 0.003 | 6.7E-28 | G | T | 0.137 | 119.881 |
|  | rs13087167 | 0.017 | 0.002 | 2.9E-17 | C | G | 0.633 | 71.380 |
|  | rs3732356 | -0.030 | 0.004 | 1E-14 | T | G | 0.934 | 59.850 |
|  | rs9647335 | 0.028 | 0.002 | 2.4E-30 | T | A | 0.192 | 131.070 |
|  | rs76247316 | -0.011 | 0.002 | 2.4E-09 | C | T | 0.480 | 35.604 |
|  | rs2268840 | 0.017 | 0.002 | 2.2E-14 | C | T | 0.228 | 58.330 |
|  | rs880674 | 0.015 | 0.003 | 0.000000044 | C | T | 0.142 | 29.953 |
|  | rs2237035 | 0.014 | 0.002 | 1.2E-12 | T | G | 0.386 | 50.509 |
|  | rs1471251 | -0.019 | 0.002 | 3.6E-23 | T | A | 0.398 | 98.302 |
|  | rs1383732 | -0.015 | 0.003 | 0.000000014 | G | A | 0.150 | 32.250 |
|  | rs10031010 | 0.014 | 0.002 | 0.000000021 | A | G | 0.185 | 31.355 |
|  | rs13137144 | 0.016 | 0.002 | 1.5E-17 | A | G | 0.461 | 72.698 |
|  | rs71603401 | -0.015 | 0.003 | 0.000000032 | G | A | 0.137 | 30.558 |
|  | rs73243877 | -0.025 | 0.003 | 2.5E-23 | G | A | 0.168 | 99.048 |
|  | rs2098918 | 0.012 | 0.002 | 5.8E-10 | T | C | 0.455 | 38.381 |
|  | rs71269068 | 0.016 | 0.002 | 3.7E-15 | C | T | 0.654 | 61.873 |
|  | rs13111599 | 0.013 | 0.002 | 3.5E-09 | G | A | 0.737 | 34.859 |
|  | rs1395221 | -0.011 | 0.002 | 0.00000001 | T | G | 0.398 | 32.802 |
|  | rs1055582 | 0.014 | 0.002 | 2.1E-13 | T | C | 0.505 | 53.935 |
|  | rs62331150 | -0.013 | 0.002 | 0.000000043 | T | G | 0.205 | 30.021 |
|  | rs13144151 | 0.018 | 0.003 | 3.8E-11 | G | A | 0.850 | 43.692 |
|  | rs1349852 | 0.011 | 0.002 | 5.8E-09 | C | A | 0.475 | 33.905 |
|  | rs6824451 | -0.020 | 0.002 | 1E-25 | A | G | 0.464 | 109.941 |
|  | rs7665587 | 0.014 | 0.002 | 4.7E-13 | C | T | 0.422 | 52.331 |
|  | rs4691379 | 0.012 | 0.002 | 6.2E-09 | T | C | 0.319 | 33.758 |
|  | rs10053349 | 0.012 | 0.002 | 7.9E-10 | C | T | 0.390 | 37.784 |
|  | rs2910949 | 0.013 | 0.002 | 2E-11 | G | T | 0.355 | 44.936 |
|  | rs11429307 | 0.031 | 0.002 | 9.7E-37 | G | GT | 0.809 | 160.314 |
|  | rs2307111 | 0.019 | 0.002 | 2.1E-22 | C | T | 0.395 | 94.786 |
|  | rs2963468 | -0.020 | 0.002 | 3.6E-18 | G | A | 0.235 | 75.544 |
|  | rs7725218 | -0.012 | 0.002 | 1.2E-09 | A | G | 0.338 | 36.965 |
|  | rs67016280 | 0.011 | 0.002 | 8.9E-09 | A | AT | 0.548 | 33.073 |
|  | rs34073570 | -0.011 | 0.002 | 0.000000013 | C | CT | 0.601 | 32.304 |
|  | rs1862205 | 0.011 | 0.002 | 5.8E-09 | A | G | 0.405 | 33.906 |
|  | rs454968 | 0.011 | 0.002 | 0.000000032 | C | T | 0.645 | 30.598 |
|  | rs32578 | 0.013 | 0.002 | 1.1E-10 | A | G | 0.309 | 41.624 |
|  | rs286965 | -0.013 | 0.002 | 1.1E-11 | C | T | 0.632 | 46.106 |
|  | rs138354839 | -0.060 | 0.008 | 7.4E-13 | A | C | 0.015 | 51.444 |
|  | rs116006942 | -0.030 | 0.004 | 1.3E-13 | A | G | 0.061 | 54.803 |
|  | rs115912456 | 0.028 | 0.005 | 4.5E-09 | G | A | 0.041 | 34.398 |
|  | rs1045241 | 0.016 | 0.002 | 2.6E-14 | T | C | 0.271 | 57.995 |
|  | rs9327468 | -0.015 | 0.002 | 1.9E-11 | A | C | 0.755 | 45.081 |
|  | rs2339234 | -0.012 | 0.002 | 7.3E-09 | A | G | 0.683 | 33.460 |
|  | rs188502504 | -0.032 | 0.005 | 5.6E-10 | C | T | 0.036 | 38.473 |
|  | rs254562 | -0.011 | 0.002 | 3.4E-09 | G | A | 0.409 | 34.919 |
|  | rs2814982 | -0.028 | 0.003 | 1.6E-18 | T | C | 0.102 | 77.123 |
|  | rs6939861 | -0.014 | 0.002 | 9.7E-11 | A | G | 0.262 | 41.885 |
|  | rs62428831 | 0.018 | 0.003 | 1E-10 | C | T | 0.142 | 41.804 |
|  | rs1281959 | 0.015 | 0.002 | 1.5E-14 | G | C | 0.526 | 59.097 |
|  | rs1240820 | 0.013 | 0.002 | 3.5E-10 | A | G | 0.293 | 39.395 |
|  | rs35224557 | -0.012 | 0.002 | 0.000000002 | GT | G | 0.597 | 36.001 |
|  | rs11381821 | 0.019 | 0.002 | 4.6E-22 | C | CA | 0.531 | 93.260 |
|  | rs635769 | 0.020 | 0.002 | 1.4E-23 | C | T | 0.628 | 100.110 |
|  | rs41272086 | -0.057 | 0.003 | 8.5E-75 | A | G | 0.106 | 334.845 |
|  | rs9465693 | -0.012 | 0.002 | 6.2E-09 | A | C | 0.305 | 33.768 |
|  | rs6934962 | 0.016 | 0.002 | 7.3E-17 | T | C | 0.402 | 69.578 |
|  | rs2750411 | -0.011 | 0.002 | 0.000000014 | G | T | 0.492 | 32.243 |
|  | rs75479205 | 0.014 | 0.002 | 0.000000012 | G | A | 0.189 | 32.539 |
|  | rs28746806 | 0.017 | 0.002 | 3.1E-16 | C | A | 0.334 | 66.725 |
|  | rs147627829 | -0.054 | 0.005 | 2.5E-30 | A | G | 0.044 | 130.952 |
|  | rs12205778 | 0.016 | 0.002 | 8.6E-14 | A | G | 0.259 | 55.672 |
|  | rs998584 | -0.034 | 0.002 | 1.1E-71 | A | C | 0.483 | 320.515 |
|  | rs2800710 | -0.020 | 0.002 | 1.8E-26 | C | T | 0.518 | 113.330 |
|  | rs9347737 | -0.013 | 0.002 | 4.4E-12 | G | A | 0.428 | 47.916 |
|  | rs6460894 | -0.012 | 0.002 | 1.6E-09 | C | T | 0.339 | 36.467 |
|  | rs17138358 | -0.027 | 0.002 | 2.2E-44 | C | G | 0.398 | 195.335 |
|  | rs201441 | -0.011 | 0.002 | 0.000000014 | G | T | 0.581 | 32.151 |
|  | rs10233430 | -0.020 | 0.002 | 2.1E-26 | C | T | 0.427 | 113.027 |
|  | rs55935382 | 0.018 | 0.002 | 4.9E-18 | A | C | 0.325 | 74.902 |
|  | rs73151974 | -0.016 | 0.003 | 3.2E-09 | T | C | 0.144 | 35.072 |
|  | rs1534696 | 0.017 | 0.002 | 2.9E-18 | A | C | 0.541 | 75.954 |
|  | rs2726111 | -0.015 | 0.002 | 2.6E-13 | G | A | 0.667 | 53.491 |
|  | rs2098368 | -0.012 | 0.002 | 1.8E-09 | T | C | 0.546 | 36.234 |
|  | rs35493868 | 0.037 | 0.002 | 3E-55 | G | C | 0.202 | 245.118 |
|  | rs183906992 | 0.029 | 0.005 | 5.5E-10 | C | T | 0.043 | 38.493 |
|  | rs77605964 | 0.017 | 0.002 | 7.5E-14 | A | G | 0.227 | 55.942 |
|  | rs34940374 | -0.017 | 0.002 | 5.3E-12 | A | G | 0.183 | 47.571 |
|  | rs13235365 | 0.026 | 0.002 | 2.1E-33 | T | C | 0.274 | 145.039 |
|  | rs42125 | -0.040 | 0.007 | 0.000000013 | G | A | 0.023 | 32.390 |
|  | rs12705595 | 0.011 | 0.002 | 0.000000025 | A | G | 0.373 | 31.080 |
|  | rs34518086 | -0.031 | 0.002 | 3.8E-56 | C | CT | 0.463 | 249.219 |
|  | rs7794796 | -0.017 | 0.002 | 1.2E-16 | T | C | 0.332 | 68.613 |
|  | rs9987289 | 0.087 | 0.003 | 9.1E-155 | G | A | 0.909 | 702.382 |
|  | rs75609851 | 0.170 | 0.010 | 1.4E-69 | A | G | 0.010 | 310.879 |
|  | rs140064750 | -0.044 | 0.006 | 3.7E-12 | C | T | 0.025 | 48.281 |
|  | rs75662196 | 0.068 | 0.006 | 6.7E-31 | C | G | 0.028 | 133.583 |
|  | rs61596977 | -0.016 | 0.003 | 3.1E-09 | T | C | 0.140 | 35.136 |
|  | rs7817574 | 0.033 | 0.002 | 3.5E-42 | C | T | 0.185 | 185.210 |
|  | rs330089 | 0.020 | 0.003 | 1.4E-10 | C | T | 0.098 | 41.193 |
|  | rs79153732 | -0.094 | 0.007 | 4.2E-38 | T | C | 0.017 | 166.528 |
|  | rs10504477 | -0.015 | 0.002 | 6.1E-15 | C | T | 0.412 | 60.877 |
|  | rs2247355 | 0.021 | 0.002 | 5.1E-17 | T | C | 0.183 | 70.290 |
|  | rs6469605 | 0.032 | 0.002 | 5.8E-61 | T | C | 0.569 | 271.330 |
|  | rs71571682 | 0.018 | 0.002 | 4.8E-15 | T | TAC | 0.235 | 61.360 |
|  | rs343 | 0.134 | 0.003 | 1E-200 | A | C | 0.083 | 1491.286 |
|  | rs308 | 0.127 | 0.007 | 1.1E-79 | G | T | 0.021 | 357.274 |
|  | rs13269725 | -0.026 | 0.004 | 1.9E-13 | G | A | 0.078 | 54.135 |
|  | rs1431659 | 0.013 | 0.002 | 2.3E-09 | G | A | 0.728 | 35.696 |
|  | rs4871603 | 0.036 | 0.002 | 5.2E-73 | T | C | 0.653 | 326.626 |
|  | rs61435086 | 0.089 | 0.009 | 3.7E-25 | C | T | 0.012 | 107.383 |
|  | rs7826177 | 0.011 | 0.002 | 0.000000014 | C | T | 0.635 | 32.252 |
|  | rs72647336 | -0.044 | 0.004 | 2.6E-23 | A | G | 0.057 | 98.946 |
|  | rs4875043 | -0.015 | 0.002 | 1.3E-10 | C | A | 0.217 | 41.357 |
|  | rs10108282 | 0.017 | 0.002 | 6.3E-13 | A | T | 0.206 | 51.752 |
|  | rs75032664 | -0.055 | 0.009 | 1.3E-09 | G | C | 0.013 | 36.765 |
|  | rs142288236 | -0.078 | 0.008 | 1.6E-22 | T | C | 0.015 | 95.363 |
|  | rs80005209 | -0.144 | 0.006 | 2.5E-144 | G | T | 0.030 | 654.390 |
|  | rs4871624 | -0.020 | 0.002 | 3.9E-22 | G | T | 0.287 | 93.593 |
|  | rs2297409 | -0.033 | 0.002 | 1E-43 | A | G | 0.195 | 192.254 |
|  | rs1125873 | 0.016 | 0.002 | 9.6E-17 | T | A | 0.492 | 69.049 |
|  | rs10119644 | 0.013 | 0.002 | 1.8E-12 | A | T | 0.494 | 49.699 |
|  | rs1411432 | -0.014 | 0.002 | 0.000000013 | C | A | 0.186 | 32.389 |
|  | rs74500135 | 0.067 | 0.010 | 2.3E-11 | C | T | 0.010 | 44.732 |
|  | rs1412234 | -0.012 | 0.002 | 3.9E-09 | C | T | 0.327 | 34.680 |
|  | rs7853377 | 0.015 | 0.002 | 4.7E-11 | G | A | 0.216 | 43.297 |
|  | rs2417125 | -0.013 | 0.002 | 5.2E-10 | G | A | 0.284 | 38.614 |
|  | rs532436 | 0.023 | 0.002 | 3.8E-21 | A | G | 0.185 | 89.054 |
|  | rs2520096 | 0.015 | 0.002 | 7.7E-12 | G | A | 0.270 | 46.828 |
|  | rs36036416 | 0.022 | 0.003 | 6.6E-12 | C | T | 0.093 | 47.136 |
|  | rs7036107 | -0.012 | 0.002 | 2.9E-10 | G | A | 0.511 | 39.733 |
|  | rs12686780 | -0.016 | 0.003 | 1.2E-10 | T | C | 0.175 | 41.485 |
|  | rs2740488 | -0.069 | 0.002 | 1E-200 | C | A | 0.265 | 1008.475 |
|  | rs147772065 | 0.029 | 0.005 | 0.000000041 | C | G | 0.035 | 30.105 |
|  | rs686030 | 0.050 | 0.003 | 6.5E-74 | A | C | 0.859 | 330.775 |
|  | rs2066714 | 0.047 | 0.003 | 3.4E-60 | C | T | 0.129 | 267.843 |
|  | rs11254464 | 0.013 | 0.002 | 3.9E-11 | C | T | 0.423 | 43.661 |
|  | rs34045894 | -0.017 | 0.003 | 4.6E-11 | A | G | 0.156 | 43.321 |
|  | rs4917675 | 0.015 | 0.002 | 3.4E-11 | C | T | 0.255 | 43.957 |
|  | rs2804894 | 0.017 | 0.002 | 2.3E-15 | A | G | 0.735 | 62.786 |
|  | rs7924036 | 0.014 | 0.002 | 3.6E-13 | T | G | 0.503 | 52.855 |
|  | rs1970811 | -0.012 | 0.002 | 1.4E-09 | C | T | 0.458 | 36.664 |
|  | rs11009262 | -0.023 | 0.004 | 0.000000012 | T | G | 0.057 | 32.495 |
|  | rs11239536 | 0.029 | 0.002 | 5.5E-38 | A | T | 0.241 | 166.022 |
|  | rs2068888 | 0.019 | 0.002 | 1.3E-23 | A | G | 0.450 | 100.296 |
|  | rs10786114 | 0.024 | 0.003 | 1.2E-16 | T | C | 0.875 | 68.604 |
|  | rs2792751 | -0.036 | 0.002 | 3.2E-64 | C | T | 0.725 | 286.275 |
|  | rs12781812 | 0.011 | 0.002 | 0.000000034 | T | G | 0.401 | 30.445 |
|  | rs12411732 | -0.031 | 0.003 | 6.8E-29 | A | G | 0.146 | 124.437 |
|  | rs61884005 | 0.016 | 0.003 | 0.000000024 | G | C | 0.121 | 31.102 |
|  | rs57512892 | 0.021 | 0.002 | 9.6E-21 | T | TAC | 0.596 | 87.236 |
|  | rs559355 | -0.035 | 0.003 | 9E-41 | T | A | 0.157 | 178.772 |
|  | rs117847213 | 0.030 | 0.005 | 1.2E-09 | G | A | 0.041 | 36.902 |
|  | rs59781045 | 0.074 | 0.004 | 4.5E-85 | T | C | 0.068 | 382.033 |
|  | rs17309930 | -0.022 | 0.002 | 1.5E-20 | A | C | 0.205 | 86.363 |
|  | rs2302263 | -0.036 | 0.003 | 1.6E-27 | T | C | 0.089 | 118.116 |
|  | rs11021232 | -0.017 | 0.002 | 1.7E-11 | C | T | 0.181 | 45.302 |
|  | rs964184 | 0.105 | 0.003 | 1E-200 | C | G | 0.866 | 1423.587 |
|  | rs141469619 | -0.203 | 0.010 | 8.7E-89 | G | A | 0.010 | 399.078 |
|  | rs4614 | -0.018 | 0.002 | 3.8E-20 | G | A | 0.406 | 84.540 |
|  | rs16928809 | -0.026 | 0.003 | 1.7E-15 | A | G | 0.093 | 63.342 |
|  | rs12575456 | 0.045 | 0.002 | 1.4E-106 | A | G | 0.322 | 480.789 |
|  | rs117762989 | -0.027 | 0.005 | 9.3E-09 | T | C | 0.043 | 32.975 |
|  | rs4930352 | 0.016 | 0.002 | 7.3E-17 | T | G | 0.494 | 69.587 |
|  | rs689183 | -0.016 | 0.002 | 7.7E-13 | T | G | 0.751 | 51.351 |
|  | rs117291242 | -0.032 | 0.005 | 4.5E-10 | T | C | 0.037 | 38.872 |
|  | rs79634051 | 0.039 | 0.006 | 9E-12 | C | G | 0.028 | 46.532 |
|  | rs564832 | -0.012 | 0.002 | 0.000000012 | C | T | 0.315 | 32.474 |
|  | rs34389751 | -0.011 | 0.002 | 0.00000001 | GT | G | 0.365 | 32.817 |
|  | rs2155220 | -0.011 | 0.002 | 0.000000038 | T | C | 0.438 | 30.239 |
|  | rs11218738 | 0.023 | 0.002 | 1.4E-26 | A | G | 0.249 | 113.804 |
|  | rs174566 | -0.056 | 0.002 | 1.2E-174 | G | A | 0.350 | 793.848 |
|  | rs10774439 | 0.021 | 0.002 | 1.4E-16 | A | G | 0.815 | 68.240 |
|  | rs11045171 | 0.028 | 0.002 | 4.3E-32 | G | A | 0.198 | 139.046 |
|  | rs7316878 | 0.012 | 0.002 | 0.000000012 | C | T | 0.434 | 32.546 |
|  | rs2645979 | 0.011 | 0.002 | 8.7E-09 | A | G | 0.357 | 33.110 |
|  | rs12229011 | -0.026 | 0.003 | 7.8E-16 | T | C | 0.097 | 64.917 |
|  | rs11614202 | 0.023 | 0.003 | 3E-18 | G | A | 0.842 | 75.909 |
|  | rs7488780 | 0.015 | 0.002 | 8.5E-10 | C | G | 0.204 | 37.636 |
|  | rs200573817 | 0.015 | 0.002 | 4.6E-11 | A | G | 0.755 | 43.346 |
|  | rs58298943 | 0.020 | 0.003 | 3.8E-09 | T | C | 0.084 | 34.729 |
|  | rs61352607 | 0.031 | 0.002 | 4.4E-43 | T | G | 0.241 | 189.376 |
|  | rs3924313 | -0.024 | 0.002 | 1.6E-31 | A | G | 0.322 | 136.431 |
|  | rs58123204 | -0.018 | 0.003 | 2.9E-12 | G | A | 0.153 | 48.732 |
|  | rs202079372 | -0.011 | 0.002 | 0.000000011 | T | C | 0.496 | 32.655 |
|  | rs2111216 | 0.021 | 0.002 | 5E-28 | G | A | 0.594 | 120.463 |
|  | rs71445274 | 0.035 | 0.002 | 2.5E-67 | T | A | 0.336 | 300.553 |
|  | rs921919 | -0.042 | 0.002 | 1.4E-90 | A | G | 0.669 | 407.279 |
|  | rs113740515 | 0.038 | 0.002 | 1.2E-58 | A | G | 0.209 | 260.714 |
|  | rs7305678 | -0.017 | 0.003 | 4.2E-09 | G | T | 0.866 | 34.529 |
|  | rs11171710 | -0.011 | 0.002 | 2.8E-09 | A | G | 0.448 | 35.340 |
|  | rs111363680 | 0.033 | 0.006 | 0.000000031 | T | C | 0.031 | 30.639 |
|  | rs9604045 | 0.018 | 0.002 | 2E-14 | T | G | 0.254 | 58.544 |
|  | rs150861794 | -0.045 | 0.008 | 0.000000004 | T | C | 0.018 | 34.612 |
|  | rs76428106 | -0.062 | 0.009 | 1.2E-12 | C | T | 0.013 | 50.428 |
|  | rs117230571 | -0.027 | 0.004 | 2.2E-13 | G | A | 0.077 | 53.834 |
|  | rs549058 | 0.017 | 0.003 | 5.7E-09 | T | G | 0.120 | 33.938 |
|  | rs367677 | 0.017 | 0.002 | 1.2E-13 | G | A | 0.240 | 54.998 |
|  | rs8014289 | 0.015 | 0.002 | 4.6E-15 | G | A | 0.562 | 61.435 |
|  | rs17124112 | -0.021 | 0.004 | 3.8E-09 | A | C | 0.080 | 34.725 |
|  | rs1760940 | 0.012 | 0.002 | 0.000000039 | C | A | 0.247 | 30.214 |
|  | rs4899251 | -0.023 | 0.004 | 0.000000021 | T | C | 0.942 | 31.418 |
|  | rs8007841 | -0.013 | 0.002 | 1.8E-10 | C | T | 0.657 | 40.695 |
|  | rs7158166 | 0.014 | 0.002 | 5.1E-13 | C | T | 0.594 | 52.180 |
|  | rs1955512 | 0.011 | 0.002 | 0.000000024 | A | G | 0.576 | 31.169 |
|  | rs13379043 | 0.020 | 0.002 | 4.7E-20 | C | T | 0.280 | 84.118 |
|  | rs2498786 | -0.025 | 0.002 | 2.6E-38 | G | C | 0.616 | 167.507 |
|  | rs10162642 | -0.048 | 0.002 | 4.6E-93 | A | G | 0.212 | 418.734 |
|  | rs35980001 | -0.119 | 0.002 | 1E-200 | G | GC | 0.787 | 2572.903 |
|  | rs1270076 | 0.014 | 0.002 | 3.5E-10 | G | A | 0.777 | 39.365 |
|  | rs147460434 | -0.015 | 0.002 | 1.2E-09 | A | AAAAAT | 0.818 | 36.985 |
|  | rs4074448 | 0.015 | 0.002 | 1.3E-14 | A | G | 0.589 | 59.382 |
|  | rs7170463 | 0.019 | 0.002 | 4.2E-20 | G | A | 0.311 | 84.348 |
|  | rs150844304 | -0.091 | 0.006 | 1.4E-51 | C | A | 0.026 | 228.320 |
|  | rs11631178 | 0.021 | 0.003 | 1.3E-11 | C | T | 0.104 | 45.792 |
|  | rs28362901 | -0.024 | 0.003 | 1.2E-12 | A | C | 0.088 | 50.461 |
|  | rs28510484 | -0.015 | 0.003 | 1.2E-09 | C | G | 0.170 | 37.005 |
|  | rs1601934 | -0.101 | 0.002 | 1E-200 | A | G | 0.687 | 2380.869 |
|  | rs3814883 | -0.015 | 0.002 | 5.9E-16 | T | C | 0.482 | 65.458 |
|  | rs4784709 | -0.075 | 0.005 | 3E-55 | A | T | 0.959 | 245.116 |
|  | rs12921195 | -0.017 | 0.003 | 3.2E-09 | A | C | 0.140 | 35.052 |
|  | rs201639483 | 0.011 | 0.002 | 0.000000024 | T | TA | 0.596 | 31.137 |
|  | rs112233856 | -0.053 | 0.006 | 8E-20 | G | A | 0.029 | 83.060 |
|  | rs55781197 | 0.059 | 0.003 | 4E-88 | G | A | 0.115 | 396.027 |
|  | rs12928099 | 0.021 | 0.002 | 3.7E-25 | A | C | 0.296 | 107.361 |
|  | rs113966472 | 0.032 | 0.006 | 4.8E-09 | A | G | 0.032 | 34.259 |
|  | rs7186799 | -0.022 | 0.002 | 5.2E-31 | C | A | 0.437 | 134.102 |
|  | rs71647892 | -0.063 | 0.009 | 5.2E-13 | C | T | 0.012 | 52.129 |
|  | rs75152587 | -0.094 | 0.008 | 1.1E-28 | T | G | 0.013 | 123.432 |
|  | rs9989419 | 0.144 | 0.002 | 1E-200 | G | A | 0.606 | 5569.869 |
|  | rs4330777 | -0.020 | 0.002 | 1.3E-25 | A | G | 0.475 | 109.375 |
|  | rs140164052 | -0.042 | 0.005 | 1.2E-14 | A | G | 0.032 | 59.589 |
|  | rs12926854 | 0.012 | 0.002 | 9.5E-09 | G | A | 0.270 | 32.947 |
|  | rs11640494 | -0.015 | 0.002 | 6.5E-16 | A | G | 0.457 | 65.282 |
|  | rs79600951 | -0.107 | 0.003 | 1E-200 | G | C | 0.092 | 1073.771 |
|  | rs116857878 | 0.048 | 0.007 | 4.5E-12 | T | C | 0.020 | 47.893 |
|  | rs9916613 | -0.013 | 0.002 | 2.3E-11 | A | T | 0.356 | 44.684 |
|  | rs34138141 | -0.018 | 0.002 | 1.6E-16 | T | G | 0.281 | 68.092 |
|  | rs145947882 | -0.165 | 0.006 | 4.7E-161 | C | A | 0.026 | 731.305 |
|  | rs112001035 | -0.047 | 0.004 | 3.3E-30 | A | G | 0.060 | 130.423 |
|  | rs7218647 | 0.011 | 0.002 | 0.000000014 | A | G | 0.559 | 32.147 |
|  | rs141062196 | -0.019 | 0.002 | 5.2E-15 | A | G | 0.194 | 61.166 |
|  | rs4969141 | 0.030 | 0.002 | 3.2E-54 | T | C | 0.490 | 240.392 |
|  | rs3027167 | -0.012 | 0.002 | 1.4E-09 | T | C | 0.682 | 36.710 |
|  | rs9904004 | -0.030 | 0.004 | 4.1E-14 | G | A | 0.061 | 57.107 |
|  | rs2271308 | 0.028 | 0.002 | 3E-38 | C | T | 0.733 | 167.230 |
|  | rs2586116 | -0.016 | 0.002 | 7.1E-14 | G | C | 0.259 | 56.038 |
|  | rs3794752 | 0.013 | 0.002 | 0.000000002 | C | T | 0.281 | 36.000 |
|  | rs8081548 | 0.018 | 0.002 | 6.9E-20 | A | T | 0.659 | 83.345 |
|  | rs35566853 | 0.013 | 0.002 | 0.000000013 | C | CT | 0.783 | 32.278 |
|  | rs56017932 | 0.015 | 0.003 | 8.2E-09 | C | G | 0.200 | 33.228 |
|  | rs73455693 | 0.028 | 0.005 | 0.000000039 | A | G | 0.037 | 30.218 |
|  | rs2435307 | 0.016 | 0.002 | 1.5E-17 | T | C | 0.486 | 72.677 |
|  | rs77960347 | 0.291 | 0.008 | 1E-200 | G | A | 0.013 | 1228.377 |
|  | rs8086351 | 0.084 | 0.003 | 1E-200 | G | C | 0.824 | 1121.740 |
|  | rs983663 | -0.014 | 0.002 | 7.7E-11 | G | A | 0.252 | 42.321 |
|  | rs150237291 | 0.045 | 0.007 | 5.1E-12 | C | T | 0.022 | 47.655 |
|  | rs680321 | 0.011 | 0.002 | 0.000000015 | C | T | 0.457 | 32.058 |
|  | rs2298624 | 0.030 | 0.003 | 1.1E-26 | T | C | 0.133 | 114.323 |
|  | rs4804101 | -0.014 | 0.002 | 5.3E-13 | T | G | 0.439 | 52.087 |
|  | rs3745683 | -0.055 | 0.004 | 3.2E-51 | A | G | 0.075 | 226.624 |
|  | rs429358 | -0.076 | 0.003 | 8.71E-181 | C | T | 0.154 | 822.047 |
|  | rs12462109 | -0.016 | 0.002 | 3.8E-14 | T | C | 0.287 | 57.272 |
|  | rs367070 | 0.042 | 0.002 | 1.3E-74 | G | A | 0.226 | 333.949 |
|  | rs554146 | 0.013 | 0.002 | 1.8E-09 | C | A | 0.705 | 36.192 |
|  | rs62117487 | 0.046 | 0.004 | 1.1E-28 | G | A | 0.057 | 123.453 |
|  | rs116843064 | 0.206 | 0.007 | 3.4E-195 | A | G | 0.019 | 888.329 |
|  | rs62102718 | -0.024 | 0.002 | 3.4E-29 | T | A | 0.286 | 125.815 |
|  | rs4803773 | 0.040 | 0.002 | 8.7E-90 | G | A | 0.499 | 403.677 |
|  | rs7251640 | 0.014 | 0.002 | 5.1E-09 | C | T | 0.194 | 34.154 |
|  | rs2965169 | 0.012 | 0.002 | 6.3E-10 | C | A | 0.389 | 38.220 |
|  | rs144311893 | 0.081 | 0.007 | 1.1E-33 | T | C | 0.022 | 146.385 |
|  | rs1132274 | -0.022 | 0.003 | 1.6E-16 | A | C | 0.154 | 68.079 |
|  | rs6018652 | 0.026 | 0.002 | 4.6E-28 | A | G | 0.793 | 120.642 |
|  | rs6123685 | 0.016 | 0.002 | 3E-13 | A | G | 0.255 | 53.179 |
|  | rs144033177 | -0.056 | 0.008 | 1.4E-12 | C | A | 0.016 | 50.254 |
|  | rs150224153 | -0.093 | 0.006 | 3.1E-59 | T | C | 0.030 | 263.434 |
|  | rs77767539 | 0.045 | 0.008 | 0.000000036 | A | G | 0.014 | 30.368 |
|  | rs6066148 | 0.013 | 0.002 | 0.000000001 | C | G | 0.260 | 37.249 |
|  | rs6059958 | 0.015 | 0.003 | 9.7E-09 | T | C | 0.171 | 32.891 |
|  | rs6142206 | -0.016 | 0.002 | 8.3E-17 | A | G | 0.421 | 69.343 |
|  | rs6073958 | -0.061 | 0.002 | 4.1E-143 | C | T | 0.199 | 648.803 |
|  | rs6075860 | -0.013 | 0.002 | 3.1E-11 | A | G | 0.565 | 44.093 |
|  | rs76602912 | -0.043 | 0.006 | 4E-12 | C | T | 0.024 | 48.115 |
|  | rs407133 | -0.011 | 0.002 | 0.000000046 | C | G | 0.559 | 29.874 |
|  | rs235314 | -0.018 | 0.002 | 2.4E-20 | T | C | 0.532 | 85.400 |
|  | rs7281183 | -0.013 | 0.002 | 6.3E-09 | A | G | 0.737 | 33.747 |
|  | rs2236464 | -0.016 | 0.002 | 3.7E-11 | C | T | 0.212 | 43.753 |
|  | rs3746915 | 0.011 | 0.002 | 0.000000021 | G | A | 0.581 | 31.428 |
|  | rs2256609 | -0.033 | 0.002 | 2.2E-41 | G | A | 0.189 | 181.590 |
|  | rs11704977 | 0.015 | 0.003 | 0.000000043 | T | C | 0.150 | 29.987 |
|  | rs133015 | 0.021 | 0.002 | 1.2E-26 | G | C | 0.440 | 114.090 |
|  | rs9622830 | -0.016 | 0.002 | 2.6E-16 | G | C | 0.354 | 67.117 |
|  | rs460428 | -0.014 | 0.002 | 2.5E-10 | C | T | 0.231 | 40.019 |
| **TG** | rs200610097 | -0.015 | 0.003 | 0.000000024 | C | CA | 0.844 | 31.102 |
|  | rs11206374 | 0.025 | 0.002 | 6.1E-26 | A | G | 0.225 | 110.937 |
|  | rs74090351 | -0.025 | 0.004 | 2.1E-10 | A | G | 0.068 | 40.363 |
|  | rs9436661 | -0.078 | 0.002 | 1E-200 | G | T | 0.353 | 1399.394 |
|  | rs34302257 | -0.012 | 0.002 | 0.000000027 | C | CA | 0.649 | 30.902 |
|  | rs1044808 | -0.025 | 0.004 | 8.3E-12 | C | G | 0.081 | 46.693 |
|  | rs1760801 | -0.020 | 0.002 | 1.5E-20 | A | G | 0.296 | 86.345 |
|  | rs9425589 | -0.014 | 0.002 | 6.2E-12 | A | G | 0.567 | 47.261 |
|  | rs1009590 | 0.021 | 0.004 | 5.7E-09 | C | G | 0.915 | 33.925 |
|  | rs114165349 | 0.082 | 0.007 | 6.3E-35 | C | G | 0.023 | 151.997 |
|  | rs213494 | 0.016 | 0.002 | 5.5E-14 | T | C | 0.648 | 56.556 |
|  | rs698927 | -0.018 | 0.003 | 9.2E-13 | C | A | 0.184 | 51.013 |
|  | rs320369 | -0.013 | 0.002 | 5.2E-09 | G | A | 0.683 | 34.116 |
|  | rs10631642 | 0.013 | 0.002 | 0.000000002 | C | CTTT | 0.689 | 35.945 |
|  | rs36043408 | -0.013 | 0.002 | 1.1E-10 | A | G | 0.502 | 41.606 |
|  | rs61830291 | 0.029 | 0.003 | 1.8E-17 | C | A | 0.096 | 72.380 |
|  | rs11122450 | -0.048 | 0.002 | 1.3E-123 | G | T | 0.612 | 559.145 |
|  | rs2131311 | -0.012 | 0.002 | 0.000000025 | G | A | 0.715 | 31.079 |
|  | rs112108602 | 0.027 | 0.003 | 1.1E-14 | A | AG | 0.910 | 59.769 |
|  | rs1938566 | -0.021 | 0.003 | 2E-15 | T | C | 0.835 | 63.095 |
|  | rs11240358 | 0.014 | 0.002 | 2.3E-11 | A | G | 0.394 | 44.674 |
|  | rs11118310 | 0.019 | 0.002 | 1.1E-21 | T | A | 0.593 | 91.446 |
|  | rs1043897 | -0.015 | 0.002 | 3.4E-13 | T | G | 0.416 | 52.952 |
|  | rs1473886 | -0.018 | 0.002 | 6.9E-20 | T | G | 0.478 | 83.329 |
|  | rs954244 | 0.015 | 0.002 | 1.6E-11 | G | C | 0.255 | 45.396 |
|  | rs2382825 | -0.013 | 0.002 | 4.8E-11 | T | C | 0.623 | 43.256 |
|  | rs676210 | -0.074 | 0.002 | 7.1E-198 | A | G | 0.205 | 900.649 |
|  | rs150419156 | -0.051 | 0.008 | 2.4E-09 | A | G | 0.015 | 35.612 |
|  | rs10210970 | 0.023 | 0.003 | 2E-15 | T | C | 0.131 | 63.089 |
|  | rs3731696 | 0.022 | 0.003 | 5.1E-13 | G | A | 0.121 | 52.150 |
|  | rs78058190 | 0.082 | 0.005 | 1.7E-57 | A | G | 0.050 | 255.394 |
|  | rs12475332 | -0.014 | 0.002 | 5.2E-10 | G | T | 0.261 | 38.587 |
|  | rs3820897 | 0.020 | 0.003 | 3.5E-14 | C | T | 0.820 | 57.432 |
|  | rs72784786 | 0.026 | 0.004 | 2.6E-10 | A | G | 0.061 | 39.974 |
|  | rs6752845 | -0.014 | 0.002 | 6.1E-12 | C | G | 0.431 | 47.296 |
|  | rs7424120 | -0.012 | 0.002 | 1.2E-09 | T | C | 0.602 | 37.025 |
|  | rs1009360 | -0.018 | 0.002 | 3.4E-20 | C | T | 0.419 | 84.741 |
|  | rs5832650 | 0.011 | 0.002 | 0.000000024 | C | CA | 0.593 | 31.124 |
|  | rs1420384 | -0.013 | 0.002 | 1.2E-09 | T | G | 0.667 | 36.906 |
|  | rs11904650 | 0.041 | 0.007 | 0.000000003 | G | A | 0.021 | 35.171 |
|  | rs4128205 | 0.012 | 0.002 | 8.1E-09 | C | A | 0.509 | 33.244 |
|  | rs17326656 | 0.017 | 0.002 | 7.8E-14 | T | G | 0.238 | 55.846 |
|  | rs62135012 | -0.012 | 0.002 | 0.000000015 | A | G | 0.358 | 32.054 |
|  | rs6432622 | -0.011 | 0.002 | 0.000000038 | G | A | 0.490 | 30.230 |
|  | rs78545596 | -0.012 | 0.002 | 0.000000011 | TAA | T | 0.370 | 32.615 |
|  | rs4675812 | -0.014 | 0.002 | 1.5E-12 | A | G | 0.588 | 50.037 |
|  | rs6800707 | 0.030 | 0.003 | 5.3E-32 | G | C | 0.811 | 138.614 |
|  | rs62271373 | 0.042 | 0.004 | 7.8E-23 | A | T | 0.060 | 96.769 |
|  | rs62274099 | 0.012 | 0.002 | 2.3E-09 | T | C | 0.424 | 35.661 |
|  | rs2137557 | 0.012 | 0.002 | 0.000000016 | C | T | 0.646 | 31.978 |
|  | rs6805924 | 0.011 | 0.002 | 0.000000045 | T | G | 0.431 | 29.905 |
|  | rs684773 | 0.029 | 0.002 | 2.5E-35 | C | A | 0.767 | 153.847 |
|  | rs3103310 | 0.020 | 0.002 | 1.1E-17 | G | A | 0.242 | 73.255 |
|  | rs9831084 | -0.012 | 0.002 | 2.9E-09 | C | T | 0.462 | 35.236 |
|  | rs9859117 | 0.015 | 0.002 | 2.4E-09 | C | G | 0.203 | 35.610 |
|  | rs73238173 | -0.017 | 0.003 | 0.000000019 | G | C | 0.130 | 31.549 |
|  | rs10513688 | 0.025 | 0.003 | 1.4E-13 | A | G | 0.097 | 54.753 |
|  | rs79287178 | 0.050 | 0.006 | 7.2E-17 | A | G | 0.031 | 69.607 |
|  | rs11185542 | -0.013 | 0.002 | 0.000000016 | C | G | 0.728 | 31.883 |
|  | rs34389637 | 0.025 | 0.004 | 3.8E-10 | C | CT | 0.935 | 39.201 |
|  | rs6792725 | -0.015 | 0.002 | 6.2E-12 | G | A | 0.692 | 47.263 |
|  | rs55807798 | -0.012 | 0.002 | 6.8E-09 | A | AT | 0.421 | 33.600 |
|  | rs13066793 | -0.022 | 0.003 | 1E-10 | G | A | 0.090 | 41.821 |
|  | rs57996145 | -0.029 | 0.003 | 1.5E-27 | G | GT | 0.833 | 118.315 |
|  | rs12504746 | -0.015 | 0.003 | 1.4E-09 | T | C | 0.193 | 36.662 |
|  | rs2237029 | -0.014 | 0.002 | 8.3E-12 | A | G | 0.601 | 46.685 |
|  | rs13101504 | 0.020 | 0.002 | 4.5E-16 | G | T | 0.413 | 66.006 |
|  | rs1347188 | 0.014 | 0.002 | 1.9E-09 | G | A | 0.246 | 36.099 |
|  | rs71603401 | 0.026 | 0.003 | 1.1E-19 | G | A | 0.137 | 82.496 |
|  | rs11722924 | 0.013 | 0.002 | 8.9E-11 | C | G | 0.535 | 42.044 |
|  | rs13118477 | 0.015 | 0.002 | 1.9E-13 | A | G | 0.393 | 54.073 |
|  | rs148827772 | 0.047 | 0.007 | 5.7E-11 | G | A | 0.023 | 42.912 |
|  | rs11100083 | -0.016 | 0.002 | 1.5E-11 | C | T | 0.226 | 45.493 |
|  | rs2604568 | 0.012 | 0.002 | 0.000000028 | A | T | 0.664 | 30.840 |
|  | rs278981 | 0.013 | 0.002 | 0.000000039 | C | T | 0.758 | 30.197 |
|  | rs3775228 | 0.034 | 0.002 | 5.8E-62 | T | C | 0.400 | 275.915 |
|  | rs6532798 | 0.014 | 0.002 | 1.8E-10 | T | C | 0.697 | 40.638 |
|  | rs13108218 | -0.031 | 0.002 | 9.7E-50 | G | A | 0.615 | 219.859 |
|  | rs7735249 | 0.027 | 0.003 | 1.9E-17 | G | C | 0.113 | 72.270 |
|  | rs55646464 | 0.012 | 0.002 | 0.000000019 | T | G | 0.300 | 31.559 |
|  | rs72644085 | -0.020 | 0.003 | 3.2E-12 | C | T | 0.146 | 48.586 |
|  | rs373798 | 0.032 | 0.004 | 5.3E-17 | ATTTTACAGAGAGCTGATTGGTCTG | A | 0.917 | 70.236 |
|  | rs193735 | 0.033 | 0.005 | 4.9E-10 | A | G | 0.037 | 38.734 |
|  | rs970069 | 0.016 | 0.002 | 2.6E-11 | T | C | 0.212 | 44.455 |
|  | rs6882076 | 0.033 | 0.002 | 4.9E-58 | C | T | 0.634 | 257.891 |
|  | rs62397245 | 0.015 | 0.002 | 3.9E-10 | G | C | 0.222 | 39.151 |
|  | rs11746801 | -0.012 | 0.002 | 2.7E-09 | A | G | 0.637 | 35.365 |
|  | rs4976033 | 0.018 | 0.002 | 4.3E-18 | G | A | 0.402 | 75.186 |
|  | rs7704653 | 0.016 | 0.002 | 2.3E-12 | G | A | 0.723 | 49.203 |
|  | rs13354321 | -0.015 | 0.002 | 1.9E-14 | C | T | 0.410 | 58.683 |
|  | rs1316753 | -0.015 | 0.002 | 7E-13 | C | G | 0.394 | 51.550 |
|  | rs7714361 | 0.014 | 0.002 | 4.4E-09 | C | A | 0.234 | 34.437 |
|  | rs1045241 | -0.021 | 0.002 | 3.2E-20 | T | C | 0.271 | 84.857 |
|  | rs11429307 | -0.047 | 0.003 | 2.7E-75 | G | GT | 0.809 | 337.103 |
|  | rs325485 | -0.012 | 0.002 | 8.6E-09 | G | A | 0.603 | 33.125 |
|  | rs7244 | 0.015 | 0.003 | 6.4E-09 | A | G | 0.174 | 33.697 |
|  | rs78588343 | -0.016 | 0.003 | 2.5E-09 | A | G | 0.176 | 35.550 |
|  | rs28752924 | 0.024 | 0.003 | 1.1E-18 | G | T | 0.243 | 77.870 |
|  | rs186413375 | 0.038 | 0.004 | 2.9E-19 | C | A | 0.083 | 80.518 |
|  | rs138191773 | -0.047 | 0.008 | 2.6E-09 | A | G | 0.017 | 35.485 |
|  | rs9373056 | -0.012 | 0.002 | 7.2E-09 | T | C | 0.320 | 33.472 |
|  | rs17585887 | -0.029 | 0.002 | 1.3E-45 | C | T | 0.591 | 200.933 |
|  | rs9376511 | -0.015 | 0.002 | 3.2E-10 | G | A | 0.203 | 39.532 |
|  | rs4134963 | -0.019 | 0.003 | 9E-14 | T | C | 0.190 | 55.584 |
|  | rs28383314 | 0.038 | 0.002 | 1.5E-76 | C | T | 0.624 | 342.908 |
|  | rs77009508 | 0.045 | 0.004 | 1.8E-32 | G | A | 0.074 | 140.807 |
|  | rs9368503 | -0.012 | 0.002 | 1.8E-09 | T | A | 0.510 | 36.203 |
|  | rs998584 | 0.040 | 0.002 | 2.2E-90 | A | C | 0.483 | 406.426 |
|  | rs62427982 | -0.013 | 0.002 | 4.2E-10 | T | C | 0.322 | 39.007 |
|  | rs186696265 | -0.104 | 0.008 | 2.4E-36 | T | C | 0.015 | 158.530 |
|  | rs729761 | 0.018 | 0.002 | 9.5E-16 | G | T | 0.712 | 64.521 |
|  | rs1835346 | -0.039 | 0.007 | 2.2E-09 | G | A | 0.024 | 35.832 |
|  | rs35786744 | -0.027 | 0.005 | 0.000000019 | A | AT | 0.953 | 31.558 |
|  | rs2983896 | 0.014 | 0.002 | 0.000000012 | A | G | 0.215 | 32.429 |
|  | rs9480889 | 0.016 | 0.002 | 1.3E-11 | G | C | 0.783 | 45.779 |
|  | rs6916318 | 0.027 | 0.002 | 4.2E-41 | T | A | 0.531 | 180.273 |
|  | rs73025562 | 0.014 | 0.002 | 0.000000002 | A | G | 0.246 | 35.926 |
|  | rs852388 | 0.016 | 0.002 | 1.6E-10 | C | G | 0.211 | 40.928 |
|  | rs35140741 | -0.016 | 0.002 | 5E-14 | CAAAAAAAA | C | 0.635 | 56.744 |
|  | rs62459095 | -0.032 | 0.004 | 6.7E-14 | T | C | 0.062 | 56.146 |
|  | rs2240466 | -0.123 | 0.003 | 1E-200 | A | G | 0.123 | 1654.249 |
|  | rs41785 | -0.015 | 0.002 | 8.2E-14 | A | C | 0.417 | 55.749 |
|  | rs62473520 | -0.021 | 0.004 | 0.000000028 | C | T | 0.078 | 30.813 |
|  | rs71538127 | 0.018 | 0.003 | 6.2E-09 | G | C | 0.122 | 33.769 |
|  | rs10242866 | 0.016 | 0.002 | 7E-15 | T | C | 0.399 | 60.599 |
|  | rs10642257 | 0.037 | 0.003 | 1.7E-42 | A | AAGGCC | 0.839 | 186.642 |
|  | rs1799831 | 0.025 | 0.003 | 3.7E-19 | T | C | 0.156 | 80.019 |
|  | rs7786339 | 0.016 | 0.003 | 7.6E-10 | T | C | 0.169 | 37.864 |
|  | rs72555385 | 0.065 | 0.005 | 1.7E-45 | G | A | 0.049 | 200.442 |
|  | rs10277582 | -0.017 | 0.003 | 0.000000017 | T | C | 0.119 | 31.807 |
|  | rs2699805 | -0.020 | 0.002 | 5.1E-23 | A | G | 0.400 | 97.624 |
|  | rs12669911 | -0.012 | 0.002 | 7.9E-09 | C | A | 0.614 | 33.303 |
|  | rs72603744 | -0.013 | 0.002 | 4.6E-09 | C | A | 0.316 | 34.347 |
|  | rs12530679 | -0.012 | 0.002 | 1.8E-09 | G | A | 0.485 | 36.173 |
|  | rs75721796 | 0.021 | 0.002 | 1.2E-17 | A | G | 0.214 | 73.219 |
|  | rs4841580 | -0.025 | 0.002 | 1.4E-34 | C | T | 0.435 | 150.392 |
|  | rs79153732 | 0.077 | 0.008 | 2E-24 | T | C | 0.017 | 104.002 |
|  | rs7000494 | 0.137 | 0.006 | 8E-121 | C | G | 0.030 | 546.317 |
|  | rs150564454 | -0.102 | 0.010 | 4.4E-26 | A | G | 0.012 | 111.600 |
|  | rs3860846 | 0.030 | 0.002 | 4E-40 | T | C | 0.275 | 175.814 |
|  | rs1544980 | 0.024 | 0.002 | 7.6E-22 | C | T | 0.199 | 92.262 |
|  | rs343 | -0.141 | 0.004 | 1E-200 | A | C | 0.083 | 1535.785 |
|  | rs2081687 | -0.026 | 0.002 | 7.6E-36 | C | T | 0.663 | 156.216 |
|  | rs6999569 | -0.086 | 0.002 | 1E-200 | G | A | 0.471 | 1889.263 |
|  | rs13264304 | 0.019 | 0.003 | 8E-12 | G | C | 0.151 | 46.771 |
|  | rs308 | -0.159 | 0.007 | 2.6E-115 | G | T | 0.021 | 520.939 |
|  | rs13269725 | 0.035 | 0.004 | 2E-21 | G | A | 0.078 | 90.374 |
|  | rs3808477 | -0.013 | 0.002 | 1.2E-09 | T | C | 0.279 | 36.904 |
|  | rs11434143 | 0.016 | 0.003 | 6.1E-10 | G | GT | 0.811 | 38.299 |
|  | rs75609851 | -0.199 | 0.010 | 1.6E-87 | A | G | 0.010 | 393.311 |
|  | rs2407278 | -0.034 | 0.006 | 5.4E-09 | G | A | 0.030 | 34.022 |
|  | rs28439112 | 0.013 | 0.002 | 0.000000027 | A | T | 0.256 | 30.884 |
|  | rs2244278 | -0.027 | 0.003 | 1.2E-18 | A | C | 0.121 | 77.736 |
|  | rs76645731 | 0.013 | 0.002 | 3.1E-09 | AG | A | 0.319 | 35.147 |
|  | rs1567353 | 0.015 | 0.002 | 7.4E-12 | G | C | 0.308 | 46.920 |
|  | rs581080 | 0.018 | 0.003 | 1.1E-11 | C | G | 0.819 | 46.078 |
|  | rs696825 | -0.020 | 0.002 | 7.5E-19 | T | C | 0.253 | 78.619 |
|  | rs10797119 | 0.016 | 0.002 | 4.6E-15 | C | T | 0.537 | 61.425 |
|  | rs2131919 | 0.017 | 0.003 | 1E-10 | G | A | 0.164 | 41.793 |
|  | rs7847285 | -0.012 | 0.002 | 0.00000001 | C | T | 0.589 | 32.833 |
|  | rs7861679 | 0.012 | 0.002 | 0.000000018 | T | C | 0.697 | 31.697 |
|  | rs10822163 | -0.032 | 0.002 | 9.7E-59 | G | C | 0.473 | 261.137 |
|  | rs7077812 | 0.014 | 0.003 | 0.000000014 | C | T | 0.195 | 32.234 |
|  | rs2068888 | -0.032 | 0.002 | 3.2E-57 | A | G | 0.451 | 254.197 |
|  | rs56397607 | 0.017 | 0.003 | 1.1E-11 | G | A | 0.183 | 46.070 |
|  | rs80276949 | 0.046 | 0.007 | 8E-12 | A | G | 0.023 | 46.778 |
|  | rs113344423 | 0.043 | 0.004 | 4.1E-24 | A | G | 0.060 | 102.601 |
|  | rs55767272 | -0.028 | 0.004 | 2.8E-12 | C | A | 0.065 | 48.823 |
|  | rs11000468 | -0.015 | 0.002 | 1.5E-10 | T | C | 0.255 | 41.085 |
|  | rs34672664 | 0.022 | 0.004 | 0.000000031 | T | TG | 0.930 | 30.670 |
|  | rs2487294 | 0.018 | 0.002 | 1.6E-16 | T | G | 0.723 | 68.041 |
|  | rs140107293 | -0.023 | 0.003 | 1.6E-16 | G | A | 0.155 | 67.993 |
|  | rs3758413 | 0.011 | 0.002 | 0.000000035 | C | T | 0.418 | 30.392 |
|  | rs10883026 | -0.014 | 0.002 | 5.5E-13 | T | C | 0.522 | 52.030 |
|  | rs1133400 | 0.014 | 0.002 | 9.8E-09 | G | A | 0.220 | 32.871 |
|  | rs499293 | -0.012 | 0.002 | 0.000000017 | A | G | 0.658 | 31.859 |
|  | rs174566 | 0.049 | 0.002 | 2.8E-120 | G | A | 0.350 | 543.834 |
|  | rs480823 | 0.156 | 0.004 | 1E-200 | C | T | 0.079 | 1752.523 |
|  | rs150423652 | 0.286 | 0.012 | 1.4E-119 | T | G | 0.007 | 540.660 |
|  | rs551243 | 0.014 | 0.002 | 4.9E-13 | C | G | 0.469 | 52.234 |
|  | rs10899490 | -0.017 | 0.003 | 2.7E-10 | T | C | 0.161 | 39.874 |
|  | rs61905078 | 0.200 | 0.004 | 1E-200 | C | A | 0.074 | 2774.643 |
|  | rs150555490 | -0.038 | 0.004 | 2.5E-19 | T | C | 0.057 | 80.766 |
|  | rs61729990 | -0.056 | 0.008 | 1.1E-13 | A | C | 0.018 | 55.136 |
|  | rs117291242 | 0.030 | 0.005 | 0.000000017 | T | C | 0.037 | 31.795 |
|  | rs117287238 | -0.039 | 0.006 | 1.4E-10 | A | G | 0.028 | 41.153 |
|  | rs75268115 | -0.021 | 0.004 | 6.8E-09 | G | A | 0.085 | 33.577 |
|  | rs11600815 | -0.032 | 0.005 | 2.1E-12 | A | G | 0.052 | 49.434 |
|  | rs2302263 | 0.044 | 0.003 | 8E-36 | T | C | 0.089 | 156.106 |
|  | rs7947951 | 0.019 | 0.002 | 1.4E-19 | G | A | 0.689 | 81.936 |
|  | rs11030107 | 0.016 | 0.002 | 1.2E-12 | G | A | 0.261 | 50.521 |
|  | rs3974807 | 0.016 | 0.003 | 2.8E-10 | T | C | 0.189 | 39.843 |
|  | rs78484485 | -0.076 | 0.004 | 1.2E-66 | A | G | 0.054 | 297.378 |
|  | rs79357714 | -0.029 | 0.005 | 1.2E-09 | G | A | 0.046 | 36.910 |
|  | rs75942983 | -0.020 | 0.004 | 0.000000025 | T | A | 0.084 | 31.059 |
|  | rs10773049 | -0.029 | 0.002 | 4.1E-46 | C | T | 0.395 | 203.256 |
|  | rs67981690 | 0.030 | 0.003 | 8.3E-24 | G | A | 0.130 | 101.194 |
|  | rs4760254 | -0.028 | 0.002 | 1E-33 | C | G | 0.239 | 146.518 |
|  | rs139386986 | -0.022 | 0.004 | 1.1E-09 | T | C | 0.092 | 37.105 |
|  | rs4761234 | -0.014 | 0.002 | 2E-12 | C | T | 0.484 | 49.474 |
|  | rs10773000 | -0.015 | 0.002 | 1.7E-12 | T | G | 0.332 | 49.836 |
|  | rs7308584 | 0.015 | 0.003 | 5.3E-09 | A | G | 0.184 | 34.086 |
|  | rs7134375 | -0.017 | 0.002 | 1.2E-17 | A | C | 0.431 | 73.106 |
|  | rs7135509 | -0.012 | 0.002 | 0.000000041 | C | T | 0.293 | 30.091 |
|  | rs35763453 | 0.028 | 0.004 | 6.2E-11 | C | T | 0.059 | 42.748 |
|  | rs12424054 | 0.019 | 0.002 | 4.5E-16 | A | G | 0.232 | 66.011 |
|  | rs4765148 | -0.025 | 0.002 | 9.7E-32 | T | G | 0.313 | 137.438 |
|  | rs11274835 | -0.025 | 0.003 | 4.3E-20 | CGAGTGTGGGAATCT | C | 0.174 | 84.295 |
|  | rs149778057 | -0.016 | 0.002 | 1.5E-12 | C | A | 0.328 | 50.110 |
|  | rs7400002 | 0.014 | 0.002 | 3.6E-09 | G | A | 0.231 | 34.845 |
|  | rs7140110 | 0.028 | 0.002 | 1.2E-38 | C | T | 0.298 | 169.021 |
|  | rs6562773 | -0.012 | 0.002 | 0.000000002 | G | A | 0.548 | 35.943 |
|  | rs9561643 | 0.017 | 0.002 | 6.5E-15 | C | A | 0.315 | 60.734 |
|  | rs1340819 | -0.012 | 0.002 | 6.3E-09 | C | A | 0.345 | 33.729 |
|  | rs2812208 | -0.048 | 0.007 | 3.5E-12 | C | G | 0.021 | 48.366 |
|  | rs9584870 | -0.012 | 0.002 | 4.3E-09 | C | T | 0.366 | 34.496 |
|  | rs2070341 | 0.011 | 0.002 | 0.000000028 | T | C | 0.603 | 30.867 |
|  | rs2240533 | -0.013 | 0.002 | 1.6E-09 | C | T | 0.310 | 36.423 |
|  | rs61993685 | -0.023 | 0.004 | 3.9E-10 | C | T | 0.076 | 39.176 |
|  | rs6572807 | 0.012 | 0.002 | 0.000000029 | G | A | 0.267 | 30.773 |
|  | rs12880341 | 0.021 | 0.003 | 1.8E-14 | C | T | 0.159 | 58.722 |
|  | rs56902258 | -0.015 | 0.003 | 1.7E-09 | A | T | 0.196 | 36.316 |
|  | rs139974673 | 0.143 | 0.006 | 2.2E-116 | C | T | 0.026 | 525.934 |
|  | rs11637681 | 0.013 | 0.002 | 0.00000002 | G | A | 0.276 | 31.503 |
|  | rs150460588 | 0.033 | 0.005 | 1.6E-11 | C | T | 0.044 | 45.451 |
|  | rs2043085 | -0.031 | 0.002 | 1.9E-51 | C | T | 0.612 | 227.642 |
|  | rs1077835 | 0.047 | 0.002 | 2.2E-86 | G | A | 0.220 | 388.053 |
|  | rs12440800 | 0.016 | 0.002 | 2.2E-12 | T | A | 0.255 | 49.302 |
|  | rs12902047 | -0.013 | 0.002 | 1.7E-09 | C | A | 0.314 | 36.299 |
|  | rs10152471 | -0.014 | 0.002 | 4E-11 | A | G | 0.389 | 43.596 |
|  | rs275184 | -0.017 | 0.003 | 2.9E-10 | G | T | 0.162 | 39.723 |
|  | rs17184382 | -0.022 | 0.002 | 1.1E-27 | C | A | 0.425 | 118.939 |
|  | rs1037117 | 0.017 | 0.002 | 5.7E-14 | A | G | 0.255 | 56.478 |
|  | rs143076454 | 0.040 | 0.007 | 0.000000039 | A | G | 0.019 | 30.183 |
|  | rs12926107 | 0.013 | 0.002 | 2.7E-10 | G | A | 0.455 | 39.864 |
|  | rs2937124 | -0.018 | 0.002 | 8.1E-18 | T | C | 0.363 | 73.921 |
|  | rs12928099 | -0.028 | 0.002 | 2.9E-38 | A | C | 0.296 | 167.268 |
|  | rs12446515 | -0.033 | 0.002 | 3.5E-55 | T | C | 0.323 | 244.835 |
|  | rs34682685 | 0.034 | 0.003 | 5.8E-25 | A | G | 0.104 | 106.488 |
|  | rs4471666 | -0.022 | 0.004 | 0.000000015 | G | T | 0.069 | 32.058 |
|  | rs28577186 | -0.016 | 0.002 | 1.2E-14 | A | G | 0.665 | 59.611 |
|  | rs742036 | -0.014 | 0.002 | 2.5E-12 | A | G | 0.375 | 49.037 |
|  | rs112381903 | 0.023 | 0.004 | 7.5E-09 | T | A | 0.067 | 33.394 |
|  | rs117431393 | 0.031 | 0.005 | 6.5E-09 | G | A | 0.035 | 33.689 |
|  | rs1801689 | -0.066 | 0.006 | 2.4E-30 | C | A | 0.031 | 131.027 |
|  | rs60856912 | 0.025 | 0.003 | 4.8E-20 | T | G | 0.163 | 84.075 |
|  | rs9889402 | 0.012 | 0.002 | 0.000000036 | A | G | 0.728 | 30.358 |
|  | rs4969179 | -0.018 | 0.002 | 2.8E-18 | G | T | 0.604 | 75.993 |
|  | rs12948505 | 0.014 | 0.003 | 0.000000032 | T | C | 0.194 | 30.566 |
|  | rs2304969 | -0.016 | 0.003 | 0.000000015 | T | G | 0.145 | 32.097 |
|  | rs1292065 | -0.014 | 0.002 | 1.9E-10 | G | C | 0.709 | 40.545 |
|  | rs11078597 | 0.019 | 0.003 | 5.7E-14 | C | T | 0.187 | 56.462 |
|  | rs9902027 | -0.015 | 0.002 | 2.1E-10 | T | C | 0.774 | 40.359 |
|  | rs7215055 | 0.039 | 0.004 | 2.3E-21 | G | A | 0.063 | 90.099 |
|  | rs75634664 | 0.036 | 0.004 | 6.4E-17 | G | C | 0.060 | 69.842 |
|  | rs10775406 | 0.021 | 0.002 | 6.6E-19 | G | A | 0.760 | 78.886 |
|  | rs145947882 | 0.137 | 0.006 | 3.4E-103 | C | A | 0.026 | 465.273 |
|  | rs11664106 | -0.013 | 0.002 | 2.4E-09 | T | A | 0.374 | 35.626 |
|  | rs867939 | -0.014 | 0.002 | 1.8E-11 | A | G | 0.576 | 45.130 |
|  | rs2187114 | -0.019 | 0.003 | 0.000000021 | A | G | 0.101 | 31.408 |
|  | rs6506033 | -0.023 | 0.004 | 2.5E-09 | T | C | 0.073 | 35.566 |
|  | rs62102718 | 0.020 | 0.002 | 3.9E-20 | T | A | 0.286 | 84.487 |
|  | rs5112 | 0.068 | 0.002 | 1E-200 | G | C | 0.533 | 1028.752 |
|  | rs117316645 | 0.028 | 0.005 | 0.000000021 | A | G | 0.042 | 31.443 |
|  | rs62112763 | 0.019 | 0.002 | 5.6E-22 | G | C | 0.439 | 92.874 |
|  | rs483808 | -0.014 | 0.002 | 4E-10 | T | C | 0.702 | 39.098 |
|  | rs62117489 | -0.043 | 0.004 | 3.6E-23 | A | C | 0.056 | 98.317 |
|  | rs2305746 | 0.028 | 0.004 | 1.2E-12 | G | A | 0.933 | 50.539 |
|  | rs8102873 | 0.012 | 0.002 | 9.4E-10 | T | C | 0.585 | 37.454 |
|  | rs10405944 | -0.013 | 0.002 | 6.2E-11 | C | T | 0.483 | 42.762 |
|  | rs483082 | 0.086 | 0.002 | 1E-200 | T | G | 0.235 | 1351.901 |
|  | rs116843064 | -0.227 | 0.007 | 1E-200 | A | G | 0.019 | 986.902 |
|  | rs188247550 | -0.134 | 0.009 | 5.9E-49 | T | C | 0.013 | 216.271 |
|  | rs62128802 | -0.016 | 0.003 | 7.4E-10 | T | C | 0.183 | 37.923 |
|  | rs71368855 | 0.026 | 0.003 | 7.8E-17 | T | C | 0.115 | 69.467 |
|  | rs2092203 | 0.014 | 0.002 | 6.8E-12 | T | C | 0.481 | 47.074 |
|  | rs6073958 | 0.056 | 0.002 | 1.6E-110 | C | T | 0.199 | 498.928 |
|  | rs149142833 | 0.017 | 0.003 | 9.1E-10 | T | C | 0.156 | 37.514 |
|  | rs6028716 | -0.013 | 0.002 | 0.00000002 | A | G | 0.258 | 31.541 |
|  | rs55966194 | -0.018 | 0.002 | 7.1E-16 | G | C | 0.282 | 65.099 |
|  | rs151235402 | 0.052 | 0.008 | 1.5E-10 | T | C | 0.016 | 40.982 |
|  | rs7274718 | 0.016 | 0.002 | 3.8E-15 | A | G | 0.599 | 61.777 |
|  | rs394872 | 0.011 | 0.002 | 0.000000024 | T | C | 0.536 | 31.163 |
|  | rs6517522 | -0.013 | 0.002 | 9.4E-11 | C | T | 0.498 | 41.940 |
|  | rs140288 | -0.013 | 0.002 | 4E-11 | A | G | 0.567 | 43.605 |
|  | rs134551 | -0.012 | 0.002 | 0.000000034 | T | C | 0.335 | 30.472 |
|  | rs2267373 | 0.022 | 0.002 | 1.4E-26 | T | C | 0.581 | 113.795 |
|  | rs5755799 | 0.012 | 0.002 | 0.000000003 | G | C | 0.454 | 35.198 |
|  | rs2071887 | 0.016 | 0.002 | 7.3E-15 | A | T | 0.345 | 60.525 |
|  | rs4253750 | 0.018 | 0.002 | 3.2E-13 | C | T | 0.214 | 53.095 |
| **apoA-I** | rs3768321 | -0.042 | 0.002 | 6.2E-66 | T | G | 0.197 | 294.150 |
|  | rs4660586 | 0.013 | 0.002 | 0.000000018 | T | C | 0.739 | 31.684 |
|  | rs12740374 | 0.044 | 0.002 | 1.1E-78 | T | G | 0.221 | 352.708 |
|  | rs12045101 | -0.015 | 0.002 | 8.9E-11 | T | C | 0.241 | 42.053 |
|  | rs10798615 | -0.015 | 0.002 | 6.2E-15 | G | T | 0.533 | 60.842 |
|  | rs199717562 | 0.015 | 0.003 | 6.4E-09 | G | A | 0.195 | 33.694 |
|  | rs57274629 | 0.015 | 0.002 | 3.3E-13 | G | A | 0.358 | 52.995 |
|  | rs698927 | 0.025 | 0.003 | 2.8E-22 | C | A | 0.184 | 94.252 |
|  | rs140584594 | 0.036 | 0.002 | 1.4E-59 | G | A | 0.730 | 264.980 |
|  | rs2642438 | 0.029 | 0.002 | 3.3E-42 | G | A | 0.703 | 185.360 |
|  | rs557933 | 0.019 | 0.002 | 3.2E-22 | C | A | 0.520 | 93.991 |
|  | rs72761606 | -0.016 | 0.003 | 0.000000028 | C | T | 0.131 | 30.840 |
|  | rs2999141 | -0.012 | 0.002 | 0.000000049 | C | A | 0.276 | 29.750 |
|  | rs1168124 | 0.043 | 0.002 | 1.2E-95 | T | C | 0.651 | 430.572 |
|  | rs9426827 | 0.017 | 0.002 | 2.8E-18 | C | T | 0.478 | 76.008 |
|  | rs3747973 | 0.015 | 0.002 | 9.9E-14 | G | A | 0.593 | 55.390 |
|  | rs2298214 | -0.013 | 0.002 | 4.7E-11 | A | C | 0.577 | 43.298 |
|  | rs472629 | -0.018 | 0.002 | 1.3E-18 | A | G | 0.333 | 77.502 |
|  | rs75246752 | 0.050 | 0.009 | 0.00000001 | C | G | 0.013 | 32.822 |
|  | rs1771591 | -0.013 | 0.002 | 4.5E-10 | A | G | 0.487 | 38.901 |
|  | rs61805076 | -0.015 | 0.002 | 1.5E-13 | C | T | 0.333 | 54.605 |
|  | rs12044156 | -0.012 | 0.002 | 1.5E-09 | C | G | 0.564 | 36.525 |
|  | rs2281719 | 0.053 | 0.002 | 1.5E-155 | T | C | 0.612 | 705.910 |
|  | rs10917383 | 0.020 | 0.003 | 5.7E-12 | G | A | 0.875 | 47.430 |
|  | rs34397747 | -0.039 | 0.004 | 2.2E-28 | C | T | 0.084 | 122.085 |
|  | rs267738 | 0.034 | 0.002 | 7.5E-48 | G | T | 0.219 | 211.216 |
|  | rs10752898 | 0.013 | 0.002 | 1.9E-10 | C | T | 0.558 | 40.609 |
|  | rs76962725 | -0.030 | 0.005 | 0.000000013 | A | G | 0.037 | 32.338 |
|  | rs62135193 | -0.011 | 0.002 | 0.000000042 | T | C | 0.541 | 30.055 |
|  | rs1446585 | 0.019 | 0.002 | 9.2E-17 | G | A | 0.244 | 69.132 |
|  | rs72926946 | -0.015 | 0.002 | 6.2E-13 | A | C | 0.296 | 51.776 |
|  | rs2362541 | -0.011 | 0.002 | 9.1E-09 | G | T | 0.508 | 33.016 |
|  | rs17326656 | -0.018 | 0.002 | 3.1E-15 | T | G | 0.239 | 62.233 |
|  | rs6705285 | 0.011 | 0.002 | 0.000000017 | T | G | 0.609 | 31.860 |
|  | rs2943645 | -0.034 | 0.002 | 1.8E-61 | T | C | 0.647 | 273.635 |
|  | rs10170658 | -0.013 | 0.002 | 0.000000017 | T | G | 0.223 | 31.755 |
|  | rs59104589 | 0.017 | 0.002 | 7.6E-17 | T | C | 0.358 | 69.500 |
|  | rs12987470 | 0.012 | 0.002 | 0.000000022 | T | A | 0.295 | 31.308 |
|  | rs1047891 | -0.026 | 0.002 | 3.3E-34 | A | C | 0.316 | 148.711 |
|  | rs13402475 | -0.024 | 0.003 | 1E-20 | G | C | 0.815 | 87.091 |
|  | rs35135293 | -0.022 | 0.002 | 2.7E-28 | T | C | 0.519 | 121.657 |
|  | rs4599108 | 0.014 | 0.002 | 1.4E-12 | T | C | 0.488 | 50.237 |
|  | rs11691486 | 0.014 | 0.002 | 8.3E-10 | C | T | 0.240 | 37.696 |
|  | rs13024140 | -0.012 | 0.002 | 6.3E-09 | C | G | 0.415 | 33.744 |
|  | rs62114506 | 0.014 | 0.002 | 2.7E-10 | C | G | 0.262 | 39.867 |
|  | rs676210 | 0.060 | 0.002 | 8.71E-136 | A | G | 0.205 | 615.101 |
|  | rs113017476 | 0.031 | 0.005 | 0.000000001 | A | G | 0.039 | 37.317 |
|  | rs2540951 | 0.012 | 0.002 | 0.000000002 | G | A | 0.376 | 35.961 |
|  | rs113011842 | -0.013 | 0.002 | 5E-10 | C | T | 0.306 | 38.682 |
|  | rs75265117 | 0.032 | 0.003 | 1.2E-25 | G | C | 0.120 | 109.682 |
|  | rs78058190 | -0.066 | 0.005 | 2.6E-39 | A | G | 0.050 | 172.077 |
|  | rs13326165 | -0.020 | 0.002 | 8E-16 | G | A | 0.795 | 64.864 |
|  | rs1852922 | 0.012 | 0.002 | 6.4E-09 | A | G | 0.690 | 33.717 |
|  | rs73216701 | 0.016 | 0.002 | 9.4E-17 | G | A | 0.430 | 69.083 |
|  | rs13059175 | 0.015 | 0.002 | 3.6E-12 | T | C | 0.300 | 48.324 |
|  | rs9817452 | 0.017 | 0.002 | 5.2E-18 | T | G | 0.388 | 74.820 |
|  | rs4916426 | 0.014 | 0.002 | 1.4E-09 | G | GA | 0.745 | 36.630 |
|  | rs2972166 | 0.015 | 0.002 | 1.1E-11 | A | G | 0.275 | 46.165 |
|  | rs4676609 | 0.015 | 0.002 | 1.6E-09 | T | C | 0.196 | 36.456 |
|  | rs17008972 | 0.019 | 0.003 | 1E-10 | A | G | 0.124 | 41.774 |
|  | rs3732356 | -0.037 | 0.004 | 2.7E-20 | T | G | 0.934 | 85.178 |
|  | rs34642857 | -0.013 | 0.002 | 4.5E-09 | C | T | 0.251 | 34.391 |
|  | rs6807935 | -0.013 | 0.002 | 8.2E-10 | G | A | 0.343 | 37.708 |
|  | rs1086056 | -0.019 | 0.003 | 3.7E-12 | G | T | 0.845 | 48.299 |
|  | rs73052033 | -0.018 | 0.003 | 3.2E-13 | C | T | 0.185 | 53.102 |
|  | rs6772763 | 0.013 | 0.002 | 8.5E-11 | C | T | 0.565 | 42.144 |
|  | rs4441609 | 0.011 | 0.002 | 0.000000044 | C | T | 0.626 | 29.959 |
|  | rs9647335 | 0.032 | 0.002 | 8.6E-37 | T | A | 0.192 | 160.538 |
|  | rs1400362 | 0.013 | 0.002 | 0.000000033 | C | T | 0.741 | 30.504 |
|  | rs10489044 | -0.018 | 0.002 | 1E-13 | G | A | 0.199 | 55.355 |
|  | rs148827772 | -0.057 | 0.007 | 3.7E-16 | G | A | 0.023 | 66.372 |
|  | rs1055582 | 0.016 | 0.002 | 2.4E-15 | T | C | 0.505 | 62.672 |
|  | rs1580083 | 0.021 | 0.002 | 1.1E-22 | T | A | 0.393 | 96.157 |
|  | rs72729623 | -0.015 | 0.003 | 0.000000044 | T | C | 0.143 | 29.957 |
|  | rs13108218 | -0.013 | 0.002 | 5.3E-11 | G | A | 0.615 | 43.055 |
|  | rs6448429 | -0.022 | 0.003 | 6.4E-17 | T | C | 0.166 | 69.857 |
|  | rs34999031 | -0.012 | 0.002 | 0.000000002 | A | AT | 0.426 | 35.946 |
|  | rs976002 | 0.023 | 0.002 | 4.4E-24 | G | A | 0.245 | 102.463 |
|  | rs1395221 | -0.013 | 0.002 | 3.9E-11 | T | G | 0.398 | 43.658 |
|  | rs28824216 | 0.038 | 0.007 | 0.000000021 | T | C | 0.022 | 31.409 |
|  | rs1789896 | 0.015 | 0.002 | 2.6E-14 | A | G | 0.513 | 58.008 |
|  | rs10023962 | 0.018 | 0.003 | 3.8E-12 | G | T | 0.817 | 48.249 |
|  | rs116006942 | -0.028 | 0.004 | 9.4E-12 | A | G | 0.061 | 46.457 |
|  | rs55801554 | 0.014 | 0.002 | 1.7E-09 | A | C | 0.247 | 36.297 |
|  | rs138354839 | -0.067 | 0.009 | 8.9E-15 | A | C | 0.015 | 60.133 |
|  | rs3749748 | 0.022 | 0.002 | 4.3E-22 | T | C | 0.247 | 93.402 |
|  | rs7700617 | -0.013 | 0.002 | 9.9E-11 | A | C | 0.497 | 41.834 |
|  | rs11429307 | 0.022 | 0.003 | 5E-19 | G | GT | 0.809 | 79.410 |
|  | rs2544654 | 0.013 | 0.002 | 3.7E-09 | T | G | 0.715 | 34.759 |
|  | rs1540687 | 0.012 | 0.002 | 0.000000028 | T | A | 0.674 | 30.863 |
|  | rs286965 | -0.016 | 0.002 | 3.2E-15 | C | T | 0.632 | 62.110 |
|  | rs2963468 | -0.017 | 0.002 | 9E-14 | G | A | 0.235 | 55.574 |
|  | rs62369502 | -0.021 | 0.004 | 6.8E-09 | C | T | 0.076 | 33.579 |
|  | rs1862205 | 0.011 | 0.002 | 0.000000032 | A | G | 0.405 | 30.603 |
|  | rs188502504 | -0.029 | 0.005 | 0.00000003 | C | T | 0.036 | 30.690 |
|  | rs254559 | -0.017 | 0.002 | 1.4E-17 | A | C | 0.405 | 72.814 |
|  | rs351862 | 0.018 | 0.003 | 2.6E-09 | T | C | 0.115 | 35.428 |
|  | rs62405458 | -0.015 | 0.003 | 6.5E-09 | T | C | 0.183 | 33.669 |
|  | rs3798233 | 0.018 | 0.002 | 2.3E-19 | C | A | 0.402 | 80.924 |
|  | rs1406982 | -0.014 | 0.002 | 1.4E-11 | A | T | 0.314 | 45.627 |
|  | rs2395158 | -0.034 | 0.003 | 1E-39 | G | A | 0.169 | 173.931 |
|  | rs9471972 | 0.027 | 0.002 | 1.8E-42 | A | G | 0.535 | 186.560 |
|  | rs4052908 | 0.015 | 0.002 | 4.6E-14 | AATT | A | 0.636 | 56.882 |
|  | rs78807370 | 0.016 | 0.003 | 8.6E-09 | A | G | 0.148 | 33.138 |
|  | rs72959041 | -0.036 | 0.005 | 4.2E-15 | A | G | 0.049 | 61.594 |
|  | rs6557267 | -0.012 | 0.002 | 2.8E-09 | T | C | 0.404 | 35.344 |
|  | rs41272086 | -0.037 | 0.003 | 1.4E-31 | A | G | 0.106 | 136.682 |
|  | rs9347737 | -0.011 | 0.002 | 0.000000029 | G | A | 0.428 | 30.751 |
|  | rs59894631 | 0.023 | 0.002 | 1.5E-21 | C | A | 0.667 | 90.882 |
|  | rs75104038 | -0.039 | 0.004 | 1.3E-21 | A | G | 0.061 | 91.202 |
|  | rs11754773 | -0.040 | 0.003 | 2.7E-32 | G | A | 0.093 | 139.989 |
|  | rs10271082 | -0.019 | 0.002 | 1.7E-21 | C | G | 0.408 | 90.622 |
|  | rs76456334 | -0.037 | 0.005 | 7.4E-13 | C | T | 0.040 | 51.442 |
|  | rs55812947 | -0.016 | 0.003 | 0.00000004 | C | T | 0.129 | 30.162 |
|  | rs4725969 | 0.012 | 0.002 | 2.7E-09 | T | C | 0.340 | 35.402 |
|  | rs55710224 | 0.012 | 0.002 | 6.1E-10 | A | G | 0.499 | 38.292 |
|  | rs2726112 | -0.014 | 0.002 | 2E-12 | G | A | 0.454 | 49.465 |
|  | rs34518086 | -0.025 | 0.002 | 2E-35 | C | CT | 0.463 | 154.292 |
|  | rs34767118 | 0.017 | 0.002 | 8.4E-16 | G | A | 0.326 | 64.776 |
|  | rs17883513 | 0.030 | 0.005 | 0.000000027 | G | A | 0.034 | 30.890 |
|  | rs150483923 | 0.020 | 0.002 | 5.3E-17 | A | C | 0.226 | 70.206 |
|  | rs3828960 | -0.015 | 0.002 | 1.7E-09 | G | A | 0.207 | 36.248 |
|  | rs6977416 | -0.016 | 0.002 | 5.7E-14 | A | G | 0.334 | 56.457 |
|  | rs35745599 | 0.028 | 0.002 | 4E-35 | T | C | 0.239 | 152.917 |
|  | rs17138358 | -0.026 | 0.002 | 6.6E-39 | C | G | 0.399 | 170.215 |
|  | rs112928559 | -0.037 | 0.005 | 2.6E-12 | T | C | 0.040 | 48.982 |
|  | rs36024006 | -0.018 | 0.002 | 1.6E-19 | A | G | 0.452 | 81.705 |
|  | rs71303482 | 0.014 | 0.002 | 1.2E-11 | G | A | 0.684 | 45.936 |
|  | rs4875043 | -0.015 | 0.002 | 2.9E-10 | C | A | 0.217 | 39.750 |
|  | rs326 | 0.083 | 0.002 | 1E-200 | G | A | 0.294 | 1483.151 |
|  | rs71517905 | 0.014 | 0.002 | 3.4E-09 | ACAC | A | 0.743 | 34.939 |
|  | rs49675 | 0.021 | 0.003 | 1.3E-10 | A | G | 0.098 | 41.258 |
|  | rs10504477 | -0.012 | 0.002 | 1.2E-09 | C | T | 0.412 | 36.959 |
|  | rs36096231 | -0.021 | 0.004 | 0.00000004 | T | C | 0.072 | 30.129 |
|  | rs61596977 | -0.016 | 0.003 | 0.000000038 | T | C | 0.140 | 30.227 |
|  | rs2247355 | 0.018 | 0.003 | 3E-12 | T | C | 0.183 | 48.717 |
|  | rs6469605 | 0.035 | 0.002 | 1E-69 | T | C | 0.568 | 311.485 |
|  | rs7817574 | 0.036 | 0.003 | 7.4E-47 | C | T | 0.185 | 206.639 |
|  | rs9987289 | 0.085 | 0.003 | 1E-138 | G | A | 0.909 | 628.525 |
|  | rs59347135 | -0.081 | 0.005 | 1.4E-63 | G | C | 0.046 | 283.353 |
|  | rs117853493 | 0.057 | 0.008 | 1.7E-12 | A | G | 0.015 | 49.783 |
|  | rs1125873 | 0.012 | 0.002 | 5.1E-10 | T | A | 0.492 | 38.633 |
|  | rs532436 | 0.030 | 0.003 | 1.7E-33 | A | G | 0.185 | 145.486 |
|  | rs2520096 | 0.014 | 0.002 | 2.3E-10 | G | A | 0.270 | 40.208 |
|  | rs686030 | 0.056 | 0.003 | 4.7E-89 | A | C | 0.859 | 400.307 |
|  | rs7036107 | -0.011 | 0.002 | 0.000000018 | G | A | 0.511 | 31.686 |
|  | rs2066714 | 0.054 | 0.003 | 7.9E-77 | C | T | 0.129 | 344.156 |
|  | rs2740488 | -0.080 | 0.002 | 1E-200 | C | A | 0.266 | 1306.880 |
|  | rs2297409 | -0.037 | 0.002 | 1.5E-49 | A | G | 0.195 | 218.990 |
|  | rs7032795 | 0.015 | 0.002 | 1.3E-13 | C | T | 0.494 | 54.915 |
|  | rs74500135 | 0.074 | 0.010 | 1.1E-12 | C | T | 0.010 | 50.744 |
|  | rs147772065 | 0.031 | 0.005 | 0.000000006 | C | G | 0.035 | 33.832 |
|  | rs2804894 | 0.016 | 0.002 | 4.8E-13 | A | G | 0.735 | 52.288 |
|  | rs7896518 | 0.015 | 0.002 | 1.6E-14 | G | A | 0.428 | 59.007 |
|  | rs1970811 | -0.012 | 0.002 | 0.000000004 | C | T | 0.458 | 34.631 |
|  | rs2803619 | -0.037 | 0.002 | 2.1E-65 | C | G | 0.725 | 291.708 |
|  | rs75406471 | -0.025 | 0.003 | 1.3E-19 | A | G | 0.154 | 82.023 |
|  | rs2068888 | 0.013 | 0.002 | 9.8E-12 | A | G | 0.450 | 46.364 |
|  | rs2419605 | -0.031 | 0.003 | 2.2E-29 | G | A | 0.148 | 126.645 |
|  | rs2245095 | -0.026 | 0.003 | 1.1E-13 | T | C | 0.089 | 55.096 |
|  | rs34045894 | -0.015 | 0.003 | 0.000000048 | A | G | 0.156 | 29.799 |
|  | rs3802548 | 0.034 | 0.002 | 9.4E-50 | A | T | 0.240 | 219.923 |
|  | rs10883451 | 0.022 | 0.002 | 1.8E-29 | C | T | 0.496 | 127.019 |
|  | rs12273363 | -0.020 | 0.002 | 3.9E-16 | C | T | 0.206 | 66.284 |
|  | rs613808 | -0.085 | 0.002 | 1E-200 | G | A | 0.715 | 1514.941 |
|  | rs3740688 | 0.016 | 0.002 | 2.6E-15 | T | G | 0.545 | 62.579 |
|  | rs4930352 | 0.017 | 0.002 | 5.3E-17 | T | G | 0.494 | 70.221 |
|  | rs11021232 | -0.016 | 0.003 | 1.3E-10 | C | T | 0.181 | 41.311 |
|  | rs7952521 | 0.027 | 0.003 | 2.8E-16 | A | G | 0.106 | 66.938 |
|  | rs78296522 | 0.068 | 0.005 | 9E-47 | A | C | 0.045 | 206.252 |
|  | rs117739035 | -0.031 | 0.005 | 6.6E-09 | T | G | 0.036 | 33.642 |
|  | rs1919309 | 0.012 | 0.002 | 2.8E-09 | C | T | 0.509 | 35.309 |
|  | rs174566 | -0.035 | 0.002 | 1E-64 | G | A | 0.350 | 288.606 |
|  | rs559355 | -0.045 | 0.003 | 8.8E-62 | T | A | 0.157 | 275.091 |
|  | rs141368429 | -0.057 | 0.005 | 1.9E-35 | T | C | 0.056 | 154.416 |
|  | rs58473820 | 0.021 | 0.002 | 1.2E-24 | T | C | 0.378 | 104.980 |
|  | rs1661052 | 0.029 | 0.003 | 6.2E-18 | A | G | 0.908 | 74.462 |
|  | rs2302263 | -0.029 | 0.003 | 1.5E-16 | T | C | 0.089 | 68.168 |
|  | rs11226108 | -0.015 | 0.002 | 4.2E-09 | C | G | 0.192 | 34.536 |
|  | rs11216920 | 0.025 | 0.004 | 1.6E-12 | G | A | 0.084 | 49.972 |
|  | rs7305678 | -0.022 | 0.003 | 5.4E-14 | G | T | 0.866 | 56.585 |
|  | rs11045172 | 0.022 | 0.002 | 2.7E-19 | C | A | 0.198 | 80.658 |
|  | rs4762756 | 0.014 | 0.002 | 4.6E-10 | C | T | 0.736 | 38.826 |
|  | rs10876447 | -0.019 | 0.003 | 9.3E-13 | A | G | 0.154 | 50.981 |
|  | rs111694265 | -0.049 | 0.003 | 6.5E-51 | C | CAAAACAA | 0.901 | 225.228 |
|  | rs11057390 | 0.019 | 0.002 | 1.2E-18 | G | T | 0.295 | 77.637 |
|  | rs7136506 | -0.032 | 0.002 | 2.1E-39 | C | T | 0.216 | 172.511 |
|  | rs11067231 | 0.022 | 0.002 | 3.6E-29 | A | C | 0.524 | 125.693 |
|  | rs851132 | -0.025 | 0.002 | 4.1E-35 | C | G | 0.592 | 152.865 |
|  | rs921919 | -0.037 | 0.002 | 2.5E-68 | A | G | 0.669 | 305.103 |
|  | rs35028188 | -0.013 | 0.002 | 4.5E-10 | T | TA | 0.612 | 38.884 |
|  | rs61352607 | 0.028 | 0.002 | 1.1E-33 | T | G | 0.241 | 146.314 |
|  | rs10745954 | -0.014 | 0.002 | 2.5E-13 | G | A | 0.480 | 53.563 |
|  | rs10774439 | 0.016 | 0.003 | 1.3E-10 | A | G | 0.815 | 41.370 |
|  | rs4883201 | -0.025 | 0.003 | 5.3E-15 | G | A | 0.102 | 61.153 |
|  | rs7304603 | -0.013 | 0.002 | 1.1E-10 | C | T | 0.538 | 41.654 |
|  | rs10748165 | 0.011 | 0.002 | 0.000000021 | T | C | 0.504 | 31.400 |
|  | rs12229372 | 0.030 | 0.003 | 2.4E-19 | C | T | 0.103 | 80.872 |
|  | rs76428106 | -0.061 | 0.009 | 1.3E-11 | C | T | 0.013 | 45.791 |
|  | rs11452128 | -0.026 | 0.005 | 0.000000011 | C | T | 0.050 | 32.659 |
|  | rs35639749 | -0.052 | 0.009 | 6.6E-09 | A | AC | 0.987 | 33.663 |
|  | rs79506257 | 0.028 | 0.005 | 1.2E-09 | A | G | 0.048 | 36.949 |
|  | rs35580554 | -0.014 | 0.002 | 8.1E-13 | A | ATATT | 0.604 | 51.261 |
|  | rs9604045 | 0.015 | 0.002 | 5.8E-11 | T | G | 0.254 | 42.880 |
|  | rs2494747 | -0.032 | 0.002 | 4.3E-57 | T | G | 0.616 | 253.601 |
|  | rs61980899 | -0.014 | 0.002 | 1.5E-12 | C | T | 0.538 | 50.016 |
|  | rs367677 | 0.017 | 0.002 | 4.1E-14 | G | A | 0.240 | 57.100 |
|  | rs13379043 | 0.022 | 0.002 | 1.5E-22 | C | T | 0.280 | 95.467 |
|  | rs7147511 | -0.015 | 0.002 | 1.5E-13 | T | C | 0.495 | 54.512 |
|  | rs12436555 | -0.015 | 0.003 | 0.000000018 | A | G | 0.173 | 31.686 |
|  | rs111849006 | 0.015 | 0.003 | 0.000000017 | A | G | 0.181 | 31.865 |
|  | rs2111705 | -0.015 | 0.002 | 3.7E-14 | A | G | 0.544 | 57.319 |
|  | rs7170463 | 0.016 | 0.002 | 2.2E-14 | G | A | 0.311 | 58.386 |
|  | rs8028785 | 0.015 | 0.003 | 7.7E-09 | C | T | 0.183 | 33.357 |
|  | rs150844304 | -0.075 | 0.006 | 2E-34 | C | A | 0.026 | 149.747 |
|  | rs1601933 | -0.084 | 0.002 | 1E-200 | T | C | 0.467 | 1781.378 |
|  | rs12898210 | -0.049 | 0.003 | 6.4E-60 | G | T | 0.122 | 266.539 |
|  | rs11632618 | 0.108 | 0.004 | 1.1E-173 | A | G | 0.070 | 789.319 |
|  | rs80236739 | -0.032 | 0.005 | 8.2E-13 | G | A | 0.050 | 51.231 |
|  | rs34180494 | -0.013 | 0.002 | 4.8E-09 | C | A | 0.274 | 34.277 |
|  | rs28362901 | -0.025 | 0.003 | 1E-12 | A | C | 0.088 | 50.792 |
|  | rs117399007 | -0.029 | 0.005 | 2.5E-09 | T | C | 0.043 | 35.503 |
|  | rs1318175 | -0.078 | 0.003 | 3.4E-186 | T | C | 0.160 | 846.918 |
|  | rs1037117 | 0.016 | 0.002 | 9.5E-13 | A | G | 0.255 | 50.944 |
|  | rs71380411 | -0.011 | 0.002 | 0.000000036 | T | C | 0.424 | 30.342 |
|  | rs75152587 | -0.080 | 0.009 | 1.2E-19 | T | G | 0.013 | 82.276 |
|  | rs2305696 | -0.018 | 0.002 | 3.9E-21 | T | C | 0.486 | 89.035 |
|  | rs4330777 | -0.017 | 0.002 | 2.8E-17 | A | G | 0.475 | 71.446 |
|  | rs11641548 | -0.013 | 0.002 | 3.6E-11 | C | A | 0.440 | 43.840 |
|  | rs74025321 | -0.030 | 0.004 | 1.1E-15 | G | A | 0.074 | 64.177 |
|  | rs35909200 | 0.019 | 0.003 | 1.7E-12 | G | T | 0.157 | 49.804 |
|  | rs34955778 | -0.012 | 0.002 | 3.7E-10 | C | T | 0.419 | 39.241 |
|  | rs4784709 | -0.062 | 0.005 | 7.4E-36 | A | T | 0.959 | 156.279 |
|  | rs33042 | 0.017 | 0.002 | 2.5E-13 | A | G | 0.246 | 53.597 |
|  | rs144475339 | 0.058 | 0.010 | 0.000000029 | C | G | 0.011 | 30.742 |
|  | rs79984435 | -0.086 | 0.003 | 4.2E-142 | A | G | 0.092 | 644.155 |
|  | rs9989419 | 0.115 | 0.002 | 1E-200 | G | A | 0.606 | 3325.539 |
|  | rs200561116 | -0.057 | 0.003 | 1.4E-75 | A | AT | 0.886 | 338.362 |
|  | rs12943517 | -0.012 | 0.002 | 0.000000024 | A | G | 0.311 | 31.120 |
|  | rs3818716 | -0.013 | 0.002 | 5.2E-10 | T | C | 0.365 | 38.606 |
|  | rs145947882 | -0.136 | 0.006 | 6.9E-105 | C | A | 0.026 | 473.072 |
|  | rs7216643 | -0.022 | 0.002 | 2.9E-21 | T | C | 0.224 | 89.577 |
|  | rs78608249 | -0.016 | 0.002 | 8E-13 | T | C | 0.290 | 51.281 |
|  | rs6505176 | -0.017 | 0.002 | 8.6E-17 | G | T | 0.332 | 69.273 |
|  | rs7503353 | -0.014 | 0.002 | 7.3E-12 | T | G | 0.532 | 46.958 |
|  | rs4795386 | 0.026 | 0.002 | 5.1E-31 | G | A | 0.724 | 134.143 |
|  | rs145773899 | 0.030 | 0.002 | 2.3E-49 | C | T | 0.543 | 218.160 |
|  | rs2071379 | -0.021 | 0.002 | 8.1E-27 | G | A | 0.595 | 114.934 |
|  | rs34138141 | -0.018 | 0.002 | 1.3E-16 | T | G | 0.281 | 68.466 |
|  | rs11870735 | -0.015 | 0.003 | 2.2E-09 | T | C | 0.181 | 35.758 |
|  | rs3760230 | -0.014 | 0.002 | 7.3E-12 | G | C | 0.579 | 46.947 |
|  | rs2298624 | 0.032 | 0.003 | 2E-28 | T | C | 0.132 | 122.325 |
|  | rs8098618 | 0.012 | 0.002 | 5.1E-09 | T | C | 0.479 | 34.155 |
|  | rs78965095 | 0.044 | 0.007 | 3.5E-10 | T | C | 0.020 | 39.397 |
|  | rs77960347 | 0.336 | 0.009 | 1E-200 | G | A | 0.013 | 1542.059 |
|  | rs4632228 | -0.020 | 0.002 | 1.6E-16 | T | G | 0.209 | 68.046 |
|  | rs150237291 | 0.049 | 0.007 | 2.3E-13 | C | T | 0.022 | 53.750 |
|  | rs681869 | -0.017 | 0.002 | 1.5E-14 | T | C | 0.704 | 59.114 |
|  | rs8086351 | 0.096 | 0.003 | 1E-200 | G | C | 0.824 | 1392.506 |
|  | rs7238484 | -0.021 | 0.002 | 5.3E-22 | T | G | 0.268 | 92.966 |
|  | rs76213248 | 0.021 | 0.002 | 1.8E-26 | T | C | 0.410 | 113.323 |
|  | rs429358 | -0.103 | 0.003 | 1E-200 | C | T | 0.154 | 1426.265 |
|  | rs12462109 | -0.013 | 0.002 | 6.4E-10 | T | C | 0.287 | 38.196 |
|  | rs4807462 | -0.011 | 0.002 | 0.000000025 | T | A | 0.360 | 31.042 |
|  | rs367070 | 0.039 | 0.002 | 8.4E-62 | G | A | 0.225 | 275.183 |
|  | rs10404380 | -0.026 | 0.002 | 1.6E-37 | A | C | 0.605 | 163.883 |
|  | rs2965169 | 0.019 | 0.002 | 7.6E-21 | C | A | 0.389 | 87.692 |
|  | rs144311893 | 0.123 | 0.007 | 1.1E-70 | T | C | 0.022 | 316.015 |
|  | rs12977604 | 0.037 | 0.002 | 2E-71 | G | C | 0.499 | 319.346 |
|  | rs737338 | -0.108 | 0.005 | 2.3E-91 | T | C | 0.035 | 410.939 |
|  | rs7251640 | 0.015 | 0.002 | 1.3E-09 | C | T | 0.194 | 36.787 |
|  | rs8103728 | 0.023 | 0.002 | 7.3E-28 | G | C | 0.670 | 119.724 |
|  | rs2424993 | -0.016 | 0.002 | 4.9E-14 | C | G | 0.698 | 56.774 |
|  | rs6062510 | -0.015 | 0.002 | 3.5E-13 | C | G | 0.674 | 52.901 |
|  | rs11481448 | 0.011 | 0.002 | 5.9E-09 | A | AT | 0.497 | 33.855 |
|  | rs201877296 | -0.058 | 0.006 | 1.2E-25 | A | T | 0.037 | 109.553 |
|  | rs1800961 | -0.149 | 0.006 | 1.3E-152 | T | C | 0.031 | 692.422 |
|  | rs144033177 | -0.049 | 0.008 | 8.9E-10 | C | A | 0.016 | 37.555 |
|  | rs1132274 | -0.029 | 0.003 | 3.2E-26 | A | C | 0.154 | 112.215 |
|  | rs6057911 | 0.019 | 0.003 | 2E-10 | G | C | 0.131 | 40.473 |
|  | rs2256720 | 0.012 | 0.002 | 7.4E-10 | C | T | 0.444 | 37.907 |
|  | rs4239651 | 0.026 | 0.002 | 1.4E-27 | C | T | 0.794 | 118.392 |
|  | rs9977268 | -0.018 | 0.002 | 1.5E-13 | T | C | 0.199 | 54.619 |
|  | rs235314 | -0.021 | 0.002 | 1.5E-27 | T | C | 0.532 | 118.251 |
|  | rs133015 | 0.020 | 0.002 | 5.6E-24 | G | C | 0.440 | 101.971 |
|  | rs11089620 | -0.032 | 0.003 | 1.7E-37 | G | C | 0.189 | 163.792 |
|  | rs4820346 | 0.013 | 0.002 | 1.1E-09 | G | C | 0.693 | 37.070 |
|  | rs2294915 | -0.020 | 0.002 | 1E-17 | T | C | 0.232 | 73.504 |
|  | rs9608972 | -0.017 | 0.002 | 2.8E-14 | C | T | 0.241 | 57.856 |
| **apoB** | rs1003533 | -0.020 | 0.003 | 5.3E-14 | T | C | 0.188 | 56.621 |
|  | rs10087526 | 0.019 | 0.003 | 6.2E-13 | G | T | 0.199 | 51.770 |
|  | rs10127775 | -0.023 | 0.002 | 4.2E-28 | T | A | 0.606 | 120.830 |
|  | rs10151436 | -0.019 | 0.003 | 9.8E-09 | T | A | 0.112 | 32.874 |
|  | rs10201242 | -0.023 | 0.004 | 0.000000012 | A | G | 0.074 | 32.455 |
|  | rs10448340 | -0.017 | 0.002 | 2E-14 | G | T | 0.320 | 58.488 |
|  | rs10794579 | 0.014 | 0.002 | 6.8E-11 | C | T | 0.575 | 42.590 |
|  | rs10832963 | 0.022 | 0.002 | 3.8E-20 | G | T | 0.744 | 84.533 |
|  | rs10953298 | -0.017 | 0.002 | 3.7E-12 | T | C | 0.236 | 48.289 |
|  | rs11047939 | 0.015 | 0.003 | 0.000000001 | A | G | 0.225 | 37.297 |
|  | rs11057397 | -0.018 | 0.002 | 7.8E-17 | T | C | 0.337 | 69.458 |
|  | rs11057837 | 0.022 | 0.003 | 2.5E-10 | T | C | 0.103 | 40.050 |
|  | rs11065384 | -0.022 | 0.002 | 4.9E-22 | C | T | 0.690 | 93.111 |
|  | rs111928762 | 0.052 | 0.005 | 3.2E-22 | G | A | 0.040 | 93.999 |
|  | rs112220485 | 0.021 | 0.004 | 0.000000039 | C | T | 0.086 | 30.189 |
|  | rs112758337 | -0.021 | 0.003 | 2.1E-15 | A | G | 0.186 | 62.984 |
|  | rs113177823 | -0.041 | 0.005 | 7.2E-19 | A | G | 0.054 | 78.709 |
|  | rs114165349 | 0.090 | 0.007 | 7.7E-39 | C | G | 0.023 | 169.913 |
|  | rs11568318 | 0.029 | 0.004 | 4.2E-12 | A | C | 0.066 | 48.013 |
|  | rs115692156 | -0.112 | 0.012 | 5.1E-21 | G | A | 0.009 | 88.506 |
|  | rs11591147 | -0.346 | 0.008 | 1E-200 | T | G | 0.017 | 1917.296 |
|  | rs11601507 | 0.041 | 0.004 | 1.1E-24 | A | C | 0.069 | 105.282 |
|  | rs116093870 | 0.026 | 0.004 | 2.3E-10 | C | T | 0.068 | 40.168 |
|  | rs11621792 | 0.021 | 0.002 | 1.3E-23 | T | C | 0.453 | 100.384 |
|  | rs116734477 | -0.063 | 0.005 | 2.5E-33 | T | C | 0.041 | 144.694 |
|  | rs11673631 | 0.039 | 0.005 | 3.2E-14 | C | G | 0.043 | 57.622 |
|  | rs11709868 | -0.016 | 0.002 | 9.5E-12 | T | G | 0.297 | 46.421 |
|  | rs117733303 | 0.086 | 0.008 | 3.6E-29 | G | A | 0.018 | 125.682 |
|  | rs118039278 | 0.087 | 0.004 | 2.2E-111 | A | G | 0.079 | 502.947 |
|  | rs11901691 | 0.014 | 0.002 | 1.5E-11 | A | C | 0.577 | 45.567 |
|  | rs12054451 | 0.016 | 0.002 | 2.1E-11 | G | T | 0.259 | 44.855 |
|  | rs12078100 | 0.015 | 0.002 | 1.2E-12 | G | C | 0.623 | 50.563 |
|  | rs12208357 | 0.063 | 0.004 | 1E-53 | T | C | 0.070 | 238.062 |
|  | rs12469941 | 0.013 | 0.002 | 1.5E-10 | T | C | 0.401 | 40.969 |
|  | rs12471768 | 0.014 | 0.002 | 8.8E-10 | C | T | 0.704 | 37.570 |
|  | rs1250258 | 0.014 | 0.002 | 1.9E-09 | T | C | 0.737 | 36.083 |
|  | rs12597418 | 0.018 | 0.002 | 2.9E-17 | A | G | 0.368 | 71.391 |
|  | rs12603290 | -0.029 | 0.002 | 9.8E-44 | C | T | 0.515 | 192.333 |
|  | rs12691088 | 0.246 | 0.008 | 1E-200 | A | G | 0.021 | 1022.709 |
|  | rs1277762 | 0.017 | 0.003 | 2.2E-09 | T | C | 0.843 | 35.796 |
|  | rs12916 | 0.055 | 0.002 | 5.2E-148 | C | T | 0.400 | 671.287 |
|  | rs13076933 | -0.018 | 0.002 | 1.8E-14 | G | T | 0.259 | 58.757 |
|  | rs13108218 | -0.023 | 0.002 | 1.6E-26 | G | A | 0.615 | 113.534 |
|  | rs13247874 | -0.022 | 0.003 | 1.4E-16 | T | C | 0.197 | 68.275 |
|  | rs13379043 | -0.014 | 0.002 | 1.1E-09 | C | T | 0.280 | 37.102 |
|  | rs1358980 | 0.015 | 0.002 | 1.1E-12 | T | C | 0.483 | 50.632 |
|  | rs13702 | -0.030 | 0.002 | 7.8E-38 | C | T | 0.288 | 165.318 |
|  | rs138026891 | -0.069 | 0.010 | 1.6E-11 | T | C | 0.011 | 45.379 |
|  | rs138354 | -0.012 | 0.002 | 6.9E-09 | C | T | 0.535 | 33.551 |
|  | rs138692741 | 0.071 | 0.006 | 3E-36 | T | C | 0.036 | 158.092 |
|  | rs141469619 | 0.079 | 0.011 | 1.6E-12 | G | A | 0.010 | 49.959 |
|  | rs143020224 | -0.165 | 0.003 | 1E-200 | G | C | 0.119 | 2703.568 |
|  | rs144926613 | -0.015 | 0.002 | 4.2E-11 | T | TTGGAGGGCAGACTAGCCCAGGCCC | 0.672 | 43.498 |
|  | rs145730801 | 0.037 | 0.005 | 3.6E-13 | C | T | 0.044 | 52.871 |
|  | rs146534110 | 0.067 | 0.009 | 1.8E-13 | T | G | 0.013 | 54.210 |
|  | rs147539187 | -0.023 | 0.004 | 0.000000011 | G | C | 0.074 | 32.628 |
|  | rs148150904 | 0.016 | 0.003 | 1.6E-09 | TTAAAG | T | 0.190 | 36.406 |
|  | rs148601586 | 0.184 | 0.009 | 5.9E-89 | G | C | 0.013 | 399.872 |
|  | rs148933445 | -0.573 | 0.008 | 1E-200 | A | G | 0.022 | 5701.635 |
|  | rs150474434 | -0.034 | 0.003 | 2.8E-23 | A | G | 0.101 | 98.789 |
|  | rs150503754 | -0.052 | 0.009 | 2.4E-09 | C | T | 0.015 | 35.617 |
|  | rs150820726 | 0.072 | 0.011 | 1.1E-11 | T | A | 0.010 | 46.148 |
|  | rs1556562 | 0.016 | 0.002 | 1.1E-10 | T | G | 0.790 | 41.667 |
|  | rs1561139 | -0.013 | 0.002 | 4.2E-10 | T | G | 0.424 | 39.008 |
|  | rs17036085 | -0.068 | 0.009 | 4E-13 | G | A | 0.012 | 52.664 |
|  | rs17050272 | -0.024 | 0.002 | 3.8E-31 | A | G | 0.409 | 134.739 |
|  | rs174564 | -0.045 | 0.002 | 1E-94 | G | A | 0.349 | 426.380 |
|  | rs17476364 | -0.021 | 0.003 | 4E-10 | C | T | 0.108 | 39.108 |
|  | rs17569873 | 0.018 | 0.003 | 1.6E-12 | T | C | 0.201 | 49.978 |
|  | rs1801689 | 0.065 | 0.006 | 9.9E-27 | C | A | 0.031 | 114.538 |
|  | rs183130 | -0.048 | 0.002 | 1.3E-102 | T | C | 0.324 | 462.635 |
|  | rs188608977 | 0.062 | 0.010 | 2.1E-09 | T | G | 0.011 | 35.894 |
|  | rs1888488 | 0.017 | 0.002 | 1.5E-16 | T | C | 0.565 | 68.161 |
|  | rs200046586 | 0.412 | 0.008 | 1E-200 | C | CA | 0.981 | 2801.294 |
|  | rs2043085 | -0.019 | 0.002 | 1.2E-19 | C | T | 0.612 | 82.176 |
|  | rs2068888 | -0.024 | 0.002 | 5.2E-31 | A | G | 0.451 | 134.098 |
|  | rs2073547 | 0.031 | 0.003 | 1.1E-31 | G | A | 0.184 | 137.185 |
|  | rs2137234 | 0.015 | 0.003 | 5.7E-09 | C | T | 0.195 | 33.936 |
|  | rs2160994 | 0.017 | 0.002 | 2.1E-15 | C | T | 0.647 | 62.927 |
|  | rs2199048 | -0.013 | 0.002 | 3.8E-09 | G | A | 0.314 | 34.733 |
|  | rs2238162 | -0.023 | 0.002 | 1.5E-27 | T | C | 0.523 | 118.305 |
|  | rs224391 | -0.017 | 0.002 | 2E-12 | C | G | 0.227 | 49.445 |
|  | rs2256814 | 0.015 | 0.003 | 0.000000029 | A | G | 0.198 | 30.792 |
|  | rs2287622 | -0.020 | 0.002 | 7.7E-22 | G | A | 0.603 | 92.233 |
|  | rs2446066 | 0.016 | 0.003 | 0.000000012 | T | G | 0.175 | 32.411 |
|  | rs2519093 | 0.041 | 0.003 | 3.9E-53 | T | C | 0.185 | 235.427 |
|  | rs2618566 | -0.030 | 0.002 | 2E-43 | T | G | 0.660 | 190.906 |
|  | rs2737263 | -0.019 | 0.002 | 1.2E-15 | T | G | 0.280 | 64.128 |
|  | rs2738447 | 0.044 | 0.002 | 9.9E-97 | C | A | 0.593 | 435.587 |
|  | rs2761311 | 0.012 | 0.002 | 0.00000001 | T | C | 0.572 | 32.757 |
|  | rs278981 | 0.015 | 0.002 | 6.1E-10 | C | T | 0.758 | 38.299 |
|  | rs2807854 | 0.016 | 0.002 | 1.4E-13 | C | T | 0.671 | 54.642 |
|  | rs28406917 | 0.013 | 0.002 | 2E-10 | T | C | 0.428 | 40.436 |
|  | rs28590710 | -0.018 | 0.003 | 9.1E-11 | A | G | 0.197 | 42.007 |
|  | rs28601761 | -0.073 | 0.002 | 1E-200 | G | C | 0.419 | 1162.066 |
|  | rs28814720 | 0.012 | 0.002 | 0.000000043 | G | A | 0.520 | 30.007 |
|  | rs3127580 | 0.040 | 0.003 | 3E-45 | T | C | 0.155 | 199.259 |
|  | rs34042070 | 0.049 | 0.003 | 1.9E-76 | G | C | 0.188 | 342.376 |
|  | rs34596921 | 0.029 | 0.002 | 3.3E-43 | T | TA | 0.580 | 189.946 |
|  | rs34869792 | -0.042 | 0.007 | 0.000000018 | A | T | 0.032 | 31.713 |
|  | rs35081008 | -0.031 | 0.003 | 7.8E-27 | T | C | 0.148 | 115.013 |
|  | rs35278712 | 0.020 | 0.002 | 4.6E-18 | GAAAT | G | 0.712 | 75.055 |
|  | rs35465692 | -0.025 | 0.004 | 5.2E-09 | AT | A | 0.908 | 34.114 |
|  | rs35583283 | 0.016 | 0.003 | 0.000000023 | C | G | 0.155 | 31.192 |
|  | rs35764292 | 0.051 | 0.005 | 1.3E-21 | G | A | 0.040 | 91.256 |
|  | rs35882350 | 0.014 | 0.002 | 0.00000001 | G | A | 0.261 | 32.801 |
|  | rs35980001 | -0.024 | 0.003 | 1.4E-21 | G | GC | 0.787 | 91.014 |
|  | rs3746337 | 0.016 | 0.002 | 5.1E-14 | T | C | 0.489 | 56.698 |
|  | rs374929797 | -0.012 | 0.002 | 8.3E-09 | C | T | 0.443 | 33.202 |
|  | rs375972689 | 0.209 | 0.010 | 3.5E-95 | G | T | 0.011 | 428.499 |
|  | rs3780181 | -0.026 | 0.004 | 4.7E-10 | G | A | 0.068 | 38.794 |
|  | rs3822855 | 0.015 | 0.002 | 2.4E-12 | T | G | 0.402 | 49.149 |
|  | rs3823376 | 0.016 | 0.002 | 2.5E-15 | T | C | 0.502 | 62.648 |
|  | rs3860846 | 0.014 | 0.002 | 4.1E-09 | T | C | 0.275 | 34.572 |
|  | rs4052908 | -0.013 | 0.002 | 0.000000007 | AATT | A | 0.636 | 33.535 |
|  | rs4307732 | 0.048 | 0.003 | 5.8E-46 | A | G | 0.106 | 202.537 |
|  | rs4470903 | 0.035 | 0.003 | 2.6E-42 | G | C | 0.215 | 185.857 |
|  | rs454715 | -0.024 | 0.002 | 2.4E-29 | G | T | 0.583 | 126.518 |
|  | rs45537841 | -0.016 | 0.003 | 4.1E-09 | T | C | 0.181 | 34.566 |
|  | rs4671050 | -0.020 | 0.002 | 1E-18 | T | G | 0.316 | 78.045 |
|  | rs4687614 | 0.027 | 0.005 | 3.7E-09 | A | G | 0.945 | 34.769 |
|  | rs4689088 | 0.012 | 0.002 | 5.9E-09 | A | G | 0.619 | 33.867 |
|  | rs472495 | 0.041 | 0.002 | 1.3E-78 | T | G | 0.649 | 352.317 |
|  | rs4782568 | -0.018 | 0.002 | 3.2E-18 | G | C | 0.451 | 75.765 |
|  | rs478975 | 0.014 | 0.002 | 7.9E-11 | A | G | 0.643 | 42.281 |
|  | rs4803818 | 0.042 | 0.007 | 1.3E-09 | T | C | 0.023 | 36.880 |
|  | rs4935356 | 0.025 | 0.003 | 6.6E-22 | A | T | 0.244 | 92.549 |
|  | rs546240 | -0.012 | 0.002 | 0.000000031 | T | C | 0.620 | 30.616 |
|  | rs556107 | 0.036 | 0.002 | 1.3E-66 | T | C | 0.523 | 297.210 |
|  | rs55637835 | -0.019 | 0.003 | 6.1E-09 | T | C | 0.121 | 33.799 |
|  | rs55714927 | -0.032 | 0.003 | 5.1E-34 | T | C | 0.190 | 147.841 |
|  | rs55831924 | 0.018 | 0.002 | 4.8E-17 | T | C | 0.361 | 70.421 |
|  | rs57754494 | 0.026 | 0.004 | 5.5E-14 | A | G | 0.116 | 56.542 |
|  | rs5776986 | -0.020 | 0.003 | 2.1E-13 | G | GT | 0.811 | 53.918 |
|  | rs581080 | 0.019 | 0.003 | 6.4E-12 | C | G | 0.819 | 47.189 |
|  | rs58148580 | 0.021 | 0.003 | 9.6E-11 | T | C | 0.110 | 41.907 |
|  | rs5849920 | -0.015 | 0.002 | 7.4E-11 | G | GT | 0.364 | 42.423 |
|  | rs59328596 | -0.021 | 0.003 | 6.6E-13 | A | G | 0.148 | 51.667 |
|  | rs6072279 | 0.029 | 0.002 | 8.7E-45 | A | G | 0.474 | 197.151 |
|  | rs6073958 | 0.042 | 0.003 | 2.5E-59 | C | T | 0.199 | 263.802 |
|  | rs6129620 | -0.025 | 0.002 | 8.7E-30 | A | T | 0.338 | 128.498 |
|  | rs61754230 | 0.043 | 0.007 | 6.8E-09 | T | C | 0.020 | 33.585 |
|  | rs62119267 | -0.335 | 0.007 | 1E-200 | C | A | 0.022 | 2233.867 |
|  | rs62122481 | 0.084 | 0.002 | 1E-200 | A | C | 0.377 | 1537.840 |
|  | rs6426328 | 0.012 | 0.002 | 0.000000004 | T | G | 0.490 | 34.638 |
|  | rs6475606 | -0.017 | 0.002 | 7.2E-17 | T | C | 0.484 | 69.620 |
|  | rs6560499 | -0.012 | 0.002 | 0.000000009 | A | G | 0.576 | 33.057 |
|  | rs6602909 | 0.021 | 0.002 | 4.6E-22 | C | T | 0.328 | 93.254 |
|  | rs6657811 | -0.128 | 0.003 | 1E-200 | T | A | 0.130 | 1713.258 |
|  | rs6667939 | 0.014 | 0.002 | 1.2E-09 | T | C | 0.719 | 36.966 |
|  | rs6689611 | -0.132 | 0.010 | 3.9E-44 | A | G | 0.012 | 194.156 |
|  | rs67038483 | 0.028 | 0.005 | 0.00000003 | T | C | 0.045 | 30.691 |
|  | rs6874202 | 0.031 | 0.002 | 1.6E-47 | C | T | 0.634 | 209.709 |
|  | rs6940814 | -0.020 | 0.002 | 3E-21 | G | A | 0.587 | 89.525 |
|  | rs7012637 | 0.020 | 0.002 | 1.3E-21 | A | G | 0.474 | 91.254 |
|  | rs7108486 | -0.043 | 0.007 | 5.2E-10 | C | T | 0.024 | 38.613 |
|  | rs71140104 | -0.013 | 0.002 | 1.4E-09 | C | CA | 0.440 | 36.722 |
|  | rs71311871 | -0.026 | 0.004 | 2.9E-12 | G | A | 0.083 | 48.760 |
|  | rs71637095 | 0.015 | 0.002 | 4.2E-12 | G | T | 0.341 | 48.046 |
|  | rs7249565 | 0.015 | 0.002 | 2.9E-12 | A | G | 0.415 | 48.779 |
|  | rs72631343 | -0.028 | 0.003 | 2.3E-19 | G | C | 0.129 | 80.949 |
|  | rs72663045 | 0.042 | 0.007 | 0.000000008 | G | T | 0.021 | 33.268 |
|  | rs72823013 | -0.023 | 0.003 | 3.2E-13 | A | G | 0.126 | 53.062 |
|  | rs72848251 | 0.035 | 0.003 | 1.5E-38 | A | G | 0.195 | 168.659 |
|  | rs73075609 | 0.049 | 0.006 | 6.2E-14 | T | C | 0.027 | 56.310 |
|  | rs74454529 | 0.028 | 0.004 | 1.3E-10 | A | G | 0.060 | 41.325 |
|  | rs75331444 | -0.090 | 0.004 | 2E-103 | A | G | 0.066 | 466.392 |
|  | rs7569317 | 0.018 | 0.002 | 2.6E-18 | C | T | 0.531 | 76.163 |
|  | rs7590687 | -0.043 | 0.004 | 3.4E-27 | C | T | 0.923 | 116.668 |
|  | rs7603427 | 0.014 | 0.002 | 6.6E-11 | T | C | 0.533 | 42.639 |
|  | rs76186504 | -0.116 | 0.007 | 3.3E-69 | T | C | 0.025 | 309.175 |
|  | rs7707394 | 0.035 | 0.002 | 1.4E-59 | A | G | 0.357 | 264.987 |
|  | rs77083979 | -0.019 | 0.003 | 0.000000002 | A | G | 0.133 | 35.969 |
|  | rs7734476 | 0.020 | 0.002 | 1.2E-21 | A | G | 0.550 | 91.317 |
|  | rs7746081 | -0.023 | 0.002 | 6.7E-24 | A | G | 0.304 | 101.622 |
|  | rs77498041 | -0.016 | 0.003 | 8.5E-10 | A | G | 0.191 | 37.648 |
|  | rs77542162 | 0.116 | 0.007 | 2.5E-61 | G | A | 0.022 | 273.020 |
|  | rs79149284 | 0.034 | 0.005 | 3.2E-10 | T | C | 0.038 | 39.545 |
|  | rs8016418 | 0.020 | 0.002 | 4.6E-20 | A | T | 0.375 | 84.135 |
|  | rs80276949 | 0.042 | 0.007 | 2.1E-09 | A | G | 0.023 | 35.893 |
|  | rs8107974 | -0.091 | 0.004 | 4.5E-121 | T | A | 0.076 | 547.478 |
|  | rs9297994 | -0.029 | 0.002 | 6.2E-40 | A | G | 0.665 | 174.932 |
|  | rs9482772 | 0.018 | 0.002 | 2.2E-17 | C | T | 0.448 | 71.956 |
|  | rs9496567 | -0.019 | 0.002 | 3.8E-15 | A | G | 0.244 | 61.790 |
|  | rs9616822 | 0.016 | 0.002 | 2.7E-13 | A | G | 0.351 | 53.396 |
|  | rs964184 | -0.077 | 0.003 | 2.5E-138 | C | G | 0.867 | 626.776 |
|  | rs969075 | 0.014 | 0.002 | 4.4E-10 | C | T | 0.665 | 38.912 |
|  | rs9834932 | -0.032 | 0.004 | 3.9E-18 | G | A | 0.089 | 75.394 |
|  | rs9884390 | 0.023 | 0.002 | 7.8E-21 | C | T | 0.234 | 87.651 |
|  | rs9894946 | -0.017 | 0.003 | 3.1E-09 | G | A | 0.841 | 35.139 |

**Supplementary Table S2:** Information of genetic instrumental variants associated with LDL cholesterol located within 100 kb windows from gene HMGCR, PCSK9, NPC1L1 or APOB.

| **Gene** | **SNP** | **Beta** | **SE** | ***p*** | **Effect allele** | **other allele** | **EAF** | **mean F-statistic** |
| --- | --- | --- | --- | --- | --- | --- | --- | --- |
| ***HMGCR*** |  |  |  |  |  |  |  |  |
|  | rs75240579 | -0.037 | 0.005 | 2.19989E-14 | T | C | 0.048 | 58.336 |
|  | rs2006760 | 0.036 | 0.003 | 2.99985E-42 | G | C | 0.205 | 185.521 |
|  | rs62366588 | -0.027 | 0.004 | 3.69999E-10 | A | C | 0.066 | 39.239 |
|  | rs141642272 | 0.053 | 0.007 | 3.69999E-16 | C | G | 0.027 | 66.382 |
|  | rs55727654 | 0.042 | 0.003 | 6.89922E-47 | A | G | 0.149 | 206.775 |
|  | rs111353455 | 0.024 | 0.004 | 6.00067E-11 | A | G | 0.086 | 42.825 |
|  | rs2303152 | 0.033 | 0.003 | 4.40048E-22 | A | G | 0.101 | 93.347 |
|  | rs116153450 | -0.030 | 0.005 | 1.2E-09 | A | C | 0.046 | 36.986 |
|  | rs12916 | 0.062 | 0.002 | 1.6982E-187 | C | T | 0.401 | 852.850 |
|  | rs17562727 | 0.039 | 0.006 | 5.30005E-10 | C | T | 0.028 | 38.580 |
|  | rs80324692 | -0.026 | 0.004 | 1.39991E-11 | T | C | 0.081 | 45.620 |
|  | rs115845757 | 0.049 | 0.008 | 6.1E-10 | A | G | 0.019 | 38.282 |
|  | rs17648121 | 0.062 | 0.006 | 1.39991E-23 | T | C | 0.030 | 100.156 |
|  | rs140092661 | 0.033 | 0.006 | 0.000000015 | T | A | 0.034 | 32.117 |
|  | rs12659331 | 0.025 | 0.005 | 4.20001E-08 | C | A | 0.054 | 30.040 |
|  | rs72633963 | 0.056 | 0.003 | 4.90004E-71 | A | G | 0.124 | 317.555 |
|  | rs10051965 | 0.041 | 0.002 | 5.40008E-80 | T | C | 0.370 | 358.685 |
|  | rs35122945 | -0.028 | 0.004 | 3.29989E-11 | C | A | 0.067 | 43.991 |
|  | rs4703665 | 0.024 | 0.003 | 1.9002E-16 | C | T | 0.849 | 67.676 |
| ***PCSK9*** |  |  |  |  |  |  |  |  |
|  | rs6691964 | -0.023 | 0.004 | 5.79963E-11 | A | G | 0.092 | 42.893 |
|  | rs556369867 | 0.018 | 0.002 | 4.79954E-13 | T | C | 0.331 | 52.286 |
|  | rs72909541 | -0.033 | 0.005 | 2.70023E-11 | T | C | 0.046 | 44.376 |
|  | rs150119739 | 0.045 | 0.005 | 3.19963E-18 | A | G | 0.045 | 75.739 |
|  | rs7525503 | 0.045 | 0.008 | 0.000000002 | T | G | 0.020 | 35.954 |
|  | rs11587071 | -0.028 | 0.003 | 5.30029E-24 | T | C | 0.169 | 102.092 |
|  | rs10493176 | -0.053 | 0.004 | 2.60016E-41 | G | T | 0.076 | 181.272 |
|  | rs3976734 | -0.030 | 0.002 | 1.10002E-37 | G | A | 0.375 | 164.597 |
|  | rs200730299 | -0.054 | 0.003 | 5.10035E-85 | C | A | 0.195 | 381.779 |
|  | rs17192725 | 0.031 | 0.004 | 6.4003E-17 | A | G | 0.095 | 69.835 |
|  | rs17111503 | 0.041 | 0.002 | 1E-66 | G | A | 0.268 | 297.765 |
|  | rs7546522 | -0.017 | 0.003 | 0.000000012 | T | C | 0.155 | 32.412 |
|  | rs2483205 | -0.030 | 0.002 | 2.90001E-43 | T | C | 0.439 | 190.203 |
|  | rs11583974 | 0.031 | 0.005 | 1.2E-09 | A | G | 0.042 | 37.003 |
|  | rs56349475 | -0.048 | 0.007 | 1.39991E-12 | C | T | 0.025 | 50.178 |
|  | rs79396670 | -0.034 | 0.006 | 2.1E-09 | A | G | 0.035 | 35.845 |
|  | rs146273942 | -0.054 | 0.007 | 8.69961E-14 | A | G | 0.023 | 55.638 |
|  | rs2479420 | -0.028 | 0.002 | 9.8992E-33 | T | C | 0.738 | 141.959 |
|  | rs11810371 | -0.029 | 0.005 | 6.4E-09 | A | G | 0.044 | 33.707 |
|  | rs11591147 | -0.348 | 0.008 | 1E-200 | T | G | 0.017 | 1930.426 |
|  | rs11206513 | 0.032 | 0.002 | 3.19963E-49 | T | C | 0.601 | 217.477 |
|  | rs11206517 | 0.068 | 0.006 | 1E-31 | G | T | 0.033 | 137.279 |
|  | rs2495517 | 0.018 | 0.003 | 5.79963E-12 | G | A | 0.794 | 47.387 |
|  | rs12732125 | -0.103 | 0.007 | 1E-44 | T | C | 0.020 | 196.797 |
|  | rs2479395 | 0.013 | 0.002 | 0.000000015 | C | T | 0.668 | 32.116 |
|  | rs77875082 | 0.048 | 0.006 | 1.80011E-15 | A | G | 0.032 | 63.233 |
|  | rs41294821 | -0.039 | 0.007 | 4.20001E-08 | T | C | 0.023 | 30.042 |
|  | rs472495 | 0.043 | 0.002 | 7.29962E-85 | T | G | 0.649 | 381.076 |
|  | rs530804537 | -0.192 | 0.010 | 7.8001E-83 | A | G | 0.011 | 371.748 |
|  | rs55637835 | -0.019 | 0.003 | 8.40001E-09 | T | C | 0.121 | 33.186 |
|  | rs12739979 | -0.020 | 0.003 | 1.50003E-15 | T | C | 0.247 | 63.583 |
|  | rs72660548 | 0.051 | 0.008 | 5.50047E-11 | G | C | 0.018 | 42.992 |
|  | rs45613943 | -0.034 | 0.005 | 2.60016E-12 | C | T | 0.049 | 48.999 |
| ***NPC1L1*** |  |  |  |  |  |  |  |  |
|  | rs217399 | -0.021 | 0.002 | 5.50047E-24 | T | C | 0.444 | 102.029 |
|  | rs73107478 | 0.026 | 0.004 | 4.00037E-11 | C | A | 0.079 | 43.620 |
|  | rs11763759 | -0.013 | 0.002 | 0.000000012 | C | T | 0.304 | 32.526 |
|  | rs2073547 | 0.036 | 0.003 | 2.29985E-40 | G | A | 0.184 | 176.896 |
|  | rs148825701 | 0.017 | 0.003 | 1.20005E-11 | T | C | 0.212 | 46.003 |
|  | rs12666108 | 0.025 | 0.004 | 7.10068E-12 | C | T | 0.092 | 46.991 |
| ***APOB*** |  |  |  |  |  |  |  |  |
|  | rs4128553 | 0.060 | 0.003 | 2.70023E-77 | T | C | 0.120 | 346.284 |
|  | rs7557067 | -0.053 | 0.002 | 1.50003E-100 | G | A | 0.228 | 453.074 |
|  | rs72653066 | -0.073 | 0.010 | 1.50003E-13 | G | C | 0.011 | 54.581 |
|  | rs11901649 | 0.075 | 0.002 | 1E-200 | A | G | 0.444 | 1269.416 |
|  | rs114185526 | -0.096 | 0.010 | 3.80014E-22 | T | C | 0.011 | 93.631 |
|  | rs312961 | -0.019 | 0.002 | 1.59993E-18 | C | T | 0.366 | 77.075 |
|  | rs72782175 | -0.082 | 0.009 | 8.99912E-19 | C | T | 0.013 | 78.275 |
|  | rs137978266 | 0.071 | 0.010 | 1.9002E-12 | G | A | 0.012 | 49.574 |
|  | rs36134738 | -0.081 | 0.004 | 6.70039E-84 | G | A | 0.069 | 376.643 |
|  | rs76384951 | -0.021 | 0.004 | 0.000000017 | C | A | 0.083 | 31.810 |
|  | rs497166 | 0.075 | 0.008 | 2.19989E-23 | C | T | 0.980 | 99.289 |
|  | rs12720796 | 0.043 | 0.007 | 6.69993E-09 | C | A | 0.020 | 33.618 |
|  | rs10198175 | -0.047 | 0.003 | 5.19996E-43 | G | A | 0.891 | 189.035 |
|  | rs140798831 | -0.036 | 0.002 | 6.79986E-62 | C | T | 0.657 | 275.595 |
|  | rs149196850 | -0.071 | 0.010 | 1.59993E-13 | A | G | 0.012 | 54.504 |
|  | rs12713956 | -0.065 | 0.004 | 5.70033E-71 | G | A | 0.089 | 317.258 |
|  | rs72902590 | -0.080 | 0.005 | 1.20005E-57 | A | G | 0.045 | 256.158 |
|  | rs377122620 | 0.080 | 0.004 | 3.59998E-100 | G | T | 0.113 | 451.406 |
|  | rs10164442 | 0.028 | 0.002 | 1E-36 | G | A | 0.318 | 160.166 |
|  | rs1801701 | 0.039 | 0.003 | 8.60003E-30 | T | C | 0.099 | 128.539 |
|  | rs1042023 | 0.098 | 0.010 | 4.70002E-21 | C | G | 0.011 | 88.666 |
|  | rs1897083 | 0.076 | 0.002 | 1E-200 | G | A | 0.335 | 1195.213 |
|  | rs6756743 | 0.046 | 0.005 | 1.20005E-20 | T | C | 0.047 | 86.862 |
|  | rs10175646 | -0.044 | 0.006 | 4.30031E-15 | C | T | 0.965 | 61.575 |
|  | rs62122481 | 0.066 | 0.002 | 1E-200 | A | C | 0.377 | 933.125 |
|  | rs4665709 | -0.049 | 0.003 | 3.50026E-78 | A | G | 0.199 | 350.378 |
|  | rs533617 | -0.115 | 0.005 | 2.3014E-103 | C | T | 0.039 | 466.025 |
|  | rs12720807 | 0.063 | 0.003 | 2.19989E-82 | T | A | 0.118 | 369.652 |
|  | rs12720842 | 0.062 | 0.006 | 6.09958E-29 | C | T | 0.036 | 124.643 |
|  | rs531819 | 0.099 | 0.003 | 1E-200 | G | T | 0.850 | 1139.620 |
|  | rs540156 | -0.028 | 0.003 | 1.39991E-20 | T | C | 0.140 | 86.456 |

**Supplementary Figure S1A:** Leave-one-out permutation analysis of the causal association between LDL-C level and AS.

**Supplementary Figure S1B:** Leave-one-out permutation analysis of the causal association between HDL-C level and AS;

**Supplementary Figure S1C:** Leave-one-out permutation analysis of the causal association between TG level and AS;

**Supplementary Figure S1D:** Leave-one-out permutation analysis of the causal association between apoA-I level and AS;

**Supplementary Figure S1E:** Leave-one-out permutation analysis of the causal association between apoB level and AS;

**Supplementary Figure S2A:** Leave-one-out permutation analysis of the causal association between LDL-C level and AIS;

**Supplementary Figure S2B:** Leave-one-out permutation analysis of the causal association between HDL-C level and AIS;

**Supplementary Figure S2C:** Leave-one-out permutation analysis of the causal association between TG level and AIS;

**Supplementary Figure S2D:** Leave-one-out permutation analysis of the causal association between apoA-I level and AIS;

**Supplementary Figure S2E:** Leave-one-out permutation analysis of the causal association between apoB level and AIS;

**Supplementary Figure S3A:** Leave-one-out permutation analysis of the causal association between LDL-C level and CES;

**Supplementary Figure S3B:** Leave-one-out permutation analysis of the causal association between HDL-C level and CES;

**Supplementary Figure S3C:** Leave-one-out permutation analysis of the causal association between TG level and CES;

**Supplementary Figure S3D:** Leave-one-out permutation analysis of the causal association between apoA-I level and CES;

**Supplementary Figure S3E:** Leave-one-out permutation analysis of the causal association between apoB level and CES;

**Supplementary Figure S4A:** Leave-one-out permutation analysis of the causal association between LDL-C level and LAS;

**Supplementary Figure S4B:** Leave-one-out permutation analysis of the causal association between HDL-C level and LAS;

**Supplementary Figure S4C:** Leave-one-out permutation analysis of the causal association between TG level and LAS;

**Supplementary Figure S4D:** Leave-one-out permutation analysis of the causal association between apoA-I level and LAS;

**Supplementary Figure S4E:** Leave-one-out permutation analysis of the causal association between apoB level and LAS;

**Supplementary Figure S5A:** Leave-one-out permutation analysis of the causal association between LDL-C level and SVS;

**Supplementary Figure S5B:** Leave-one-out permutation analysis of the causal association between HDL-C level and SVS;

**Supplementary Figure S5C:** Leave-one-out permutation analysis of the causal association between TG level and SVS;

**Supplementary Figure S5D:** Leave-one-out permutation analysis of the causal association between apoA-I level and SVS;

**Supplementary Figure S5E:** Leave-one-out permutation analysis of the causal association between apoB level and SVS;

**Supplementary Figure S6A:** Scatter plot showing the association of the SNP effects on LDL-C level against the SNP effects on AS;

**Supplementary Figure S6B:** Scatter plot showing the association of the SNP effects on HDL-C level against the SNP effects on AS;

**Supplementary Figure S6C:** Scatter plot showing the association of the SNP effects on TG levels against the SNP effects on AS;

**Supplementary Figure S6D:** Scatter plot showing the association of the SNP effects on apoA-I level against the SNP effects on AS;

**Supplementary Figure S6E:** Scatter plot showing the association of the SNP effects on the apoB level against the SNP effects on AS;

**Supplementary Figure S7A:** Scatter plot showing the association of the SNP effects on LDL-C level against the SNP effects on AIS;

**Supplementary Figure S7B:** Scatter plot showing the association of the SNP effects on HDL-C level against the SNP effects on AIS;

**Supplementary Figure S7C:** Scatter plot showing the association of the SNP effects on TG levels against the SNP effects on AIS;

**Supplementary Figure S7D:** Scatter plot showing the association of the SNP effects on apoA-I level against the SNP effects on AIS;

**Supplementary Figure S7E:** Scatter plot showing the association of the SNP effects on the apoB level against the SNP effects on AIS;

**Supplementary Figure S8A:** Scatter plot showing the association of the SNP effects on LDL-C level against the SNP effects on CES;

**Supplementary Figure S8B:** Scatter plot showing the association of the SNP effects on HDL-C level against the SNP effects on CES;

**Supplementary Figure S8C:** Scatter plot showing the association of the SNP effects on TG levels against the SNP effects on CES;

**Supplementary Figure S8D:** Scatter plot showing the association of the SNP effects on apoA-I level against the SNP effects on CES;

**Supplementary Figure S8E:** Scatter plot showing the association of the SNP effects on the apoB level against the SNP effects on CES;

**Supplementary Figure S9A:** Scatter plot showing the association of the SNP effects on LDL-C level against the SNP effects on LAS;

**Supplementary Figure S9B:** Scatter plot showing the association of the SNP effects on HDL-C level against the SNP effects on LAS;

**Supplementary Figure S9C:** Scatter plot showing the association of the SNP effects on TG levels against the SNP effects on LAS;

**Supplementary Figure S9D:** Scatter plot showing the association of the SNP effects on apoA-I level against the SNP effects on LAS;

**Supplementary Figure S9E:** Scatter plot showing the association of the SNP effects on the apoB level against the SNP effects on LAS;

**Supplementary Figure S10A:** Scatter plot showing the association of the SNP effects on LDL-C level against the SNP effects on SVS;

**Supplementary Figure S10B:** Scatter plot showing the association of the SNP effects on HDL-C level against the SNP effects on SVS;

**Supplementary Figure S10C:** Scatter plot showing the association of the SNP effects on TG levels against the SNP effects on SVS;

**Supplementary Figure S10D:** Scatter plot showing the association of the SNP effects on apoA-I level against the SNP effects on SVS;

**Supplementary Figure S10E:** Scatter plot showing the association of the SNP effects on the apoB level against the SNP effects on SVS;

**Supplementary Figure S11A:** Funnel plot showing the association of the SNP effects on LDL-C level against the SNP effects on AS;

**Supplementary Figure S11B:** Funnel plot showing the association of the SNP effects on HDL-C level against the SNP effects on AS;

**Supplementary Figure S11C:** Funnel plot showing the association of the SNP effects on TG levels against the SNP effects on AS;

**Supplementary Figure S11D:** Funnel plot showing the association of the SNP effects on apoA-I level against the SNP effects on AS;

**Supplementary Figure S11E:** Funnel plot showing the association of the SNP effects on the apoB level against the SNP effects on AS;

**Supplementary Figure S12A:** Funnel plot showing the association of the SNP effects on LDL-C level against the SNP effects on AIS;

**Supplementary Figure S12B:** Funnel plot showing the association of the SNP effects on HDL-C level against the SNP effects on AIS;

**Supplementary Figure S12C:** Funnel plot showing the association of the SNP effects on TG levels against the SNP effects on AIS;

**Supplementary Figure S12D:** Funnel plot showing the association of the SNP effects on apoA-I level against the SNP effects on AIS;

**Supplementary Figure S12E:** Funnel plot showing the association of the SNP effects on the apoB level against the SNP effects on AIS;

**Supplementary Figure S13A:** Funnel plot showing the association of the SNP effects on LDL-C level against the SNP effects on CES;

**Supplementary Figure S13B:** Funnel plot showing the association of the SNP effects on HDL-C level against the SNP effects on CES;

**Supplementary Figure S13C:** Funnel plot showing the association of the SNP effects on TG levels against the SNP effects on CES;

**Supplementary Figure S13D:** Funnel plot showing the association of the SNP effects on apoA-I level against the SNP effects on CES;

**Supplementary Figure S13E:** Funnel plot showing the association of the SNP effects on the apoB level against the SNP effects on CES;

**Supplementary Figure S14A:** Funnel plot showing the association of the SNP effects on LDL-C level against the SNP effects on LAS;

**Supplementary Figure S14B:** Funnel plot showing the association of the SNP effects on HDL-C level against the SNP effects on LAS;

**Supplementary Figure S14C:** Funnel plot showing the association of the SNP effects on TG levels against the SNP effects on LAS;

**Supplementary Figure S14D:** Funnel plot showing the association of the SNP effects on apoA-I level against the SNP effects on LAS;

**Supplementary Figure S14E:** Funnel plot showing the association of the SNP effects on the apoB level against the SNP effects on LAS;

**Supplementary Figure S15A:** Funnel plot showing the association of the SNP effects on LDL-C level against the SNP effects on SVS;

**Supplementary Figure S15B:** Funnel plot showing the association of the SNP effects on HDL-C level against the SNP effects on SVS;

**Supplementary Figure S15C:** Funnel plot showing the association of the SNP effects on TG levels against the SNP effects on SVS;

**Supplementary Figure S15D:** Funnel plot showing the association of the SNP effects on apoA-I level against the SNP effects on SVS;

**Supplementary Figure S15E:** Funnel plot showing the association of the SNP effects on the apoB level against the SNP effects on SVS.
